# Community—Minimal Invasive Tissue Sampling (cMITS) using a modified ambulance for ascertaining the cause of death: A novel approach piloted in a remote inaccessible rural area in India

**DOI:** 10.1186/s13690-023-01062-x

**Published:** 2023-04-27

**Authors:** Ashish Satav, Niteen Wairagkar, Shubhada Khirwadkar, Vibhawari Dani, Reeta Rasaily, Usha Agrawal, Yagnesh Thakar, Dhananjay Raje, Fouzia Siraj, Pradyot Garge, Sameer Palaskar, Shraddha Kumbhare, Eric A. F. Simões

**Affiliations:** 1 Tahsil: Dharni, Mahatma Gandhi Tribal Hospital, MAHAN Trust, District Amaravati, Maharashtra State, Karmgram, Utavali 444702 India; 2 Community Medicine, MAHAN trust, Karmgram, Utavali, Dharni, District Amaravati, India; 3 Indian Council of Medical Research, New Delhi, India; 4 National Institute of Pathology, NIOP, New Delhi, India; 5 Center for Global Health, Colorado School of Public Health, University of Colorado School of Medicine, Aurora Colorado, USA; 6 Department of Paediatric Infectious Diseases, University of Colorado School of Medicine and Children’s Hospital Colorado, 12123 E 16Th Ave, Aurora, CO 80045 USA

**Keywords:** MITS, Community, Ambulance, VHWs, Counsellors

## Abstract

**Background:**

Melghat in India is a hilly, forested, difficult to access, impoverished rural area in northeast part of Maharashtra (Central India) with difficult healthcare access. Melghat has very high Mortality rates, because of grossly inadequate medical facilities. (1) Home deaths contribute to 67% of deaths,(2) which are difficult to track and where cause of death is mostly unknown.

**Methods:**

A feasibility study was carried out in 93 rural villages and 5 hospitals to assess feasibility of tracking real-time community mortality and to ascertain cause of death in 0–60 months and 16–60 years age group using Minimal Invasive Tissue Sampling (MITS) in purpose-modified ambulance. We used the network of village health workers (VHW)s, to establish real-time community mortality tracking. Upon receipt of reports of home death, we performed MITS within 4 h of death in the vicinity of the village.

**Results:**

We conducted 16 MITS. Nine, in MITS ambulance in community and seven at MAHAN hospital. The acceptance rate of MITS was 59.26%. Standard operating procedure (SOP) of conducting community MITS in an ambulance, is established. Major challenges were, Covid19 lockdown, reluctance of tribal parents for consent for MITS due to illiteracy, superstitions and fear of organ removal. Ambulance was an easy to reach transport means in remote area, provided a well-designed and discrete facility to perform MITS in community, winning the confidence of bereaved family. This has reduced time interval between time of death and performing MITS.

**Conclusions:**

MITS in purpose-modified Ambulance can be used worldwide for community MITS especially in areas which are remote and lack healthcare access. This solution needs to be assessed in different cultural settings to document culture specific issues.

**Supplementary Information:**

The online version contains supplementary material available at 10.1186/s13690-023-01062-x.

## Summary

Rural India has high mortality, mostly home deaths without diagnosis. To ascertain causes of deaths in 0-5 and 16-60-years group, we successfully conducted Minimal Invasive Tissue Sampling (MITS), nine in modified ambulance and seven in hospital. This method is replicable in low resource settings.

## Introduction

Global under 5 mortality rate (U5MR) and age standardized mortality rates were 38.42/1000 live births and 833·6 per 100,000 population of that age group, respectively in 2016 [1]. U5MR was 37·1 (33·2–41·7) in 2019 [2]. For a majority of these deaths, the cause of death (CoD) is unknown because of cultural barriers against performing a full autopsy and many deaths occur at home [3–5]. Child Health and Mortality Prevention Surveillance Network (CHAMPS) Program is a collaboration of research organisations in Africa and Asia developed to know the reasons of deaths using Minimal Invasive Tissue Sampling (MITS) as an substitute to complete post-mortem. CHAMPS will reinforce Global assessment of child mortality causes.

MITS improves specificity and provide a more complete opinion of the series of conditions leading to death, highlighting multiple probable interventions to prevent under-5 mortality and stillbirths [6]. The post-mortem MITS, is a method of tissue and body fluid sampling, integrated with advanced laboratory techniques for histopathology and molecular diagnostics that can inform CoD [6]. The procedure is quicker, less invasive, non-deforming, with less interval for the potential burial practices and therefore more culturally acceptable by parents in developing countries. It is cost effective than a complete diagnostic autopsy, and initial results have shown that MITS-informed CoD classification is equivalent to a complete diagnostic autopsy (Castillo, 2015) [7]. For stillbirths and intrauterine foetal deaths, the results of MITS are similar to standard autopsy[8].

MITS gives researchers, policymakers with excellent post-mortem data of accurate CoD needed to develop and execute particular interventions to prevent deaths to achieve Sustainable Development Goals (SDG) target by 2030 [9, 10]. Though, the CHAMPS Network has concentrated on children’s deaths, expanding the MITS concept to adults will furnish additional knowledge [10]. MITS is a reliable method to confirm CoD in HIV-infected patients with TB[11].

**Fig. 1:**
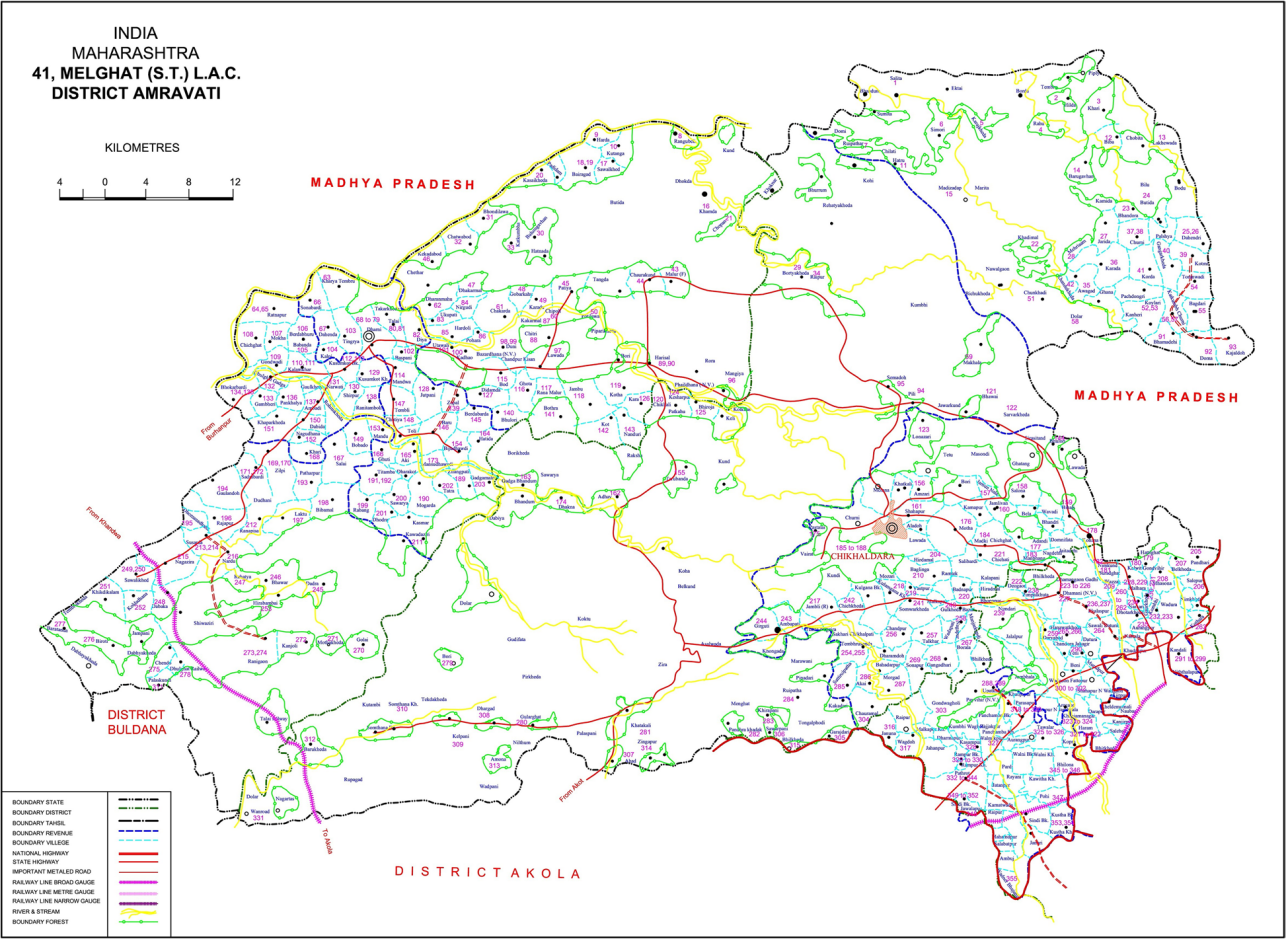
**(**Photo 1): Map of Melghat

**Fig. 2:**
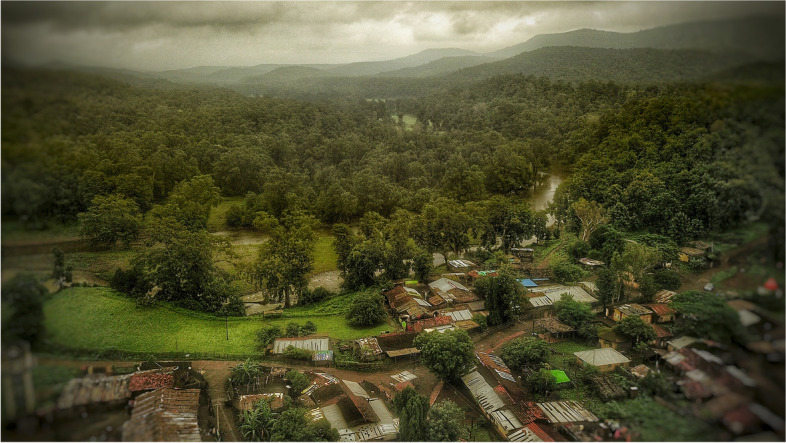
**(**Photo 2): Melghat Tribal village

Melghat is hilly, forested, difficult to access, impoverished rural area in northeast part of Maharashtra (Central India) with difficult healthcare access (Figs. 1 and 2). Melghat is spread over 4000 square kilometres with a population of 300,000 and a population density of 75/km^2^. Melghat has a very high U5MR of > 80/1000 live births, Infant Mortality Rate of > 55/1000 live births, Still Birth Rate of > 18/1000 live births and age specific mortality rate (16–60 years) > 400/100,000 population, because of grossly inadequate medical facilities and poor access to care [12]. The CoD is unknown in 67% of the deaths [13]. Majority (67%) deaths occur at home, not reported to government death registration and health system due to quick burial practices as culturally prescribed. Common causes for rejection of MITS by parents are social and old style doubts in India and burial delays in Pakistan. All parents from India and Pakistan were interested to know the cause of children’s deaths [14].

The objective of the study is to know the causes of deaths by using purpose modified ambulance in difficult to access, hilly forest tribal areas of Melghat.

We piloted a novel approach of reaching the unreachable to carry out MITS in community (cMITS) using purpose modified ambulance, trained team of technicians and doctors to carry out cMITS to track community mortality and ascertain cause of death.

We have established a system of real-time tracking of community mortality (deaths at home in community) since last 15 years through a network of trained village health workers (VHWs) who live in villages establishing trust with community [15]. This system facilitated MITS pilot project. In rural Melghat, full autopsy is culturally unacceptable.

In many of the LMICs with difficult to reach areas lacking healthcare access and significant number of home deaths, community-MITS in ambulance approach will be helpful to understand cause of deaths, obtain data on population-based burden of mortality to frame appropriate policies for reduction of high mortality in underserved communities.

Hence one of the objectives is to develop replicable model for other similar geographies.

In any case, the difficult to access terrain, precludes taking the dead body to a hospital for full autopsy to ensure a timely post-mortem evaluation.

In the CHAMP sites, MITS studies are usually carried out in tertiary care hospitals [16]. In many low and middle-income countries (LMICs) with difficult to reach areas lacking healthcare access and significant number of home deaths, a community-MITS in ambulance approach could help to understand CoD, and thus obtain data on population-based burden of mortality to frame appropriate policies for reduction of high mortality in these underserved communities.

Thus, we document our experience in this paper so that our innovative approach could be piloted in other similar geographies.

## Methods

This pilot study aims to assess feasibility of a novel approach of cMITS to identify accurately, the causes of stillbirths, neonatal deaths, and deaths in (1–60 months and 16–60 years age group) in 93 villages (with population of 101,792), four government hospitals and the MAHAN (Meditation, AIDS, Health, De-Addiction, Nutrition) hospital in Melghat, Central India over one year from January 1^st^ 2020 to December 31^st^, 2020. MAHAN is a charitable trust providing medical services to highly impoverished, difficult to access, hilly, forest, tribal area of Melghat in central India for 25 years.

### Eligibility criteria

#### Inclusion criteria

Still births and all deaths amongst Age 0 to 5 years, 16–60 years, in usual resident population of 93 villages of Melghat and MAHAN base- hospital, after obtaining informed consent from parents/guardians or next of kin of deceased person.

#### Exclusion criteria

Deaths in families, planning to migrate from the study area within next three months, prisoners, accidental deaths and where informed consent could not be obtained from decisionally-challenged parents, unwilling caretaker/ family member.

Approvals were obtained from Institutional independent review board of MAHAN trust; Ethical, technical, and administrative committees of state government of Maharashtra and Health Ministry Screening committee of Government of India for piloting community MITS project using a purpose-built ambulance in a rural area of Melghat.

#### Obtaining community and individual consent

Community participation and acceptance is critical in community-based studies. The study team, after seeking permission from the traditional panchayat (village elders with at least 60% of all adults in the village in attendance), approached the whole village to explain the study and obtained verbal and written community consent to the study before starting the study. After that, we selected local trained semiliterate female VHW, accepted by the community, for each village. A health supervisor was also identified to supervise 8–10 of the VHWs and their assigned villages. The VHWs used a baseline census to identify all children under five years of age and adults in age group of 16–60 years in the villages. After obtaining a signed, written informed consent from the caretaker, near relatives, parent or guardian, a socio-economic and demographic questionnaire was administered.

#### Mortality monitoring & Strategies for MITS

For the last 15 years, we have established a real-time community mortality tracking system through our VHWs and counsellors, who are local tribal working in villages and in government hospitals respectively. As our VHWs live in the same village and have been providing medical services for several years, they have good community rapport, are informed of most community deaths within 4 h, which they report back to MAHAN. We have conducted 2000 verbal autopsies since 2004. Our counsellors also working round the clock, in all public hospitals, have been helping tribal people for the last 14 years, strengthening hospital services and have good rapport with the local people and hence pick up most of the hospital deaths within 2 h as well [17].

Community sensitization and mobilization activities were carried out from January 1^st^ to December 31^st^, 2020. Our team explained the importance of MITS to community elders and requested them for their support. MITS support groups were developed in villages. Traditional faith healers and traditional birth attendants were motivated to be part of the project and help obtain informed consent from relatives of deceased person. Focus Group Discussion with stakeholders and target groups and behaviour change communication through VHWs were carried out for explaining importance of MITS. The counsellors tracked every serious patient in age group of 0–5 years and 16–60 years admitted in the hospitals and reported the deaths to MITS research team. Our VHWs and counsellors are trained in grief counselling by qualified clinical psychologist [Annexure 1]. There is significant difference between grief counselling and MITS consent. The grief counselling is offered to console the relatives of the dead person who are in psychological trauma. It has no objective to coerce the relative to give consent. Grief counselling was offered to all families where a death happened irrespective of whether they consented for MITS or not. Our team is doing grief counselling since 2016, long time before MITS project as part of our community work. To know certainly the cause of death (CoD) to protect their future children was the major stimulus for involvement and consenting by parents in MITS. Correct pre-consent information of MITS procedures via suitable grief counselling is vital for decision-making by parents [18].

#### MITS pro cedure area

As 67% of the deaths occur in villages, an ambulance was the main site for MITS. We also developed a special MITS room at the MAHAN base hospital.

#### MITS ambulance set-up

**Fig. 3:**
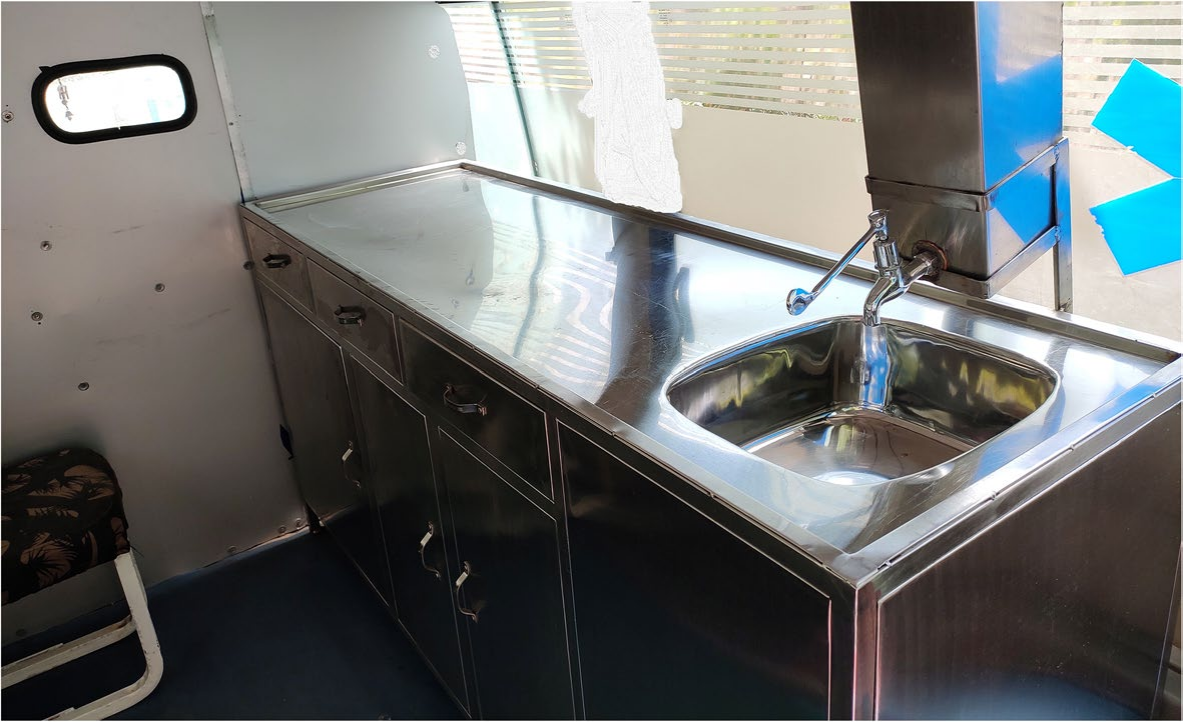
**(**Photo 3): MITS table in MITS ambulance

**Fig. 4:**
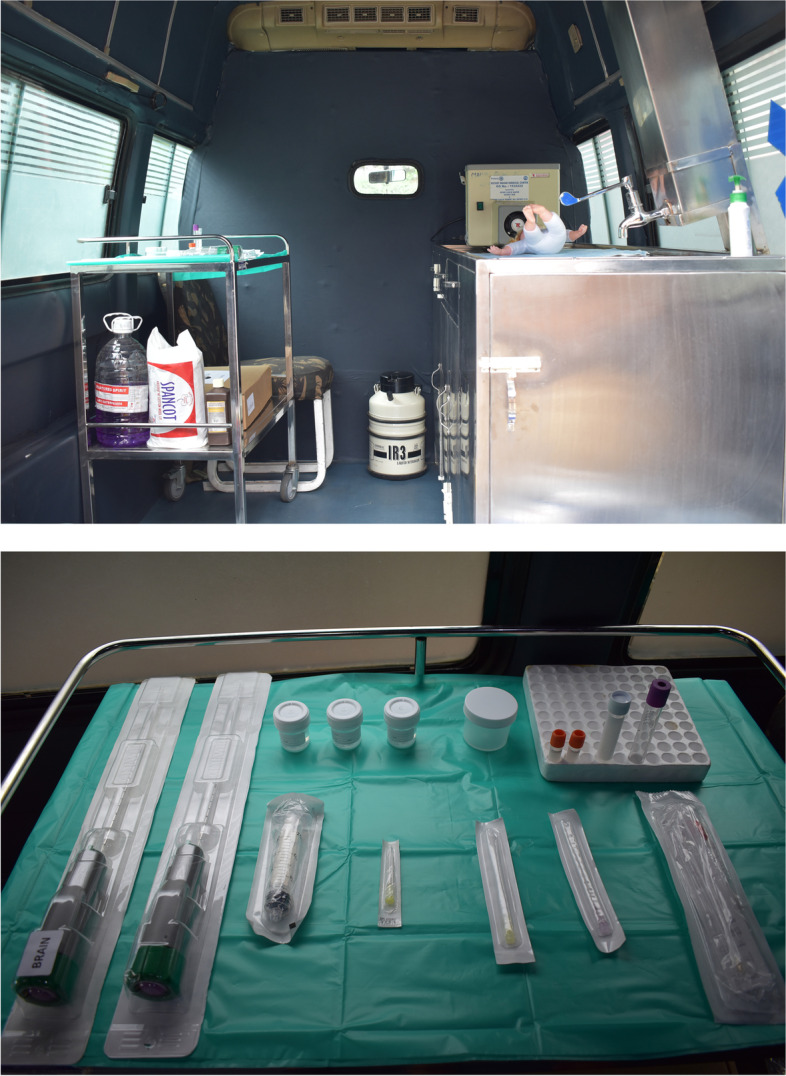
**(**Photo 4): Arrangement of kits in MITS ambulance

A regular ambulance was modified to suit the performance of MITS. All surfaces of ambulance were made airtight and as smooth as possible to prevent dust accumulation, thus reducing chances of contamination. Adequate light sources and ventilation was maintained with an air conditioner. Sterilization was carried out as per standard operation theatre guidelines [Annexure 2]. A MITS table was installed with a wash basin, adequate hand-washing, and drainage area as per guidelines (Fig. 3). There are clearly delineated spaces for storing materials and for donning and doffing of personal protective equipment [Annexure 3] (Fig. 4). This ambulance was not used for any other patient transport. It was kept in a ready to go state 24 × 7 to minimize duration between time of death and cMITS.

The trained site surveillance team (TSST) which comprised of VHW, supervisor or counsellor, was authorized to seek informed consent from the relatives. If the relatives are not literate, then the procedure was explained in local tribal dialect in presence of literate witness, who were not part of the TSST. Literate witness also counter signed the consent form along with thumb impression of illiterate parents. The consent form was in Hindi language. We also obtained audio consent whenever needed and is stored.

#### Community mortality tracking

**Fig. 5:**
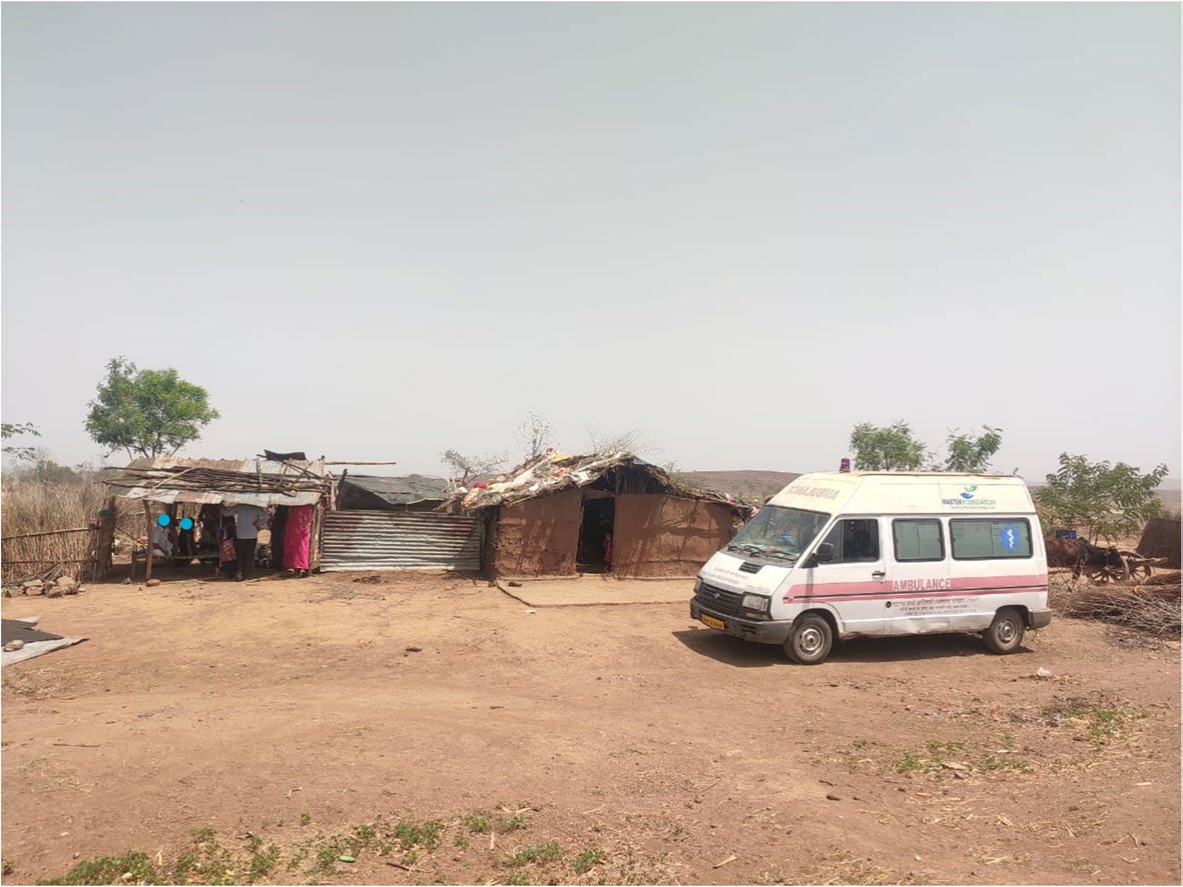
**(**Photo 5): MITS ambulance Infront of tribal hut in village

A child (0–5 years) or adult (16–60 years) death is identified in the community by VHWs or in hospital by counsellors and reported to MAHAN network. Trained site surveillance team (TSST) (VHW, supervisor or counsellor) approached families for grief counselling. Our team offered grief counselling to all families experiencing deaths. After grief counselling and once the mental status of the relatives is stabilised, the parents/near relatives/ caregivers were requested by TSST to sign an informed consent form (in local language) for collecting MITS samples. TSST informed the death within 30 to 60 min to MAHAN base hospital by phone or personal visit. For eligible deaths, notified within the MITS timeframe (within 24–36 h of death), MAHAN- MITS team reached to site by MITS ambulance (Fig. 5). On getting informed consent by TSST, MAHAN MITS-experts and MITS-assistant conducted MITS in an ambulance or hospital [Annexure 4]. Most of the MITS were conducted within 4 h of deaths and the timing needed for actual MITS procedure were 60–75 min. The doctors and MITS team also did counselling. Doctors, counsellors and MITS team must do expert counselling to improve consent for MITS [19].

Important concept in consenting by persons are the hope of some assistance for involvement and their remarkably high levels of faith in the researchers [20].

#### Conduct of MITS

The TSST obtained the informed consent for MITS. TSST was responsible for getting informed consent. After obtaining written informed consent, the MAHAN- MITS team reached the site by MITS ambulance (Fig. 5), where they conducted MITS in either the ambulance or hospital if the person died there [Annexure 4]. MITS samples were collected from the lung, brain, liver, cerebrospinal fluid (CSF), blood, and nasopharyngeal swabs as well as from placenta and umbilical cord for stillbirths or early neonatal deaths.

#### Sample transportation

Samples were transported to MAHAN laboratory within 1–2 h. PCR samples were preserved in cryo-vials at -160^0^C in liquid nitrogen and histopathology samples in formalin [Annexure 5]. Subsequently when enough samples are collected, they were transported following standard procedures from MAHAN hospital to National Institute of Pathology- Indian Council of Medical Research New Delhi for histopathology analysis, to National Institute of Virology Pune for PCR, and for routine microbiology and culture to Vishakha Clinical Microbiology laboratory, Nagpur [Annexure 5].

#### Verbal Autopsy (VA)

A supervisor and VHW verified the deaths and conducted a VA within 2 weeks of deaths. Details regarding the circumstances surrounding the death was recorded. The VA consists of a standardized, validated questionnaire with local terminologies. The investigators having over 15 years of experience in conducting verbal autopsies have developed and used standardized protocols modified by combination of Society for Education, Action and Research in Community Health (SEARCH) a non-governmental organisation’s instrument, [21] the WHO-VA forms, [22] the IHME validated protocol, [23] and the CHAMPS VA forms for under 5 children [24]. For adults, a separate VA instrument was developed by MAHAN which is modified WHO VA form. Standard algorithms were used for determining major identifiable causes of deaths. We used WHO consensus development techniques to synthesize 2 experts’ opinion on diagnostic criteria for identification of causes of deaths.

#### Data collection

Available anthropometric measurements, demographic data, clinical records, VA data were entered in project database. Data was collected by field team and data entry done by a data entry operator on password protected access databases in the MAHAN office. Data were double entered (once in access and once in excel sheet) and verified by a data manager to minimize data errors.

On obtaining the necessary data and determining the cause, [Annexure 10] parents and relatives were informed about the cause of death. The cause of death was determined through verbal autopsy and MITS results in the form of microscopy, blood culture, malaria and HIV rapid test, ELISA test, histopathology and PCR of lung, liver and brain tissues. Cause of death was determined by a panel of experts which consisted of onsite physician with research experience of 25 years, paediatrician, microbiologist, pathologist, epidemiologist, infectious disease expert, public health expert and global health expert. Details have been explained in annexure 10.

It was envisaged to perform MITS in 50 deaths during the feasibility assessment period August 2019 to July 2020.

#### Diagnosis standards are as per CHAMPS data elements [17]

### Results

This manuscript is a description of the methodology for doing MITS in the ambulance and not the results.

During the study period, (1^st^ May 2020 to 30^th^ April 2021) there were 32 deaths which were eligible for MITS**,** which the MITS team was informed. Of those**,** MITS was not conducted in 16 deaths i.e., 50%. We could approach 27 deaths but our team could not reach in time in 5 deaths as the funeral was done immediately after death. We could obtain informed written consent for MITS from relatives of 16 dead persons (59.26%). This might introduce some bias in ascertaining cause of death as well as in cause wise distribution of deaths in community. In a sensitive and very difficult community study, 50% achievement of the target is reasonable.

We conducted MITS on 16 of 27 deceased persons (59%) of families approached. The average time between death and sample collection was 2 h, 29 min. Tables 1 and 2 revealed that, of 16 deaths, there were 2(13%) still births, 5(31%) neonatal deaths, 2 (13%) post-neonatal children’s deaths and seven (44%) deaths in 16–60 years age group. The male to female ratio was 1.3:1.

**Table 1:** Socio-demographic data on deaths investigated (*n* = 16) (1^st^ May 2020 to 30^th^ April 2021) (Dharni Block of Amaravati district)

Parameter	Categories	(Number)	(%)
Age category	Still births	2	12.5
	0 – 28 days	5	31.25
	29 days – 5 years	2	12.5
	16 years– 60 years	7	43.75
Sex	Male	9	56.25
	Female	7	43.75
Place of death	SDH^a^ Dharni (dead bodies were shifted to villages.)	7	43.75
	MAHAN hospital	7	43.75
	Village	2	12.5
Place of MITS^b^	Ambulance	9	56.25
	MAHAN hospital	7	43.75

^a^*SDH*  Sub district hospital

^b^*MITS* Minimal invasive tissue sampling

**Table 2:** Age distribution of MITS cases (1^st^ May 2020 to 30^th^ April 2021) (Dharni Block of Amaravati district)

Age group	Total deaths	Number (No.) of MITS done			MITS not done (No.)	Cause of non-performance
		Total	In hospital	In ambulance		
Still births	6	2	0	2	4	a) Our team could not reach in time in 1 death as funeral was done immediately after death b) Our team could not obtain consent from relatives of 3 deaths
0 – 28 days	11	5	0	5	6	a) Our team could not reach in time in 3 deaths as funeral was done immediately after death b) Our team could not obtain consent from relatives of 3 deaths
29 days – 5 years	5	2	0	2	3	a) Our team could not reach in time in 1 death as funeral was done immediately after death b) Our team could not obtain consent from relatives of 2 deaths
16 years– 60 years	10	7	7	0	3	a) Our team could not obtain consent from relatives of 3 deaths
Total	32	16	7	9	16	a) Our team could not reach in time in 5 deaths as funeral was done immediately after death b) Our team could not obtain consent from relatives of 11 deaths

Table 3 shows that the clinical causes of deaths for three (18.75%) cases were pneumonia, while four (25%) cases had COVID-19, acute respiratory distress syndrome (ARDS), hypoxia. The microbiological analysis of CSF showed five (31.25%) positive cases (three staphylococcus aureus, one klebsiella pneumonia and, one Escherichia coli), while blood culture of one case was positive for alpha haemolytic streptococci. One case each of HIV, Malaria and Dengue were confirmed. MITS PCR of nasal swab and lung tissue revealed that Klebsiella pneumoniae is major cause of infant mortality (mostly new-born) seen in 5 cases and H. Influenzae and Staphylococcus aureus is seen one case each.

**Table 3:** Causes of deaths (1^st^ May 2020 to 30^th^ April 2021) (Dharni Block of Amaravati district)

a: Causes of deaths (Without PCR)										
Parameter			Categories				Number			%
Sample collection [16]			Cryovial lungs				16			100
			Cryovial brain				16			100
			Cryovial liver				7			43.75
			CSF^a^				16			100
			Nasopharyngeal swab				14			87. 5
Clinical cause of death			Pneumonia				3			18.75
[16]			COVID-19, ARDS^b^, Hypoxia				4			25
			Still birth				2			12.5
			Brought dead				2			12.5
			Dengue				1			6.25
			Unknown				4			25
CSF Culture [16]			Staphylococcus Aureus				3			18.75
			Klebsiella Pneumonia				1			6.25
			Escherichia coli				1			6.25
Blood Culture [14]			Alpha Haemolytic Streptococcus				1			7.14
			No growth				13			92.86
HIV^c^			Positive				1			6.25
Malaria			Vivax positive				1			6.25
Dengue			Positive				1			6.25
**b: The link between clinical cause of death and MITS cause of death, to show the added value of MITS**										
**S. No**	**MITS KIT ID. No**	**AGE (Days** **/Months** **/ Yrs.)**	**Immediate cause of death**	**Blood culture (growth after 5 days)**	**CSF culture**	**HIV**	**Malaria**	**Dengue**	**Nasal swab PCR**	**Lung PCR**
1	108–002	1 day	Aspiration Pneumonia	No growth		Negative	Negative	N/A	K. pneumoniae	Negative
2	108–003	1.5 month	Pneumonia	No growth		Negative	Negative	N/A	NA	M. catarrhalis
3	108–004	still birth	Still birth	No growth		Positive	P. Vivax (Positive)	N/A	Negative	Negative
4	108–005	6 days		No growth		Negative	Negative	N/A	K. pneumoniae	K. pneumoniae
5	108–006	19 years	Brought death		Klebsiella pneumonia	Negative	Negative	Positive	NA	Negative
6	108–007	2 days		No growth	Klebsiella Pneumonia	Negative	Negative	N/A	Negative	K. pneumoniae
7	108–008	45 years	Dengue	No growth	No growth	Negative	Negative	N/A	Negative	Negative
8	108–009	still birth	Still birth	No report	No report	Negative	Negative	N/A	S. aureus	Negative
9	108–010	1 month		No growth		Negative	Negative	N/A	Negative	Negative
10	108–011	2 Day		No report	No report	Negative	Negative	N/A	K. pneumoniae	K. pneumoniae
**S. No**	**MITS KIT ID.No**	**AGE (Days** **/Months** **/ Yrs.)**	**Immediate cause of death**	**Blood culture (growth after 5 days)**	**CSF culture**	**HIV**	**Malaria**	**Dengue**	**Nasal swab PCR**	**Lung PCR**
11	108–012	45 years	Brought death	Alpha Haemolytic Streptococcus	No growth	Negative	Negative	N/A	Negative	Negative
12	108–013	11 months	Pneumonia	No growth	Staphylococcus Aureus	Negative	Negative	N/A	Rhinovirus + K. pneumoniae	Negative
13	108–014	65 Year	Covid-19 (positive)	No growth	Staphylococcus Aureus	Negative	Negative	N/A	Negative	Negative
14	108–015	18 years	Covid ??	No growth		No report	No report	N/A	H. influenzae	Negative
15	108–016	55 years	ARDS with severe Hypoxia			Negative	Negative	N/A	NA	Negative
16	108–017	70 years	ARDS with severe Hypoxia			Negative	Negative	N/A	NA	Negative

^a^*CSF*  Cerebrospinal fluid

^b^*ARDS* Acute respiratory distress syndrome

^c^*HIV*  Human immunodeficiency syndrome

The histopathology, and VA are in progress and will be presented in a separate manuscript.

## Discussion

We could obtain informed written consent for MITS from relatives of 16 dead persons (59.26%). This might introduce some bias in ascertaining cause of death as well as in cause wise distribution of deaths in community. In a sensitive and very difficult pioneering community study like MITS, 100% achievement of the target is not possible. Our study, community MITS in ambulance is done for the first time in the world. Areas with high community mortality where cause of death is not known in most of the cases, community MITS is the only alternative.

### Our MITS experiences

Tribal culture and beliefs did not allow shifting dead bodies to hospital for any investigations. Local tribal prefer to conduct funeral at the earliest after death. A modified ambulance was sent in the village, community taken into confidence, explained the procedures and invited to watch MITS if parents/relatives were willing. Allowing a relative to observe MITS had enhanced community’s confidence and led to increased cooperation for procedures and allay their fears about the organs being removed for malicious purposes. Ambulance MITS were found to be more acceptable than hospital MITS. It was after conducting 16 MITS in Melghat by MAHAN team. All of the relatives preferred MITS in ambulance rather than shifting the body to hospital for MITS.

### Challenges encountered

Tribal are usually reluctant for procedure like autopsy or sample taking post-mortem due to cultural beliefs that departed souls should not be disturbed on their journey to another world. Illiteracy, superstitions, and fear of organs being removed during such procedures also affected community’s acceptance of MITS. This was observed in one out of 16 MITS cases done in our study.

Long duration of MITS procedures created problems and doubts in mind of tribal.

The basis of the descriptive findings is focus group discussions (FGD). The instrument for the focus group discussion was developed after a meeting with experts including a social scientist, paediatrician, physician practising in Melghat for more than 25 years, program manager/public health expert, local tribal VHW and a local tribal representative. Community-based focus group discussions (FGDs) were conducted using this instrument in 8 representative villages of Melghat, to understand the causes, places of deaths, their views about rituals and life after death; and their concepts, inhibitions and challenges regarding autopsy when it was done for medicolegal cases in hospital [13].

Each focus group consisted of 5 to 10 tribal males and females participants. Members conducting FGD consisted of program manager, specially trained MITS experts, village health worker, community mobiliser and counsellor. The team explained the importance of autopsy, difference between routine autopsy and MITS, and advantage of MITS over autopsy. The results obtained from focus group discussions regarding autopsy, MITS, socio-cultural aspects including challenges, beliefs, cultural practices were compiled [13].

A detailed analysis of the FGD is beyond the scope of this manuscript, to quantify the statements.

Initially in two instances few community members instigated parents against us and complained to local administration. Some political persons misguided community against MITS.In one still birth, our MITS experts took placenta for analysis. The mother-in-law became very angry because of the belief that if placenta is not buried in home, the woman will not conceive in future. After such incidences, we conducted serial meetings with parents and key persons in villages and explained the facts and removed myths from minds of tribal, invited parents/relatives/ community leaders to observe performance of MITS in ambulance.

MAHAN trust has very strong community network and community rapport, good relationship with government officers and well-trained staff to handle such incidences. Principal investigator and program manager talked to government officers and local administration, revealed all the permission granted by government, ethical committee, consent from parents of dead children and community for conducting MITS to the officials. Our team explained importance of community MITS to the officers and community members. Government officers, community members and family of the dead persons were convinced with our good intensions and benefit to the society and the issues were resolved.

Such education and transparency led to enhanced confidence about our objectives and subsequent MITS performances were uneventful. Poor connectivity and transportation facilities, posed difficulty in getting immediate information of death.

Maharashtra state government did not approve hospital MITS in government hospital of Dharni. Dharni is the tahsil/block in Amaravati district of Maharashtra state of India.

After hospital deaths, dead bodies were handed over to relatives for further rituals and funeral. Our team could conduct community-MITS in ambulance in the villages after due consent. During COVID lockdown, local administration prohibited us to conduct MITS in community.Keeping the community motivated for MITS is great challenge. It remains to be seen if other cultures would encounter such challenges as we faced. We had established a good rapport with the community for last 25 years providing free/very affordable medical services to tribal from our study area. Our network of VHW working in same area for long time proved to be an asset for the program, establishing trust with the community. Our trained grief counsellors and grief counselling procedures immediately after the death added value for the program. On follow-up discussions with community, it was felt that the desire to know the cause of death led to giving informed consent. Our reporting back to parents about cause of death helped to enhanced cooperation. We have experience of surveys of the tribal community in Melghat from 1998 to 2020. Impact of our VHWs and counsellors have been published before this paper [12, 25–29]. The above statements are after qualitative analysis of our various surveys, multiple FGDs and key informant interviews, with the community members, relatives of the deceased persons and relatives of dead person where MITS have been conducted.

Proper counselling of the parents and family, based on sociocultural and religious grounds along with the good care, behaviour and empathetic conversation by the healthcare providers can improve acceptability of MITS[30]. Above descriptive findings are not anecdotal.

### Advantages of ambulance-MITS

The responses of the community members were quite positive towards ambulance MITS as ambulance was reaching to their doorsteps. It avoided cost and efforts of relatives for travelling to hospital for MITS, it also saved their lost wages which would have happened for hospital MITS. They were allowed to see MITS in ambulance if willing, which built their confidence in our procedure and intensions. They were happy as they could know the cause of death free of cost at door step, which will help them to prevent further death. We understood this after focus group discussions with relatives of the dead person after MITS.

Due to ambulance, the response time for MITS after reports of death at home was short, it was easy in reaching out the remote, inaccessible area. It had reduced the time interval between deaths and MITS (Average 2 h,29 min). It has overcome the cultural barrier of refusal of shifting dead body to hospital for MITS. Our MITS ambulance is secure place for collection and appropriate storage of samples in community e.g., Liquid nitrogen container for storage of PCR samples in cryovials at less than minus 160^0^C.

Transparency imparted by MITS conducted in full view of relative, yet discretely, removed fear about organ removal and boosted community acceptance. Our program manager and coordinator conducted focus group discussions with the relatives of the dead person, couple of days after MITS. Our team also conducted meeting with various community members after MITS. Our study team determined it after analysis of above FGD.

Modified ambulance is kind of well-resourced mobile mortuary facility in community. Ambulance can be used for shifting serious patient from village to hospital or dead body from hospital to village. During travel, our team got enough time to understand the circumstances causing deaths and emotions of the relatives, developed good rapport with the relatives of the dead person, could do grief counselling and explain our intentions of MITS. Hence it becomes easy to obtain the informed consent of the relative for ambulance MITS. Our team did not force the relatives for consent. Our team got permission from independent ethical committee and health ministry screening committee of government of India for all of the procedures mentioned in the manuscript. Proper counselling of the parents and family, based on sociocultural and religious grounds along with the good care, behaviour and empathetic conversation by the healthcare providers can improve acceptability of MITS [30]. To know surely the cause of death (CoD) to guard their upcoming children was the key incentive for participation and consenting by parents in MITS. Correct pre-consent information of MITS procedures via suitable grief counselling is vital for decision-making by parents [18].

As per our preliminary cost analysis, Ambulance MITS has reduced the cost of MITS by avoiding development of MITS infrastructure in 17government hospitals. There are 17 government hospitals and one MAHAN hospital where MITS was supposed to be conducted. Which required building up of the MITS infrastructure in 18 hospitals which would have increased the cost by USD 42,385. Single MITS ambulance has reduced the cost significantly which was USD 12,573 while the cost of developing MITS infrastructure in 17 hospitals would have been USD 54,958. This study addressed a significant gap in addressing causes of community mortality and established a novel potential solution of community MITS. Further experience in other cultures and adopting cMITS ambulance procedure to the culture will remove the barriers in ascertaining the age specific population-based mortality data. To our knowledge this is the first study in world where MITS was conducted in community utilizing ambulance to ascertain the cause of deaths. It will help policy makers to plan programs to obtain reliable causes of deaths and frame policies to reduce deaths in age group of 0–5 years and 16–60 years in most impoverished area of world.

There were certain limitations and challenges we encountered**.** It was difficult to obtain MITS for deaths occurring at night, given tribal cultural practices of rapid burial. Before our MITS study, it never happened that an ambulance came to a village to escort a dead body except in medico-legal case. Even if it happened, it never happened that the dead body was kept in the ambulance for one hour, some procedure was done on the dead body inside the ambulance and then the body was handed over to the relatives. It was felt that we may need to give more time to the family and community to be prepared for such an experience.

All of the challenges or limitations were after qualitative analysis of the FGDs, key informant interviews, multiple surveys of the communities, multiple discussions with community members, government service providers and voluntary organisations, and practical experience of our research staff who conducted many research studies in the same tribal communities from 1998 to 2020 and research publications [12, 13, 31].

Since the ambulance was not custom-built for the purpose but was a modified, converted version of the locally available vehicle, we felt the space constrains for performing MITS in community setting. We would need additional, partitioned area for MITS, donning/doffing area and secluded place for relatives, all in one ambulance. Such partition will act as barrier room for MITS area, further improving sterilization of working place. Design considerations will improve the experience across the geographies and cultures.

At present there is no evidence of conducting post-mortems in remote inaccessible areas to know the cause of death. In such areas, post mortems are conducted only in medico legal cases in LMIC. Hence it is our hypothesis that this MITS approach could perhaps be extended for post-mortems to know the causes of deaths in remote inaccessible areas." This is the first community-based feasibility/pilot study conducted to know which are the probable interventions to know the causes of deaths in case of home deaths in remote inaccessible areas of LMIC with scarce health services where causes of deaths are not known in 67% of deaths [13, 29].

## Conclusions

We piloted a novel approach for ascertaining cause of death in remote inaccessible areas. MITS in Ambulance approach can be used worldwide for introduction of community-MITS for investigations of home deaths, especially in difficult to reach areas. This approach could perhaps be extended for general post-mortems in remote inaccessible areas. More research and more such pilots would be needed before generalization of this concept across cultures and geographies.

## Supplementary Information

**Additional file 1:** Annexure 1: Standard operating procedure (SOP) for grief counselling MITS in MAHAN.

**Additional file 2:** Annexure 2: MITS Ambulance sterilisation SOP.

**Additional file 3:** Annexure 3: MITS Ambulance Set up.

**Additional file 4:** Annexure 4: Minimally Invasive Tissue Sampling SOP MAHAN.

**Additional file 5:** Annexure 5: SOP for sample storage and transport.

**Additional file 6:** Annexure 6: Verbal autopsy form for neonatal deaths (0 to 28 days).

**Additional file 7:** Annexure 7: Verbal autopsy form of post neonatal under 5 children’s deaths.

**Additional file 8:** Annexure 8: Verbal autopsy form for death of people in age group of 16-60 years.

**Additional file 9:** Annexure 9: Tangerine Software.

**Additional file 10: **Annexure 10: COD procedure.

**Additional file 11.**

## Data Availability

**Table:** 

Will individual participant data be available (including data dictionaries)?	No, as per guidelines of the Government of India (GOI)
What data in particular will be shared?	Aggregate data that underlie the results reported in this article, after de-identification (text, tables, figures, and appendices)
What other documents will be available?	Study protocol
When will data be available (start and end dates)?	Beginning 9 months and ending 36 months following article publication
With whom?	Investigators whose proposed use of the data has been approved by an independent review committee, the GOI and ethical review by the ICMR and Govt of Maharashtra (India), Tribal Section clearance, identified for this purpose
For what types of analyses?	For meta-analysis
By what mechanism will data be made available?	Proposals may be submitted up to 35 months following article publication. After 36 months the data will be available with investigator support only after permission from government of India

## Abbreviations

MITS: Minimal Invasive Tissue Sampling
VHWs: Village Health Workers
SOP: Standard operating procedure
CoD: Cause of death
CHAMPS: Child Health And Mortality Prevention Surveillance (Network)
SDG: Sustainable development goals
cMITS: Minimal Invasive Tissue Sampling in Community
LMIC: Low and middle-income countries
ARDS: Acute respiratory distress syndrome
CSF: Cerebrospinal fluid
