# Supplementary material for: Community—Minimal Invasive Tissue Sampling (cMITS) using a modified ambulance for ascertaining the cause of death: A novel approach piloted in a remote inaccessible rural area in India
Source: Arch Public Health. 2023 Apr 27;81:72. doi: 10.1186/s13690-023-01062-x (PMC10134564; doi:10.1186/s13690-023-01062-x)
Supplement: Supplementary file 1 — Additional file 1: Annexure 1: Standard operating procedure (SOP) for grief counselling MITS in MAHAN. [file 13690_2023_1062_MOESM1_ESM.pdf]

SOP of Grief Counselling for MITS

(1st May 2020 to 30th April 2021) (Dharni Block of Amaravati district)

1.Purpose

The purpose of this Standard Operating Procedure (SOP) is to describe the procedure for Grief counseling of caretakers of dead child, before specimen collection during the MITS procedure.

What is grief counseling? The counseling offers friendly talk, needful advice, emotional support, discussion and sharing knowledge about ways to tackle the situation. It should be natural, to the point, showing right path to relatives, with good faith and caring.

There is significant difference between grief counselling and MITS consent. The grief counselling is offered to console the relatives of the dead person who are in psychological trauma. It has no objective to coerce the relative to give consent. We offered grief counselling to relatives of all dead persons, whether they give consent or not for MITS. Our team is doing grief counselling since 2016, long time before MITS project as part of our community work.

Common causes for denial of MITS by parents are social and traditional apprehensions in India and burial postponements in Pakistan. All parents from India and Pakistan were inquisitive to know the cause of children deaths.[1]

Acceptability of MITS can be improved by sociocultural and religious aspects based appropriate counselling of the parents and family along with the quality of care, attitude and communication by the healthcare providers.[2]
Expert counselling by MITS and counselling team along with doctors is must to improve consent for MITS.[3]

Essential themes in consenting by persons are the expectation of some benefits for contribution and their unusually high levels of trust in the researchers.[4]

To know certainly the cause of death (CoD) to protect their future children was the major stimulus for involvement and consenting by parents in MITS. Proper pre-consent knowledge of MITS procedures via appropriate grief counselling is important for decision-making by parents.[5]

The effects of death of child on parents/caretakers can be devastating. There are following 5 stages of reaction from family.

1. Denial /Rejection.
2. Anger.
3. Bargaining
4. Depression and guilt feeling.
5. Acceptance of the situation.

Above emotions can be different in strength and mildness in different persons. Some people may revisit certain stages for long time. Our health worker should think about emotional status of relative of dead child/person and respect the same for successfully getting the consent for MITS specimen collection,

Objectives

• Describe the standard operation procedure of 'Grief counseling' of caretakers of dead child before sample collection by MITS according to the study protocol.

2. Scope

This document is relevant to all health workers, involved in Grief Counseling, before collecting specimen by MITS. Grief counseling of parents and caretakers of the dead child needs to be done.
In analysis of grieving behaviors, context matters as much as the symptoms themselves. So, the therapists should keep cultural influences in mind.

3. Roles and Responsibilities

Role	Responsibilities
VHW and counselors in hospitals.	VHW offers friendly talk, in local language, emotional support, discussion and sharing knowledge about ways to tackle the situation. It should be natural, to the point, with good faith and caring
BCC supervisor	* BCC supervisor, along with VHW offers friendly talk, in local language, needful advice, emotional support, discussion and sharing knowledge about ways to tackle the situation. It should be natural, to the point, showing right path to relatives, with good faith and caring<br><br>*Executes and ensures that consent is given by parents/ caretakers for MITS collection.
MIT specialist	MITS done after Grief counseling and obtaining consent

4. Related Documents. To be completed by study staff, if applicable.

e. g. Consent form.

5. Useful Terms and Definitions

Review table and add additional project specific terms, as needed

VHW	Village Health worker
BCC supervisor	Behavior Change Communication Supervisor
MITS	Minimal invasive tissue sampling

6. Equipment

Consent form for signature of parents/ caretakers.

7. Required Precondition

Create friendly, emotionally supportive ambience so that consent for MITS is obtained from parents/ caregivers.

8. Procedure.

Bereavement is a type of grief involving the death of a loved one. Grief is a reaction to death of child that encompasses a range of feelings from deep sadness to anger, and the variable process of adapting to this significant loss. It is difficult for the relatives to accept the death of child and the event causes psychological shock. When a person experiences such event, stress hormones are secreted and these increased levels reflect on parent's behavior and reactions. The impact of death may cause disturbed

family relationship or the interaction with the society. It may affect physical, psychological, behavioral changes in caretakers.

After death of a child, the relatives have different thoughts, reflecting in their reactions as they make sense of their loss. There are following 5 stages of reaction from family. The emotions can be different in strength and mildness in different persons. Some people may revisit certain stages for long time.

1. Denial /Rejection. - When a loving child dies in house, the relatives cannot accept the situation immediately, and are in mindset of denial of the reality that death has occurred and they reject the idea that this could really be the truth. Research suggests people in death-denying cultures tend to have more anxiety around death than people in death-accepting cultures like tribes of Melghat.
2. Anger - When they realize the child is dead, they become very angry, thinking, how can this happen to their child?
3. Bargaining or blaming person or situation - When the anger subsides after realizing that the child is dead, the relatives try to blame somebody or some situation as the cause of death of the child.
4. Depression and guilt feeling - Relatives may think that they are responsible for the death of child and it is due to some mistake on their part. This guilt feeling can cause lot of psychological depression. After this stage, the relatives become dejected and feel very sad. It is followed by depression and feeling of gloominess.
5. Acceptance of the situation - At last they accept the reality of death of the child.

Our health worker should think about emotional status of relative of dead child/person and respect the same.

Counselling specifically for MITS:

After receiving the news of death of a child in community, the VHW and BCC supervisor will visit the house, or counselor in the hospital. They should start the conversation preferably in local Korku language with parents and relatives. It is important to inform it to parents as …. 'we don't know the exact cause of deaths in most of the under 5 children deaths in Melghat. It is important to know the cause of death so that we will be able to prevent deaths due to similar cause as specific treatment can be given to other children preventing their deaths. So, the knowledge of the disease and the cause of death of children is important for society in general.

In culture of Melghat, helping each other in all possible ways is way of life which is being followed in the whole society. By doing MITS, we may be able to prevent deaths of other children which may be the biggest help for the children in community.

Some people may be talking against MITS but saving lives of poor people is like serving the god. So, the negative feeling should be neglected.

Some parents may be against the decision of MITS, they should be explained very patiently in their mother tongue by person of same community in details about MITS. If any person in family is against MITS, he/she should be counseled and explained about

MITS by putting extra efforts. This family member may convince all remaining family members.

It is specifically explained that by doing MITS the body will not be disfigured. There will not be any bleeding. Needle prick marks will be seen over head and chest. The examination of tissues removed from babies' body will be done by expert doctors recognized by government. And for this examination, permission from government (HMSC, Indian government and Maharashtra government) is already been obtained. The report of the baby will be communicated to the relative.

Here is the opportunity to change the situation by doing this study for improving lives of children. This is the new technique; we should learn to accept and utilize it. And by doing this, you can support other children's healthy lives. Loss of your baby is a very big trauma and we fully sympathize but you will have to get over the grief, be courageous, fight with your dejection and depression and while moving on with life, utilize the opportunity of improving community children health.

We will do grief counseling to convince families to give consent for MITS after death of their children.

Techniques used in Grief Counselling[1]

Three of the biggest things a good grief counselor should do are to:

1. Let them talk about the deceased; ask them about the person, and allow them to speak about their lost loved one in a safe space.

2. Distinguish grief from trauma; if the parents are struggling to get an image out of their head or experiencing flash backs to the moment they learned of their loved one's death, they are experiencing trauma, which can keep them from working through their grief.

3. Deal with any guilt they are feeling and help them organize the grief; the client may feel guilty about what they did or didn't do while their loved one was alive, or they may feel guilty about not feeling "sad enough" or moving on while their loved one is dead. Encourage them to let go of the guilt and commit to living a life that will honor the deceased, even if that means forgetting about them for a little while.[2]

---

[1] Ref: Grief Counseling Therapy Techniques & Interventionshttps://positivepsychology.com/grief-counseling/ 5/23)

Ref : Grief Counselling Therapy Techniques & Interventions https://positivepsychology.com/grief-counseling/ 6/23

[2] Tyrrell, n.d.

While counselling a person in shock the points to be remembered by VHW/counsellors are as follows:

1) VHWs and counsellors should act as a companion and helper for the relatives.
Companioning is about…
Honoring the spirit, not focusing on the intellect.
Curiosity. Learning from others, not teaching them.
Bearing witness to the struggles of others, not directing them.
2) Be empathetic. Listen with the heart, not analyzing with the head. Listen in a supportive manner to the parents' concerns.
3) Some people take time to process their emotions before reaching out, and others may be in shock or denial for a period of time. Use the different approach of grief counseling as per nature of people.
4) Use minimum words.
5) Body language should be friendly with friendly touch. Walking alongside, not leading. Be still, not frantic movement forward.
6) Make the relative to talk you about the death. Ask him or her about any funeral or memorial services.
7) Begin the talk with distant relative first and then come to near relative of the child e.g. Parents.
8) Express your grief.
9) Respecting disorder and confusion, not imposing order and logic.
10) Share the feeling that it is difficult to face the situation. Counsellor should witness relative's pain.
11) Make / stimulate the relative to talk.
12) Listen quietly.
13) Tell them, crying is necessary as it relieves the negative emotions.
14) Explain that the challenge is to adjust to the new reality of life in the absence of the loved one, they have to be courageous and have to move on.
15) Tell them that they also need help from society.
16) A community can provide emotional and financial aid when people are vulnerable.
17) Assist individuals to draw on their own strengths and develop healthy coping mechanisms that permit them to gradually resume their pre-disaster (or pre-loss) level of functioning.

Smaller, everyday rituals can also be helpful for those grieving a loss; these are called Rituals to Commemorate, and include things like lighting a candle/lamp and thinking of the loved one, or visiting the funeral process and burial site, offer them small help if needed and leaving a tribute or symbolic item, (we will think for Melghat).

Our VHWs or counselor may arrange food for near relatives (parents, siblings and grandparents) for one or two days. This will help VHWS and counsellors' better access to the affected family and person. Also, will take care of nutrition and other care of the person.

Outcome:

Getting the consent form for MITS collection signed by parents/ caretakers.

Appendix:

- Training schedule of health workers by Psychologist Dr Shubhada Khirwadkar, Dr Deoghare and Dr. Abhijit.
-

1. Tikmani SS, Saleem S, Moore JL, Reza S, Gowder G, Dhaded S, et al. Factors Associated With Parental Acceptance of Minimally Invasive Tissue Sampling to Identify the Causes of Stillbirth and Neonatal Death. Clin Infect Dis. 2021;73(Suppl_5):S422-s9.
2. Das MK, Arora NK, Kaur G, Malik P, Kumari M, Joshi S, et al. Perceptions of family, community and religious leaders and acceptability for minimal invasive tissue sampling to identify the cause of death in under-five deaths and stillbirths in North India: a qualitative study. Reproductive health. 2021;18(1):168.
3. Das MK, Arora NK, Debata P, Chellani H, Rasaily R, Gaikwad H, et al. Why parents agree or disagree for minimally invasive tissue sampling (MITS) to identify causes of death in under-five children and stillbirth in North India: a qualitative study. BMC pediatrics. 2021;21(1):513.
4. Kongsholm NCH, Lassen J, Sandøe P. "I didn't have anything to decide, I wanted to help my kids"-An interview-based study of consent procedures for sampling human biological material for genetic research in rural Pakistan. AJOB Empir Bioeth. 2018;9(3):113-27.
5. Lawrence S, Namusanya D, Mohamed SB, Hamuza A, Huwa C, Chasweka D, et al. Primary motivations for and experiences with paediatric minimally invasive tissue sampling (MITS) participation in Malawi: a qualitative study. BMJ open. 2022;12(6):e060061.

-
