# Supplementary material for: Community—Minimal Invasive Tissue Sampling (cMITS) using a modified ambulance for ascertaining the cause of death: A novel approach piloted in a remote inaccessible rural area in India
Source: Arch Public Health. 2023 Apr 27;81:72. doi: 10.1186/s13690-023-01062-x (PMC10134564; doi:10.1186/s13690-023-01062-x)
Supplement: Supplementary file 2 — Additional file 2: Annexure 2: MITS Ambulance sterilisation SOP. [file 13690_2023_1062_MOESM2_ESM.pdf]

# MITS Ambulance sterilisation

(1st May 2020 to 30th April 2021) (Dharni Block of Amaravati district)

- Roof, floor and 3 walls of MITS ambulance are made air tight.
- Roof, floor, all walls and door are made smooth to prevent accumulation of dust.
- The MITS ambulance is cleaned and washed thoroughly from inside and outside.
- There is regular cleaning of all inner surfaces of ambulance with Bacillol 25 with spray, at least once a week and after MITS.
- MITS ambulance is always ready by fogging with Glutaral (15.2gm) and Dimethanol (19.7gm) (after MITS and before MITS) to maintain asepsis .
- Fogging with H2O2 , Silver nitrate for one hour is done during emergency or Air sterilisers are used for instant sterilisation before MITS procedure.
- Air conditioner is installed in MITS ambulance.
