# Supplementary material for: Community—Minimal Invasive Tissue Sampling (cMITS) using a modified ambulance for ascertaining the cause of death: A novel approach piloted in a remote inaccessible rural area in India
Source: Arch Public Health. 2023 Apr 27;81:72. doi: 10.1186/s13690-023-01062-x (PMC10134564; doi:10.1186/s13690-023-01062-x)
Supplement: Supplementary file 3 — Additional file 3: Annexure 3: MITS Ambulance Set up. [file 13690_2023_1062_MOESM3_ESM.pdf]

MITS Ambulance Set-up (1st May 2020 to 30th April 2021) (Dharni Block of Amaravati district)

i. There is adequate ventilation, air purification and light system in the MITS ambulance.
ii. There is clearly delineated space e.g., drawers for storage of clean items including PPE, linens and MITS Kits in upper inner compartments of MITS table.
iii. There is clean and climate-controlled, temperature regulated space for storing MITS samples (microbiology and histopathology) in upper and inner compartments of MITS table, properly separated from storage of clean and dirty materials by partition.
iv. The PCR samples are stored in cryovials in 5L mini liquid nitrogen container (temperature <-80^{0}C to -160^{0}C) in inner part of MITS ambulance outside the MITS table.
v. Designated space for donning and doffing of PPE at the entry in MITS ambulance.
vi. There is clearly delineated space for storage of 'dirty' materials in MITS table, below the wash basin, in outer most lower compartments of MITS table. It is separated from clean storage by partition.
vii. Clearly delineated space for biohazard waste in outermost compartment below wash basin in MITS table.
viii. Adequate handwashing, wash basin with tap and waste disposal receptacles appropriately placed and marked in outer most part of MITS table.
