# Supplementary material for: Community—Minimal Invasive Tissue Sampling (cMITS) using a modified ambulance for ascertaining the cause of death: A novel approach piloted in a remote inaccessible rural area in India
Source: Arch Public Health. 2023 Apr 27;81:72. doi: 10.1186/s13690-023-01062-x (PMC10134564; doi:10.1186/s13690-023-01062-x)
Supplement: Supplementary file 4 — Additional file 4: Annexure 4: Minimally Invasive Tissue Sampling SOP MAHAN. [file 13690_2023_1062_MOESM4_ESM.pdf]

Minimally Invasive Tissue Sampling (MITS) Standard Operating Procedure (SOP)

(By MAHAN trust)

(9.2.20)

(1st May 2020 to 30th April 2021) (Dharni Block of Amaravati district)

Table of Contents

1. Purpose 4

2. Scope 4

3. Roles and Responsibilities 4

4. Related Documents 5

5. Useful Terms and Definitions 5

6. Equipment / Reagents 5

6.1. MITS Sample Collection Kit with the Specimen Kit ID number 5

6.2. MITS backup box 5

6.3. Material to be obtained by participating site 5

6.4. Reagents to be obtained by participating site 6

7. Safety 6

8. Procedure 6

8.1. Preparation of the body and materials 6

8.2. Anthropometric Measurements 7

8.3. Body Inspection 8

8.4. Body Palpation 9

8.5. Photography 9

8.6. Body Cleaning and Sterilization 9

9. Specimen Collection 11

9.1. Cerebrospinal Fluid (CSF) Occipital Approach for Microbiology 11

9.2. Brain/CNS MITS for Microbiology (Occipital Approach Only) 13

Posterior/Occipital approach - All Cases 13

9.3. BRAIN/CNS MITS for Histology (Occipital and Fontanelle Approaches) 14

Posterior/Occipital approach - All Cases 15

Fontanelle approach – Stillbirth, Neonates, and Infants with OPEN fontanelle Only 15

9.4. Nasopharyngeal (NP) Swab Sample 16

9.5. BRAIN/CNS MITS for Histology Trans-nasal approach – Infant with closed fontanelle, Child, Adult Cases ..... **Error! Bookmark not defined.**

9.6. Blood Collection 20

9.7. Lung/Thorax MITS for Microbiology 24

9.8. Lung/Thorax MITS for Histology 26

9.9. Liver MITS for Microbiology ..... **Error! Bookmark not defined.**

9.10. Liver MITS for Histology ..... **Error! Bookmark not defined.**

9.11. Rectal Stool Sample ..... **Error! Bookmark not defined.**

9.12. Skin Sample ..... **Error! Bookmark not defined.**

9.13. Placenta and fetal membranes and cord for microbiology 28
10. End of the procedure and completion of the MITS Specimen Collection Form 29
10.1. MITS Specimen Collection Form 29
10.2. Excessive seepage or bleeding though the biopsy entry points 29
10.3. Containers and Tools 31
10.4. MITS Envelope 31
11. References 32
12. Appendices 32
12.1. Appendix A: MITS Kit Components 32
12.2. Appendix B: Back-up box Components 34
12.3. Appendix C: Table for formalin jars and cryogenic vials 35

1. Purpose

The purpose of this Standard Operating Procedure (SOP) is to describe the procedures for specimen collection during the MITS procedure.

Objectives

- Describe the standard operation procedure of the MITS according to the study protocol
- List the materials and reagents not included in the MITS sample collection kit
- Articulate the steps to adequately prepare and disinfect the body for the MITS
- Identify the components of external inspection, the anthropometric measurements and the pictures.
- Obtain MITS Samples for microbiology and pathology
- Identify the presence of ascites fluid and pleural effusions and how to obtain samples for analysis
- Describe the basis of the examination and sampling of the placenta
- State the steps to be done at the end of the procedure

2. Scope

This document is relevant to all tissue and non-tissue specimens collected from the following populations. Age-specific case instructions will be noted in the text.

- **Perinatal**
    - **Stillbirth -** baby born with no signs of life at or after 28 weeks' gestation
    - **Neonatal** - live born babies who die before 28 days of age
- **Infant(post neonatal)** – baby who dies between 29 days and 1 year of age
    - **Infant, open fontanelle**
    - **Infant, closed fontanelle**
- **Child -** bodies of children > 1 year of age – 5 years of age
- **Adult** – bodies > 19 years of age

We used sample collection SOP for those deaths aged 16-19 as per MITS SOP of adults>19 years of age.

3. Roles and Responsibilities

The table below provides teams with an understanding of the individuals involved with the MITS SOP

Role	Responsibilities
**MITS Specialist**	Executes and ensures specimens are collected and assures compliance with the procedure. Specially trained to perform MITS. Referred to as “specialist” throughout the SOP.
**MITS Assistant**	Assists the MITS specialist during the MITS procedure. Manages the specimen collection containers and the MITS Specimen Collection Form. Moves the body and to helps maintain the adequate positions of the body. Referred to as the “assistant” throughout the SOP.

4. Related Documents

To be completed by study staff, if applicable

Title	Version Number

5. Useful Terms and Definitions

Review table and add additional project specific terms, as needed

**CNS**	Central Nervous System
**CSF**	Cerebrospinal fluid
**ID**	Identification number
**MITS**	Minimally Invasive Tissue Sampling
**PPE**	Personal protective equipment
**SMC**	Severely malnourished children under the age of 5 years.
Filter Paper	*Blood Spot Card*

6. Equipment / Reagents

Check list /Requirements for Molecular Pathology Lab:

ICE CONTAINER	BLOOD CULTURE BOTTLE
CHILLER FOR CRYOVIALS	STERILE JAR FOR CSF
CRYOVIAL STAND	2 ML TUBES FOR M/B LUNG CULTURE
TUBE RACK	MARKER
MITS KIT	SCISSORS
MITS FORM	EXTRA EDTA TUBES FOR M/B MALARIA TESTING
STERILE JAR FOR PLACENTA	EXTRA STERILE TUBES FOR SAMPLE COLLECTION

6.1. MITS Sample Collection Kit with the Specimen Kit ID number

See Appendix A for all components

6.2. MITS backup box

See Appendix B for all components

6.3. Material to be obtained by MAHAN

Materials tp
Disposable scrub, lab coat, gloves, boots, shoe covers, hat
Paper towels
Safety goggles
Forceps
Gauze rolls 36' x 100 yards, and 4x4
Cotton swabs for cleaning the body with reagents

Camera for pre-procedure pictures (high resolution, >300 ppi preferred)<br>Body weight scale
• for stillbirths and neonates (0-5 Kg) calibrated in grams
• for children (5-25 Kg) calibrated in grams
• for adults (large scale range) calibrated in grams
Rolling cart or auxiliary table
MITS cool box and cool packs to maintain temperature control of samples for microbiological analysis (Liquid nitrogen and Dry ice will be preferred in our project)
MITS cryogenic vial rack
MITS test tube rack
MITS tray on which to place formalin jars
Biowaste container

6.4. Reagents to be obtained by MAHAN

Alcohol 70%

- Conservation at room temperature | Long stability |Inflammable

Iodine solution

- Conservation at room temperature | Long stability

Any reagent required for microbiological sample collection, processing, and analysis, if specified by the project protocol

- e.g. RNA *later* or other stabilization solution will not be purchased in our project as it is very costly, and we do not have funding.

Hemostatic agent :

We will use Tincture Benzene to stop bleeding after biopsy.

Monsel's solution (we will not use it as it is very costly)–
• Ferric subsulfate 20%, used to stop bleeding after biopsy (we will not use it as it is very costly)
• Conservation at room temperature in an airtight container protected from light.
• Crystallization may occur at temperatures below 22 degrees. Warming the solution may dissolve the crystals.

Bleach 10%

- Or project-specific standard for cleaning surfaces

Project-specific supplies for blood culture, if applicable

7. Safety

Ensure appropriate personal protective equipment (PPE) is worn by all MITS personnel<br>Dispose of needles and all waste generated during procedure in appropriate container as per biosafety laboratory protocols (Biomedical waste disposable)

8. Procedure

8.1. Preparation of the body and materials

Prior to starting the MITS specimen collection

The Specialist and Assistant will wear the appropriate PPE to perform the MITS (not included in kit).
☐ gown ☐ cap ☐ mask ☐ gloves x2 ☐ goggles ☐ shoe covers/boots

The Assistant picks up the body from the home or ward and checks the surname (or name of mother for stillbirths and early neonates) in the autopsy request document.

The Assistant takes the body to the MITS ambulance or room and places it on the table in a supine position.

Assistant and specialist confirm MITS kit contains all items on the list of contents. Assistant arranges items from MITS kit on disposable placemat.
☐ Confirm MITS kit contains all items in the list of contents, arrange items, and apply labels to tubes as needed. Gather all materials and reagents not included in the MITS kit prior to starting the MITS specimen collection.

The Assistant and The Specialist check once again the name, surname, official information related to autopsy report (if available), and informed consent.
☐ Check name ☐ Informed consent

The Assistant prepares additional materials and equipment (not included in kit) required for MITS procedure.

Apply labels, as needed

The Assistant completes the MITS Specimen Collection form Overview Section

Site ID
MITS specialist
MITS assistant
Date of death
Time and date received
Circumstances preventing MITS
Time and date MITS initiated.
Placenta submitted

8.2. Anthropometric Measurements

Note that different scales are used for weight
•Scale for stillbirths and neonates (Perinatal)
•Scale for children
•Scale for adults
The Specialist places the body on the appropriate type of scale and measures the weight.

Note the body should be weighed alone, or the tare discounted if an adult or cart is used to hold the body.

The Assistant records the weight on the MITS Specimen Collection Form.

Weight______☐ g ☐kg

Grams – perinatal (stillbirth/neonates)
Kilograms – infant, child, adult The Specialist measures the basic anthropometric data using the tape measure (included in MITS kit).

The Assistant records on the MITS Specimen Collection Form

Height/length (all): The specialist places the body lying on side with legs extended and, using the flexible tape, measures the distance from vertex (top of head) to the heel of the right foot.

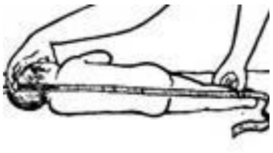

MUAC (6 month-5 years): Specialist locates the acromial process on the RIGHT shoulder and the olecranon process at the elbow. At the midpoint between these two points place the tape measure perpendicular to the long axis and measure and record the circumference.

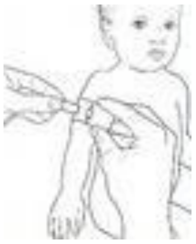

Head circumference (0 to 5 years): Specialist places the measure tape around the head so that the tape lies across the frontal bones of the skull, slightly above the eyebrows, perpendicular to the long axis of the face, above the ears, and over the occipital prominence at the back of the head.

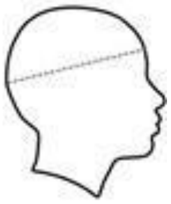

Lower leg length
(Stillbirth/Neonate only): Specialist measures the right leg from the medial malleolus to the medial condyle of the tibia.

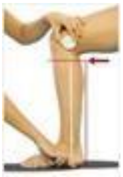

Foot length
(Stillbirth/Neonate only): Specialist measures the distance from the heel to the longest toe of the right foot parallel to the long axis of the foot.

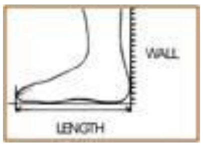

8.3. Body Inspection

Note: In perinatal cases, the body can be placed on top of the second disposable placemat

The Specialist performs an inspection of the external genitals. The Assistant records sex on the MITS Specimen Collection Form	Sex ☐Male ☐Female ☐Indeterminate
**Stillbirth only:** The specialist determines whether the body is fresh or macerated (skin and soft-tissue changes such as skin discoloration or darkening, redness, peeling and breakdown), and in case of maceration evaluates the grade. Assistant records on the MITS Specimen Collection Form (Note: this is recorded under Section 6 of the MITS Sample Collection Form)	☐ Fresh, Grade 0, Non-macerated<br>☐ Grade 1 (skin slippage and peeling, small patches; <8 hours)<br>☐ Grade 2 (extensive skin peeling, large patches; 2-7days)<br>☐ Grade 3 (mummification; >8days)
The Specialist performs a detailed external inspection of the whole body looking for visible congenital physical anomalies or	Evidence of Trauma<br>Gross Facial Abnormalities<br>Abdominal distention

malformations, evidence of trauma, external tumors, skin rashes and lesions or changes of the color of the skin (e.g. depigmentation, areas of darkness). The Assistant indicates presence or absence of findings on the MITS Specimen Collection Form.	Umbilical abnormal<br>Jaundice/Icterus<br>Petechiae<br>Rash<br>Other skin lesions<br>Bleeding<br>Other significant gross findings

8.4. Body Palpation

Note: NOT to be done on stillbirths

The Specialist performs a detailed palpation of the **abdomen** in order to detect hepatomegaly, other visceromegalies or abdominal masses. The Specialist performs a detailed palpation of the **superficial lymph node areas** (anterior and posterior cervical, supraclavicular, axillary and inguinal areas).

The Assistant indicates presence or absence of findings on the MITS Specimen Collection Form.

8.5. Photography

The Assistant places body on a non-patterned light or dark blue background alongside the ID photo card with scale provided with the kit.

The Assistant takes high resolution (>/=300 ppi, if possible) photographs and documents on MITS Specimen Collection Form.	☐ Front<br>☐ Back<br>☐ Side<br>☐ Nails<br>☐ Other gross lesions/Abnormalities<br>☐ No photos taken, explain why

The Specialist checks the quality of the photos for clarity and completeness. Assistant retakes, if needed.

8.6. Body Cleaning and Sterilization

Clean the areas of the body to be punctured before beginning the MITS procedure, as follows:	
The Specialist cleans body with water and then dries with gauze the areas of the body to be punctured. Areas vary by patient age and are indicated for each on the MITS Specimen Collection Form.	
**Specific areas to be cleaned are indicated below for each category of MITS patient.**	
**Stillbirth**	Cleaning completed:<br>☐ nuchal area ☐ anterior fontanelle<br>☐ thorax ☐ axillae ☐ abdomen

**Neonatal**	Cleaning completed:
**Infant, open fontanelle**	☐ nuchal area ☐ anterior fontanelle
	☐ nares ☐ thorax ☐ axillae
	☐ abdomen

**Infant, closed fontanelle**	Cleaning completed:
**Child**	☐ nuchal area
	☐ nares ☐ thorax ☐ axillae
	☐ abdomen

The Specialist cleans with abundant spirit/alcohol(70%) the **project-specific areas** of the body to be punctured. Cleaning is performed with circular movements from the center to the periphery.

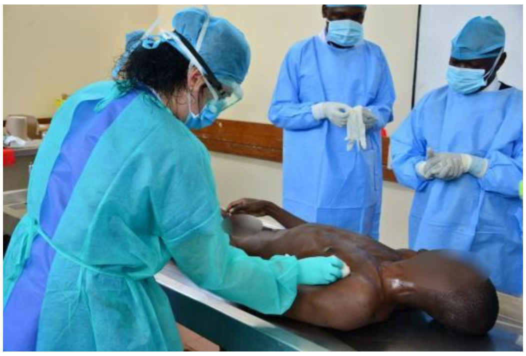

Allow alcohol to air dry for at least 5 minutes. The Specialist cleans with abundant iodine solution the **project-specific areas** of the body to be punctured. Cleaning is performed with circular movements from the center to the periphery.

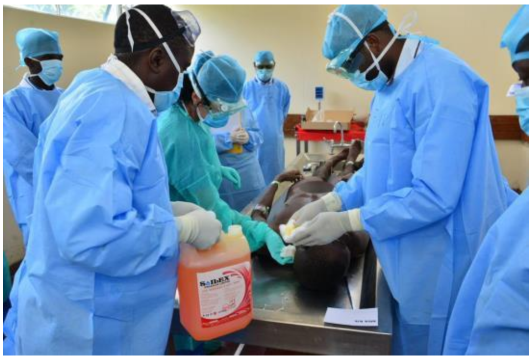

Allow iodine to air dry for at least 5 minutes.

THEN AGAIN CLEAN AGAIN WITH spirit/70% alcohol/ETHANOL.

Remove the soiled sheet and Shift the body on fresh sterile sheet. Personnel must change the gloves before starting the procedure

9. Specimen Collection

9.1. Cerebrospinal Fluid (CSF) Occipital Approach for Microbiology

**20G spinal puncture needle – Perinatal cases (Yellow)**	Kit Bag 2
**18G spinal puncture needle – Infants with closed fontanelle, child, adults (Pink) (L.P. needle may be used?)**	Kit Bag 2

**16G hypodermic needle (Purple)**	Kit Bag 2
**20 mL syringe**<br>*Note: Projects working with perinatal cases may prefer a smaller 10 mL syringe*	Kit Bag 2

**10 mL sterile tube (pre-labeled)**	Kit Bag 3

- *Note that the specific bag where materials will be located is project specific and the locations here are specific to the MITS Training Kits.*

a. The Specialist rotates and maintains the head in a lateral position, while the body is in a supine position

- The assistant helps to maintain the head in the correct position

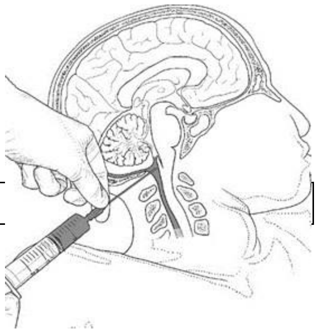

b. The Assistant prepares a new and sterile 20 G (neonates/infants) or 18 G (adults and children) spinal puncture needle, a 20 mL syringe, and a 10 mL empty sterile tube for CSF fluid included in the MITS kit.

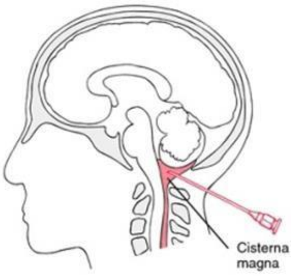

c. The Specialist selects and opens the spinal puncture needle (of the correct gauge for the type of case).

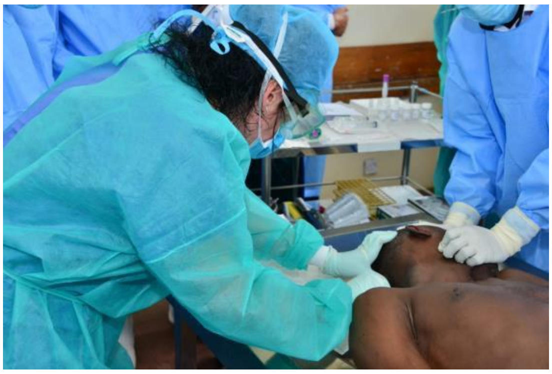

d. The Specialist stabilizes the spinal needle in the middle line below the occipital bone between the **squama** and the atlas bone

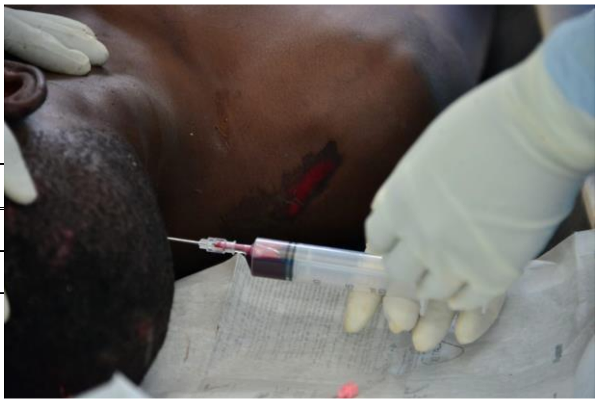

- Insert needle through the skin.

Advance the needle leaning it towards the orbital cavities.

The angle with the skin of the back should be approximately 75º. Advance the needle slowly but smoothly.

- Occasionally, a characteristic "pop" is felt when the needle penetrates the dura.

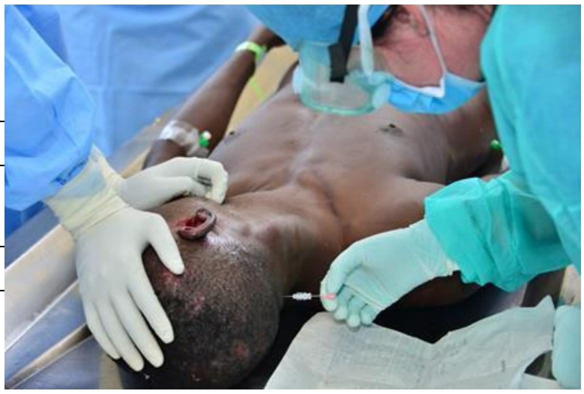

- Otherwise, the guide should be withdrawn after approximately 1 cm and observed for fluid return.
- If no fluid is obtained, replace the guide, advance or withdraw the needle a few millimeters, and recheck for fluid return
- Continue this process until drops of fluid are successfully obtained

e. The Assistant opens the 20 mL new sterile syringe package

f. The Specialist attaches the syringe to the needle and draws off CSF.

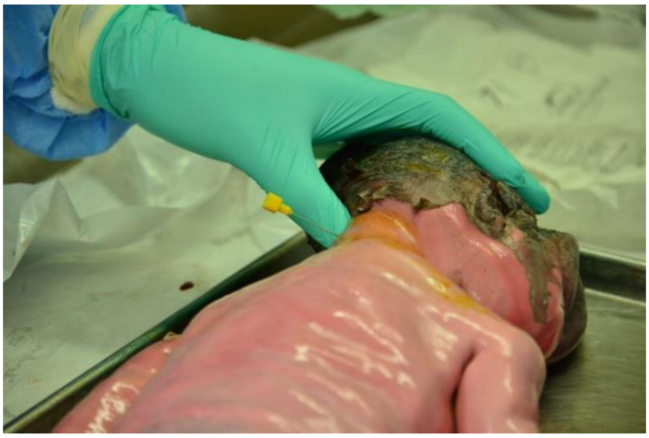

g. The Assistant opens the package of the 16G hypodermic needle (Purple).

h. Holding the syringe vertically with the needle up, the specialist removes the spinal needle from the Luer-Lok of the syringe and places it in the sharps bin. The specialist then attaches the 16G hypodermic needle to the Luer-Lok of the syringe.

g. The Assistant opens the 10 mL sterile tube and the specialist transfers the CSF from the syringe to the tube.

h. Assistant caps the tube.

i. The Assistant fills the required information in the MITS specimen collection form	CSF ☐ Y ☐ N<br>Total volume collected: _____(ml)<br>☐ Clear☐ Turbid ☐ Purulent☐ Hematic
j. The assistant stores the 10mL sterile tube in the MITS test tube rack	
Collect In 2 Green Capped Tube (Label with Extra Stickers)	

9.2. Brain/CNS MITS for Microbiology (Occipital Approach Only)

**Bard Monopty 16G 160mm (labeled "BRAIN")**	Kit Bag 1
**BRAIN/CNS cryogenic vial (pre-labeled)**	Kit Bag 3

Posterior/Occipital approach - All Cases

a. The Assistant selects the CNS cryogenic vial and the BARD 16G, 160mm needle labeled "BRAIN" from the MITS kit

b. The Specialist starts the occipital puncture, using an entry point 2 cm below the approach used for the CSF puncture.

- Midline 3-4 cm below the occipital bone

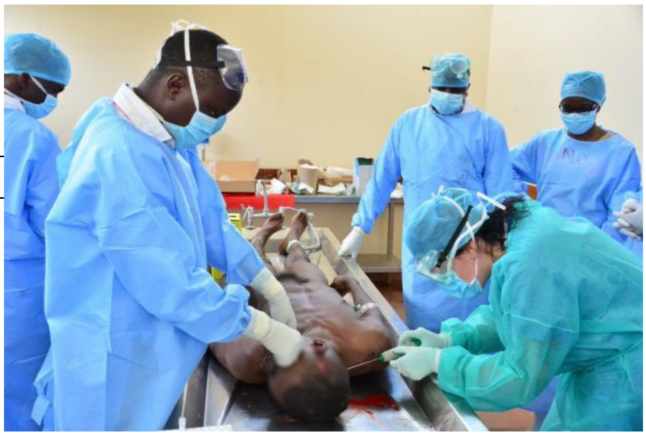

c. The Specialist advances the needle through the skin, with a 30◦ angle with the skin of the back, leaning it towards the orbital cavities, advancing the needle slowly but smoothly into the brain

- **Note: The needle should freely advance into the cranial cavity.**

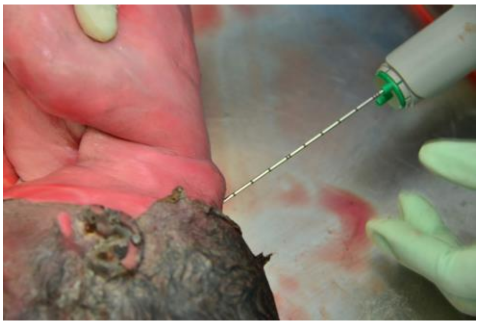

d. The Specialist obtains the biopsy

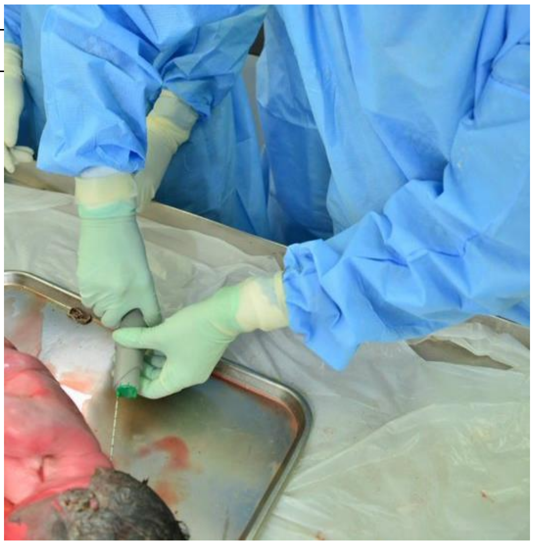

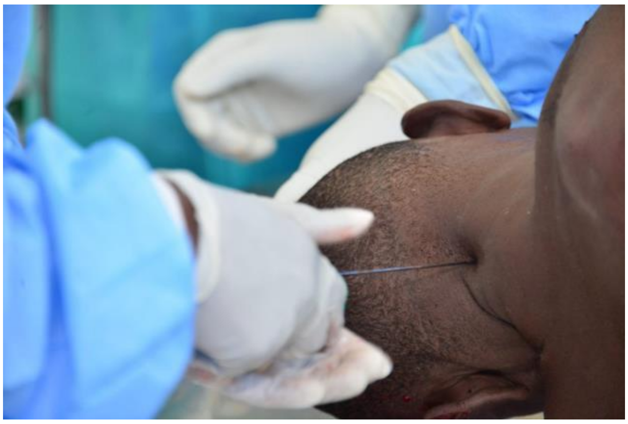

e. Withdraw the needle.

f. The Specialist removes the samples by rotating the needle handle clockwise and then depositing the sample into the BRAIN/CNS cryogenic vial.

g. The Specialist repeats steps b. through step g. Advancing the needle in different directions/depths to obtain samples from different brain regions. At least 3 samples are obtained / and stored in Cryovials Labeled CNS-P for molecular pathology.

h. The Assistant stores the cryogenic vial in the MITS collection rack or tray

CNS, posterior- cryogenic vial (3)
☐ Y ☐ N

i. The Assistant fills the required information on the MITS Specimen Collection form

9.3. BRAIN/CNS MITS for Histology (Occipital and Fontanelle Approaches)

Materials Needed	MITS KIT Location
**BRAIN/CNS formalin jar (pre-labeled)**	Kit Bag 4
**Same needle from Section 9.2 (BRAIN/CNS Microbiology)**	re-use from 9.2

Posterior/Occipital approach - All Cases

a. The Assistant selects the BRAIN/CNS formalin jar from the MITS kit

b. Using the same needle used for microbiology punctures:

The Specialist repeats the occipital sampling using the same entry points(Suboccipital region) and following the same procedure and puts 6/3 samples in the BRAIN/CNS, posterior formalin jar. (Container Labeled CNS-P (LOC)

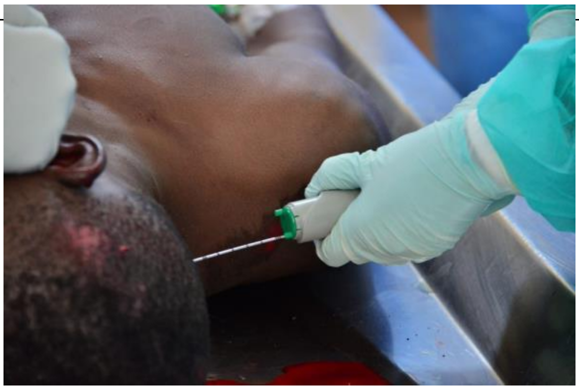

c. The Assistant stores the BRAIN/CNS, POSTERIOR formalin jar in the MITS tray.

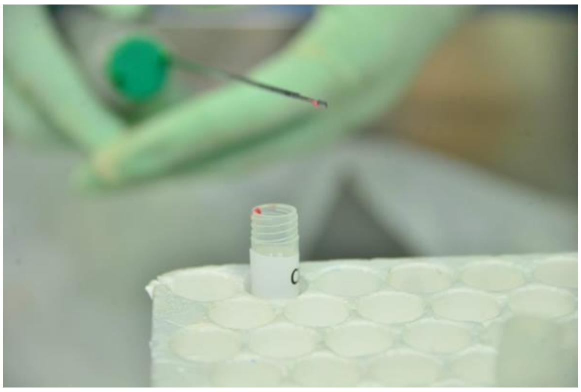

d. The Assistant fills the required information on the MITS Specimen Collection form	CNS, posterior- formalin (6)<br>☐ Y ☐ N

Fontanelle approach – Stillbirth, Neonates, and Infants with OPEN fontanelle Only

MITS Specimen Collection

a. The Specialist locates by palpation the anterior fontanelle and checks whether it is open

- **The anterior fontanelle remains opened the first 12-18 months of life.**
- **In older infants and in children, the Anterior fontanelle is already closed and cannot be punctured.**

The approximate location of the fontanelle is shown in the figures.

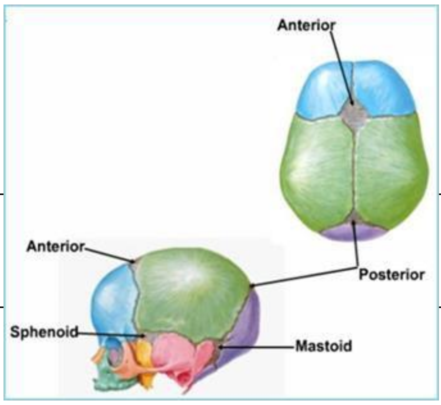

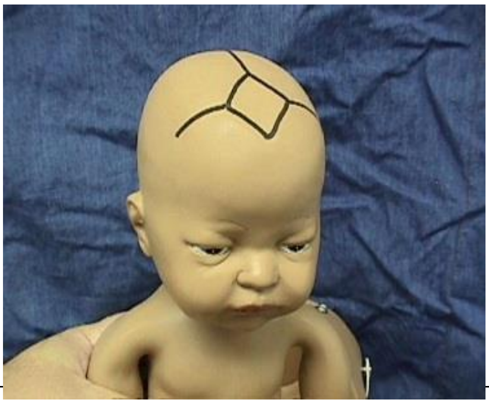

b. Using the same needle that was used for the occipital puncture.
The Specialist obtains 6 samples by puncturing the anterior fontanelle and puts them into the same BRAIN/CNS, formalin jar. The Specialist aims the needle in different directions/depths to obtain samples from different regions of the brain.

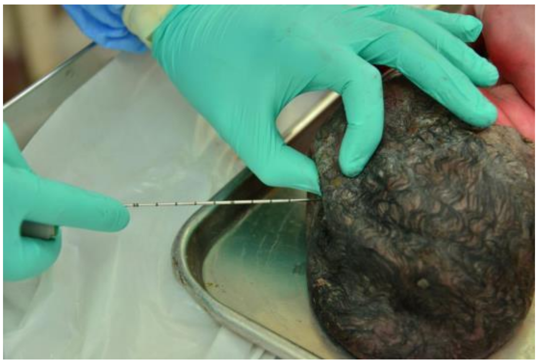

For molecular Pathology work

In case of stillborn/neonates or infants (before closure of ant. Fontanelle) only –
collect 3 samples/cores from fontanelle region in cryovials labeled CNS-P
Repeat Above Steps For CEN Tube

For histopathlogy :

In Case Of Stillborn/Neonates Only –
Collect 3 Cores From Fontanelle Region In Formalin/Cryovials Labeled CNS-P(LOC)Repeat Above Steps ForCEN Jar

c. The Assistant fills the required information on the MITS Specimen Collection form	CNS, fontanelle – formalin (6)<br>☐ Y ☐ N

9.4. Nasopharyngeal (NP) Swab Sample

10. NASO-PHARYNGEAL SWABS:

Clean Nasal Area with Sterile Cloth.

**Nasopharyngeal (NP) swab tube with 1 mL viral transport media (individually wrapped, NOT pre-labeled)**	Kit Bag 1
**NP label**	Kit Envelope

**MITS Specimen Collection**
**NOTE: This procedure is NOT to be done on Stillbirths.**

NOTE: This sample should be collected BEFORE Brain/CNS sampling through Trans-nasal approach.

a. The Assistant takes the NP swab tube 12 X 80mm with 1 mL viral transport media from the MITS kit and labels it.

b. The Assistant opens the NP swab tube.

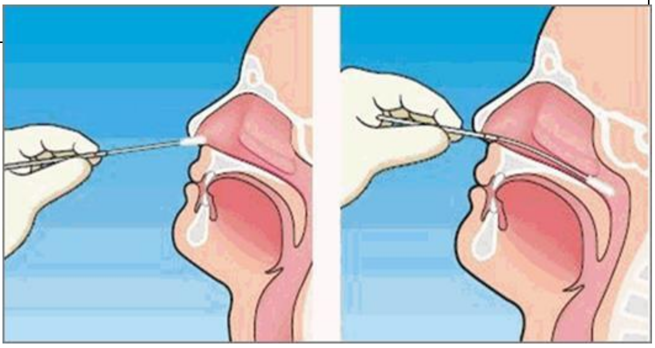

c. The Specialist tilts the head back 70◦ degrees and inserts the NP cotton swab into the nostril (until resistance is met at turbinates).

- Note: the swab should reach a depth equal to the distance from nostrils to outer opening of the ear.

d. The Specialist leaves the swab in place for several seconds to absorb secretions.

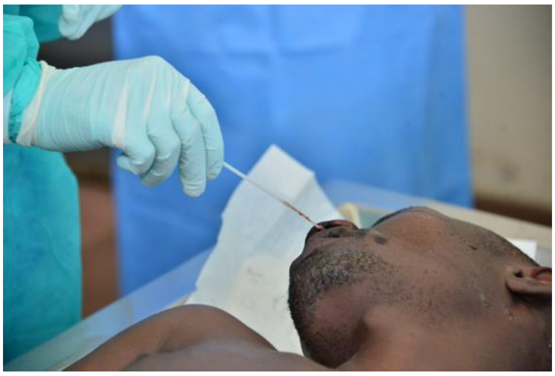

e. The Specialist slowly removes swab while rotating it.

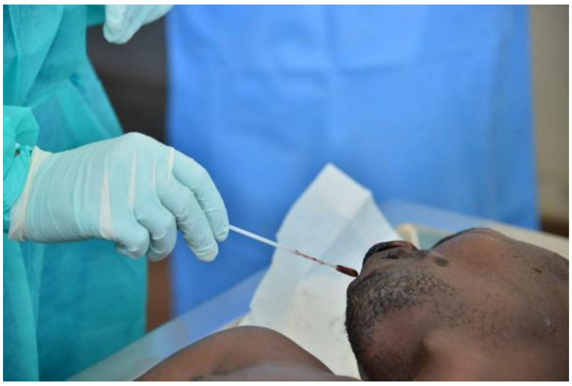

f. The Specialist swabs the other nostril with same swab and procedure.

g. The Specialist places the swab into NP tube with viral transport media

Note: The swab should be deep enough that the medium covers the cotton tip portion.

h. The Specialist breaks off the top portion of the stick and discards it.

i. The Assistant caps the tube and fills the required information on the MITS Specimen Collection form	NP swab<br>☐ Y ☐ N

j. The assistant places the NP tube with viral transport media in the MITS test tube rack. Label tube using stickers.

10.1. BRAIN/CNS MITS for Histology Trans-nasal approach – Infant, Child, Cases above age of 12-18 months with closed fontanelle), Adult

Material Needed	MITS Kits location
**Bone marrow biopsy kit**	Backup Box unbagged
**BRAIN/CNS formalin jar (pre-labeled)**	Kit Bag 4
**Same needle from Section 9.2 (BRAIN/CNS Microbiology)**	re-use from 9.2

MITS Specimen Collection

- **Trans-nasal brain specimens should NOT be collected for microbiology testing, due to Contamination with nasal flora.**
- **To reach the brain parenchyma, the cribriform plate must be perforated. Bone marrow trephine kit is used for this.**

a. The Assistant takes the bone puncture trephine and opens the container of a bone marrow biopsy needle.

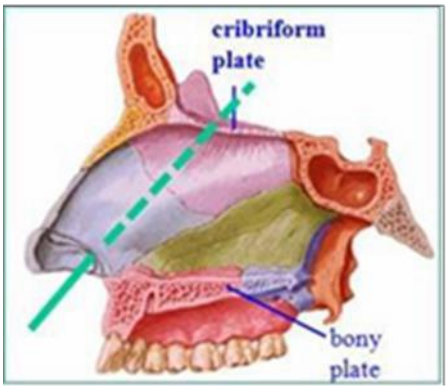

b. The Specialist inserts the stylet into trephine.

c. The Specialist introduces the needle with the internal guide into the nasal cavity with and angle of 45º, until reaching the roof of the nasal cavity (cribriform plate of the ethmoid bone).

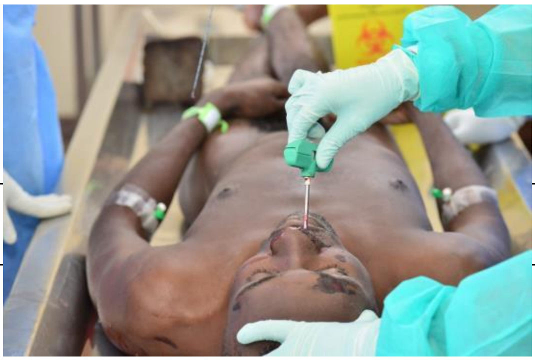

- The Assistant helps to maintain the head in the Correct position, when required.

d. The Specialist performs a constant pressure as well as a rotation back and forth to penetrates into the cranial cavity. Forceful tapping of the trephine may be required to penetrate the ossified bone.

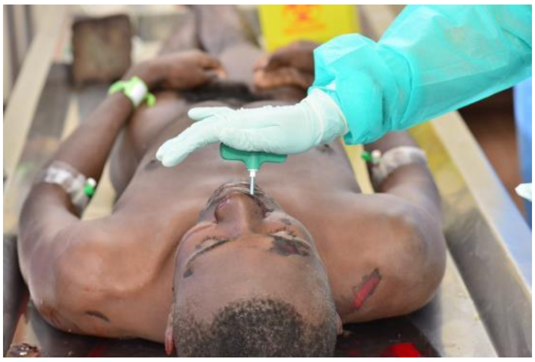

e. Specialist removes the trephine and stylet.
f. The Specialist introduces the CNS biopsy needle into the nasal cavity at an angle of 45º, until reaching the roof of the nasal cavity

g. Once the roof of the nasal cavity has been reached, the Specialist moves the needle around until the hole created with the trephine is identified

h. Once the hole is identified, the Specialist advances the needle into the cranial cavity and presses the button to obtain the sample.

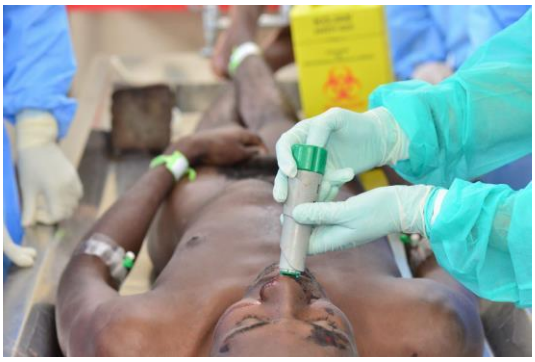

i. The Specialist withdraws the needle and deposits the sample into the CNS, TRANSNASAL formalin jar.

j. Specialist repeats steps f through i, advancing the needle in different directions/depths to obtain specimens from different regions of the brain. At least 6 specimens should be obtained.

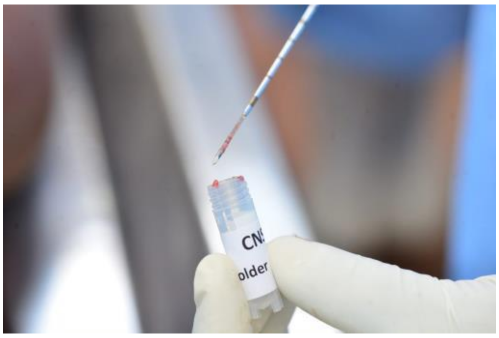

k. The Assistant stores the BRAIN/CNS formalin jar.

For Mol. Path. Work

Clean Nasal Area with Sterile Cloth. Pierce needle through cribriform plate and insert into brain tissue and collect 3 cores/samples from brain tissue.

Collect 3 samples/cores From Trans-Nasal Region In Cryovials Labeled CNS-T Repeat Above Steps For CEN Tube

FOR HISTOPATHOLOGY WORK (COLLECTED IN FORMALIN JAR)

Collect 3 Cores From Trans-Nasal Region In formalin Container Labeled CNS- T(LOC)

Repeat Above Steps ForCEN Jar

l. The Assistant fills the required information on the MITS Specimen Collection form	CNS, transnasal – formalin (6)
	☐ Y ☐ N

10.2. Blood Collection

First try right Subclavian artery
If no yield, try left side
If no yield, try heart

**20G spinal puncture needle – Perinatal (Stillbirth/Neonate) cases (Yellow)**	Kit Bag 2

**18G spinal puncture needle – Infant, child, adult cases (Pink)**	Kit Bag 2
**16G hypodermic needle (Purple)**	Kit Bag 2
**20 mL syringe**	Kit Bag 2
**EDTA vacutainer (pre-labeled)**	Kit Bag 3
**Blood spot card (pre-labeled)**	Kit envelope
**10 mL sterile tube (pre-labeled)**	Kit Bag 3

Note: Projects working with perinatal cases may prefer a smaller 10 mL syringe

a. The Assistant prepares a new and sterile needle (of the correct gauge for the type of case), a new 20 mL syringe, the EDTA vacutainer, a 10mL sterile tube and the blood spot card from the MITS kit box.

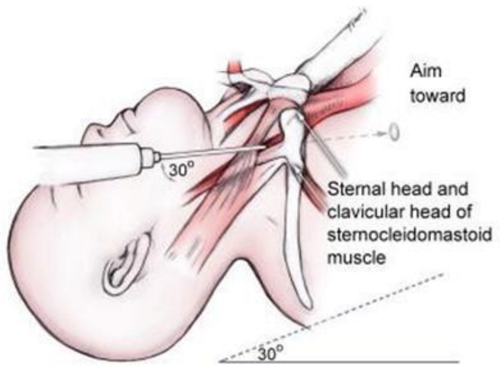

b. The Assistant opens the plastic wrap of a new sterile 20 mL syringe and a new 20G (Yellow)/18G (Pink) spinal puncture needle and gives them to the Specialist.

Note: The guide of the needle should be withdrawn, and needle connected to the syringe prior to puncture

c. The Specialist locates the midpoint of the clavicle (mid-way between the sternal notch and acromioclavicular joint) and inserts the needle 1 cm lateral and superior or inferior to the clavicle.

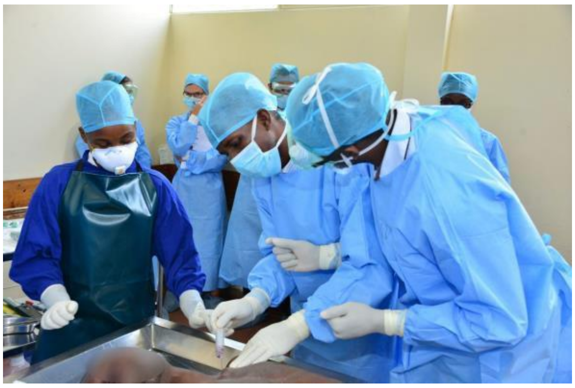

d. For supraclavicular approach, use an angle of 30º above the clavicle and aim inferior, the specialist attempts to first aim for the clavicle. Once the clavicle is passed, pull the plunger to make negative pressure.

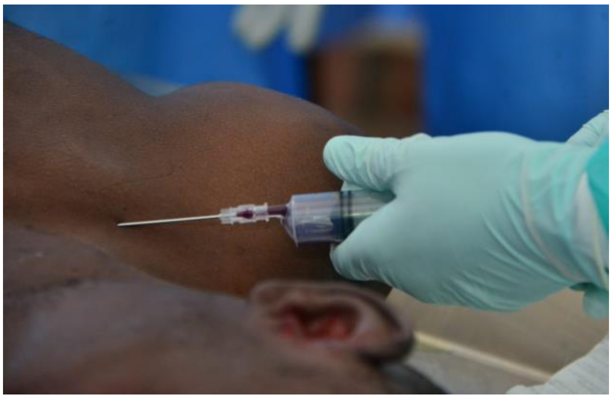

e. Maintaining the negative pressure with the plunger, the specialist continues advancing the needle in a plane almost parallel to the skin approximately 2-3 cm. Change the angle of penetration of the needle until venous blood is freely aspirated into the syringe.

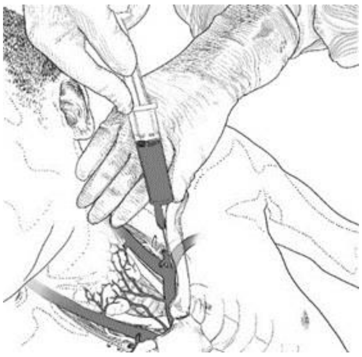

- **Note: In case no blood is obtained with supraclavicular approach, use an infra-clavicular puncture aiming to reach the subclavian behind the clavicle**

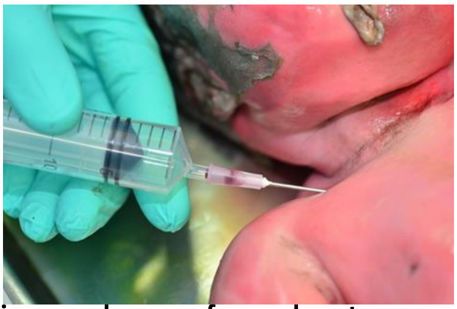

In the case that no or very little blood is obtained following this procedure, perform a heart puncture, using the same needle, aiming to obtain an adequate sample

e. The Specialist palpates the ribs to identify the left central, fifth intercostal space, and punctures there in a parasternal location.

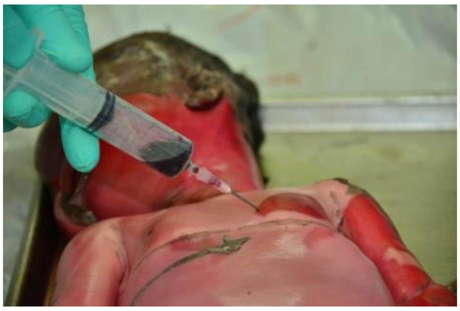

f. The needle should penetrate approximately 2-4 cm (children) or 5-6 cm (adults) in a sagittal direction.

g. Aspirate and redirect the needle as needed until obtaining blood.

h. Fill the syringe with as much blood as possible.

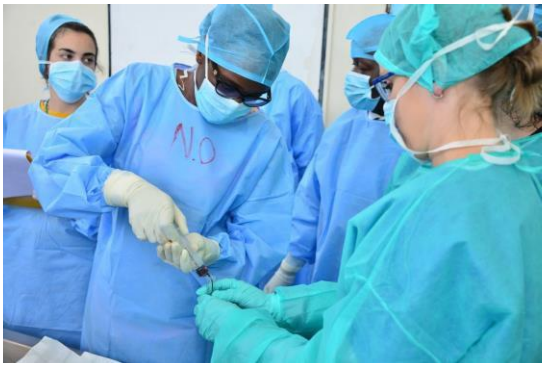

In case serum is obtained instead of blood, try again. In the case that no blood is obtained the second time, put the serum in the EDTA container only.
Note: Projects may have their own sample prioritization and should be addressed accordingly

g. The Assistant opens the package of the 16G hypodermic needle (purple).

h. Holding the syringe vertically with the needle up, the specialist removes the spinal needle from the Luer-Lok of the syringe and places it in the sharps bin. The specialist then attaches the 16G hypodermic needle to the Luer-Lok of the syringe.

i. The Assistant opens the EDTA tube and the specialist transfers up to 9 ml blood from the syringe to the tube. (for molecular pathology work).

j. The Assistant retrieves the blood spot card and the specialist deposits 4 large drops of blood onto each circle of the blood spot card, filling each circle. (Prepare DBS Cards)

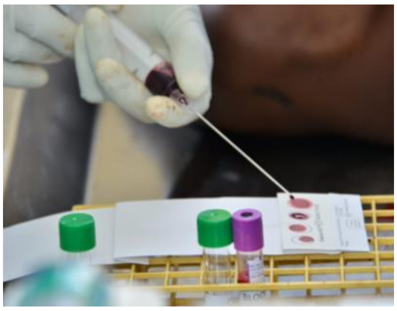

k. The Assistant fills the required information on the MITS Specimen Collection form.

Blood ☐ Y ☐ N
Total volume collected: ______(ml)
Location collected from:

**ensure that entire circle on blood spot card is filled with blood*	☐ Supraclavicular Heart<br>☐ Other: _________<br>1. Culture tube - 0.5-5 ml ☐ Y ☐ N<br>2. EDTA tube - 1.5-9 ml ☐ Y ☐ N<br>*invert EDTA tube after filling<br>3. Blood spot card circles ☐ Y ☐ N
l. The Assistant stores the EDTA tube and the 10 ml sterile tube in the MITS test tube rack and the blood spot card on the MITS tray..	

Collect blood In blood culture tubes (1 ml)

10.3. Lung/Thorax MITS for Microbiology

Materials Needed	MITS KIT Location
**Bard Monopty 16G 100mm (unlabeled)**	Kit Bag 1
**1 LUNG 2mL cryogenic vial (pre-labeled)**	Kit Bag 3

For Mol. Path. work (2 sets – loc and Cen Formalin jar):

MITS Specimen Collection

a. The Assistant takes and prepares the second, new, disposable Bard Monopty 16 G biopsy needle, and the LUNG cryogenic vials from the MITS kit.

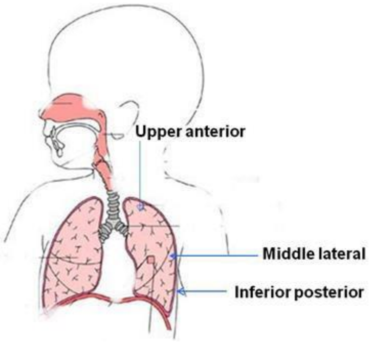

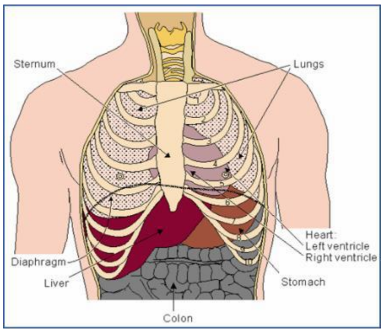

b. The Specialist performs the puncture with the automatic needle in the mid-axillary line, upper region of the right thorax, trying to obtain a lung sample from the upper lobe. The needle should be oriented towards the head. <u>The needle should penetrate as much as possible, for adults only,</u> and the movement of penetration should be quick in order to avoid iatrogenic pneumothorax and lung collapse. After reaching the limit, the needle should be retracted 2-3 cm, varies by body size.

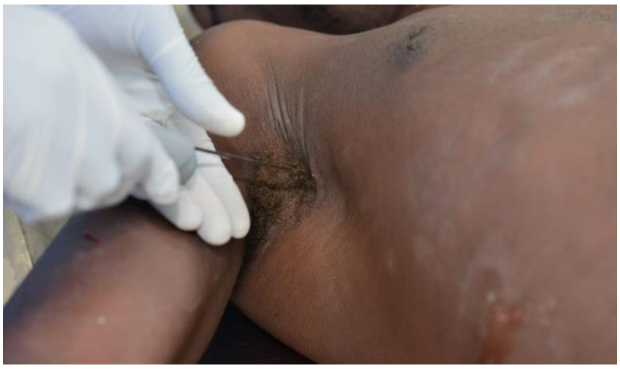

c. The Specialist engages the biopsy needle by pressing with the thumb the button at the back end of the handle.

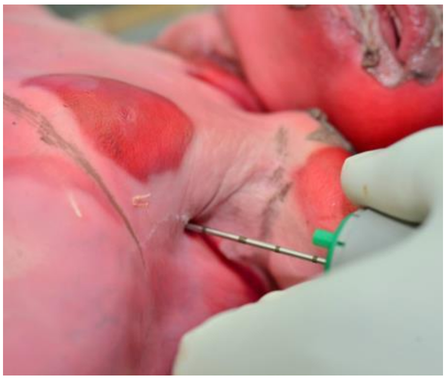

d. The specialist removes the needle from the body.

e. The specialist opens the needle to obtain the sample by rotating the handle in a clockwise direction.

f. The specialist checks that the tissue cylinder has been obtained from the correct organ (the cylinder should be about 20 x 1 mm and show a pink or reddish color and soft consistency

g. If there is no sample or the sample has not an adequate appearance (yellow color indicates fatty tissue, brown color and increased consistency may indicate liver tissue), the puncture should be repeated using the same needle until an adequate sample is obtained

h. The Specialist puts the tissue sample in the LUNG cryogenic vial

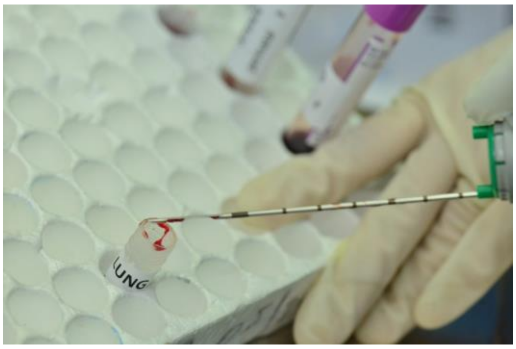

i. The Specialist repeats the sequence of punctures (repeat steps Section 9.6 Step a-j), aiming the needle in different directions to try to obtain samples from the upper, middle, and lower lung regions/lobes. Remove needle after a sample from each lobe is taken. Have to reset the needle for every sample.

One sample from each region should be obtained for the cryogenic vial. In the left thoracic region using the same needle and entry hole, and following the same procedure and placing the samples in the same LUNG cryogenic vial

For molecular pathology work.

Right Lung - Collect 6 Cores From (Upper: Middle: Lower :: 2 + 2 + 2)

RL Lung Combined - Collect 3 Cores From Upper: Middle: Lower :: 1 + 1 + 1)
Repeat above steps for CEN Tube

RL Combined M/B Lung Culture Tube - 3 cores from Upper, Middle, Lower :1 + 1 + 1

j. The Specialist repeats the same procedures in the left axillary region to obtain left lung samples, which are collected into the same LUNG cryogenic vial.

LEFT-SIDE LUNG

Left Lung - Collect 6 Cores From (upper: Middle: Lower: 2 + 2 + 2)

RL Lung Combined - Collect 3 Cores From Upper: Middle: Lower: 1 + 1 + 1)

Repeat Above Steps For CEN Tube

RL Lung Combined Microbiology & culture Tube - 3 Cores From (Upper: Middle: Lower::1 + 1 + 1)

k. The Assistant stores the LUNG cryogenic vial in the MITS cryogenic vial rack

l. The Assistant fills the required information on the MITS Specimen Collection Form

R & L lung – cryogenic vial (3 each) ☐ Y ☐ N

Note: The punctures of the lung can also be obtained using a posterior approach (back of the body, below the inferior angle of the scapula). Only use if difficulty with axillary approach.

- For this approach, the assistant should help the specialist to turn the body in a lateral or prone position.
- In this case, the biopsy needle should also be directed to the upper, middle and lower areas.

10.4. Lung/Thorax MITS for Histology

Materials Needed	MITS KIT Location
**Same needle as Section 9.6 (Lung/Thorax Microbiology)**	re-use from Section 9.6
**RIGHT LUNG formalin jar (pre-labeled)**	Kit Bag 4
**LEFT LUNG formalin jar (pre-labeled)**	Kit Bag 4

MITS Specimen Collection
a. The Assistant takes the RIGHT LUNG and LEFT LUNG formalin jars from the MITS kit.
b. The Specialist repeats the sequence of puncture approaches of the RIGHT and LEFT

Lung using the same entry points and following the same procedure.

c. The Specialist puts 2 samples of each right lung region (upper, middle, lower) in the RIGHT LUNG formalin jar and 2 samples of each left lung region (upper, middle, lower) area in the LEFT LUNG formalin jar (*a total of 12 samples, 6 from the right lung and 6 from the left lung*). Repeat Above Steps for CEN Jar

d. The Assistant stores the RIGHT LUNG and LEFT LUNG formalin jars in the MITS tray.

e. The Assistant fills the required information on the MITS Specimen Collection Form.

Left lung - formalin jar (6) ☐ Y ☐ N
Right lung - formalin jar (6) ☐ Y ☐ N

Discard Bard Biopsy Needle and

Take Bard Biopsy Needle Labeled For Brain

9.8 Placenta and fetal membranes and cord for microbiology

Note: Only for perinatal cases/still births if placenta is available

Collect the cord sample before proceeding for cleaning if it is a stillbirth case.

Materials Needed	MITS KIT Location
**Placenta cryogenic vial**	Backup Box Bag 1
**Membrane cryogenic vial**	Backup Box Bag 1
**5 cm measuring ruler**	Backup Box Envelope
**Scalpel blade #24 (2 blades)**	Backup Box Envelope
**Forceps (2 pair)**	Backup Box Bag 1
**MITS Placental Collection Form**	

Specimen Collection
**Review the Placental Gross Examination and Sampling SOP.<br>Take weight**
**Measure cord length**
**Take photos – fetal side, maternal side**
**Collect in sterile jar containing formalin. Label properly.<br>Make sure every tube/jar is labeled**

Note: These samples are only collected if skin lesions (papules, nodules, vesicles, macules) are detected.

a. The Assistant takes PLACENTA and MEMBRANE cryogenic vials from the MITS kit.

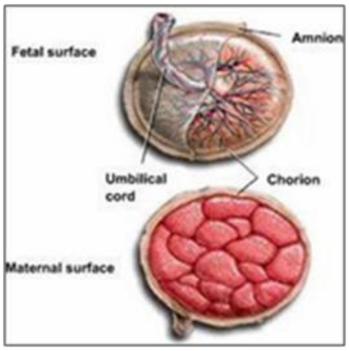

b. The Specialist cuts with the first scalpel blade two pieces of tissue 0.5 x 0.5 x 0.5 cm from the periphery of the placenta.

Take photos.
Make sure every tube/jar is labeled

c. Using the forceps, the specialist places two pieces in the PLACENTA cryogenic vial

d. Using a new scalpel blade, the specialist cuts two pieces of membrane

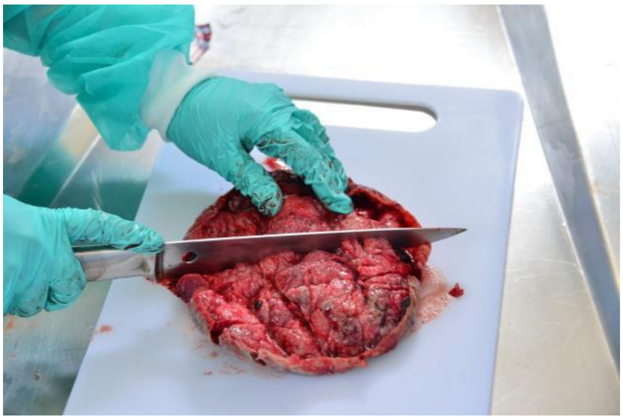

e. Using a new set of forceps, the specialist places two pieces in the MEMBRANE cryogenic vial
f. The Assistant puts the whole placenta in a container with 1.5-2L of 10% buffered neutral formalin and fills the required information on the Placenta Collection form.
h. The placenta will be sent to the pathology lab for gross examination and sampling for histology / **Take jar to histopathology lab.**

Transfer blood culture tube, CSF container, DBS card and EDTA lung culture tube to microbiology dept. at room temp.

End of the procedure and completion of the MITS Specimen Collection Form

10.5. MITS Specimen Collection Form

**After the MITS specimen collection procedure**<br>a. The Assistant writes the time (24 hour) in which the procedure has ended on the MITS Specimen Collection Form
b. The Assistant writes on the MITS Specimen Collection Form, by indication of The Specialist, any additional samples collected and/or notes from the procedure

10.6. Excessive seepage or bleeding though the biopsy entry points

**After the MITS specimen collection**
**Note: If body fluid leakage is a social concern in your community, you can use Monsel's solution (ferric subsulfate 20%)/ Tincture Benzene to stop excessive bleeding or seepage if it occurs during MITS collection. Monsel's solution is not included in the MITS kit.**
a. The Assistant and Specialist check for excessive seepage or bleeding.

b. The Assistant prepares the container with the ~~Monsel's solution~~/Tincture Benzene and a swab for its application and opens the ~~Mosel's solution jar~~/Tincture Benzene.

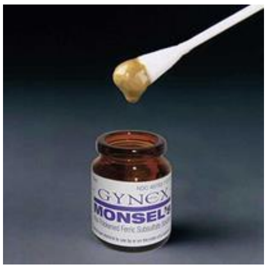

c. The Assistant takes with the swab some ~~Monsel's solution~~/Tincture Benzene from the jar.

d. The Assistant applies the ~~Monsel's solution~~/Tincture Benzene to the bleeding entry point.

e. If necessary, take a gauze and roll it around the bleeding area to make pressure and reinforce the hemostatic effect of ~~the Monsel's solution~~/Tincture Benzene. Before delivering the body to the family REMOVE the gauze.

10.7. Containers and Tools

After the MITS specimen collection	
a. The Assistant makes sure that all the containers and jars are properly labeled and closed.	☐ Confirm all the containers and jars are properly labeled and closed.
b. The Specialist and Assistant make sure that any unused and unlabeled materials (*e.g. swabs*) are stored in a backup box and any unused materials labeled with an ID are disposed of in the biowaste container.	☐ Confirm any unused labeled cryovials and jars are disposed of in a biowaste container.
c. The Specialist and Assistant dispose of any sharps in sharps container.	☐ Confirm all sharps are disposed in sharp container.
d. The Assistant puts the MITS cryogenic vial rack with all the used cryogenic vials and the MITS test tube rack with the used tubes in the liquid nitrogen transfer vessel.	☐ Confirm MITS rack with all the used cryovials in the **liquid nitrogen transfer vessel.**
e. The Assistant puts the MITS tray with all the used formalin jars, the unused large screw-cap jar, and any MITS tissue cassettes in the MITS kit box.	☐ Confirm MITS tray with used formalin jars, the unused large screw-cap jar, any tissue cassettes in the MITS kit box.
**Note: The** liquid nitrogen transfer vessel. **(microbiology) and the MITS kit box (pathology) should be sent for further processing**	
e. The Assistant washes the permanent marker with 70% ethanol and the surfaces used during the process with the standard 10% bleach followed by 70% ethanol to dry.	☐ Confirm all used surfaces are washed.
f. The Assistant puts away the digital camera.	☐ Confirm digital camera is stored.

Storage in liquid nitrogen of samples described separately.

Clean body properly.
Seal puncture wounds properly.
Hand-over the child to parents/attendants and thank them.

10.8. MITS Envelope

After the MITS specimen collection

a. The Assistant puts the Specimen Collection Form back into the MITS envelope

b. The Specialist makes sure that the project-specific gross examination form and pathology form, remaining labels ,and the 5 cm ruler are in the MITS envelope.

c. The Assistant puts the MITS envelope back into the MITS kit box

11. References

Castillo P, Ussene E, Ismail MR, Jordao D, Lovane L, Carrilho C, et al. Pathological Methods Applied to the Investigation of Causes of Death in Developing Countries: Minimally Invasive Autopsy Approach. PLoS One. 2015 Jun 30; 10(6):e0132057.

Martínez MJ, Massora S, Mandomando I, Ussene E, Jordao D, Lovane L, et al. Infectious cause of death determination using minimally invasive autopsies in developing countries. Diagn Microbiol Infect Dis. 2016; 84(1):80-6.

CDC. National health and nutrition examination survey (NHANES). Anthropometry procedures manual. January 2011

12. Appendices

12.1. Appendix A: MITS Kit Components

Note: This is project-specific. These are the contents of the MITS Surveillance Alliance MITS training kit

	**ENVELOPE**	**UNITS**
**E.1**	MITS Specimen Collection Form (pre-labeled)	1
**E.2**	Blood spot card (pre-labeled)	1
**E.3**	Photo card (pre-labeled)	1
**E.4**	Labels for: extras samples, NP swab, slides	1
	**NO BAG**	**UNITS**
**0.1**	Tape Measure	1
**0.2**	Disposable placemat	2
	**BAG 1 – Biopsy needles, swabs, brushes**	**UNITS**
**1.1**	Bard Monopty needle 16G, 100mm	2
**1.2**	Bard Monopty needle 16G, 160mm (labeled with	1
	BRAIN)	
**1.3**	NP swab tube with viral transport media (individually wrapped, unlabeled)	1
	**BAG 2 – Needles, syringes**	**UNITS**
**2.1**	20G spinal puncture needle (Yellow)	2
**2.2**	18G spinal puncture needle (Pink)	2
**2.3**	20mL syringes	2
**2.4**	16G hypodermic needle (Purple)	2
	**BAG 3 – Vacutainer, vials**	**UNITS**
**3.1**	10mL EDTA vacutainer (purple top) (pre-labeled)	1
**3.2**	10mL cryogenic storage vial (prelabeled)	3
**3.3**	2mL cryogenic storage vials (pre-labeled)	3
	**BAG 4 – Cassettes, jars**	**UNITS**
**4.1**	Large (120 ml) screw cap jar (pre-labeled)	1
**4.2**	Cassettes (unlabeled)	4
**4.3**	20 mL jars (prefilled 10% formalin) (pre-labeled)	4

12.2. Appendix B: Back-up box Components

Note: This is project-specific. These are the contents of the MITS Surveillance Alliance MITS training backup box

	ENVELOPE	UNITS
**E.1**	Millimeter ruler (5 cm)	2
**E.2**	Photo card	1
**E.3**	Scalpel blade #24	2
	**NO BAG**	**UNITS**
**0.1**	Tape Measure	1
**0.2**	Disposable placemat	1
**0.3**	Bone Marrow Trephine	4
	**Bag 1**	**UNITS**
**1.1**	Forceps	2
**1.2**	2mL cryogenic storage vials	4
**1.3**	Biopsy punch needle 5mm	1
**1.4**	20 mL jars (prefilled 10% formalin) (pre-labeled)	4
**1.5**	White tissue cassette (unlabeled)	4
**1.6**	Scalpel handle	1

12.3. Appendix C: Table for formalin jars and cryogenic vials

I. Formalin jars (Histology)

Tissue Specimen Description	Label Sample ID	Label Text	Label location
**Brain CNS biopsies for histology**	1##-###-03-J	Brain/CNS	Pre-applied to formalin jar
**Left lung/thorax biopsies for histology**	1##-###-05-J1	L Lung/Thorax	
**Right lung/thorax biopsies for histology**	1##-###-05-J2	R Lung/Thorax	
**Placenta and cord biopsies for histology**	1##-###-08-J	Placenta	In Kit envelope
**Placenta membrane biopsies for histology**	1##-###-09-J	Membrane	

II.

Croygenic Vials (Microbiology)

Tissue Specimen Description	Label Sample ID	Label Text	Label location
**Brain CNS biopsies for microbiology**	1##-###-03-V	Brain/CNS	Pre-applied to formalin jar
**Left Lung/thorax biopsies for microbiology**	1##-###-05-V	L Lung/Thorax	
**Placenta and cord biopsies for microbiology**	1##-###-08-V	Placenta	In Kit envelope
**Placenta membrane biopsies for microbiology**	1##-###-09-V	Membrane	

III. Cassettes and Slides (Histology)

Tissue Specimen Description	Label Sample ID	Label Text	Label location
**Brain CNS histology slides**	1##-##1-03-S	Brain/CNS	In Kit envelope
**Left lung/thorax histology slides**	1##-##1-05-S1	L Lung/Thorax	
**Right lung/thorax histology slides**	1##-##1-05-S2	R Lung/Thorax	
**Placenta and cord histology slides**	1##-##1-08-S	Placenta	
**Placenta membrane histology slides**	1##-##1-09-S	Membrane	

Minimally Invasive Tissue Sampling (MITS) Standard Operating Procedure (SOP) (MAHAN Trust)
