# Supplementary material for: Community—Minimal Invasive Tissue Sampling (cMITS) using a modified ambulance for ascertaining the cause of death: A novel approach piloted in a remote inaccessible rural area in India
Source: Arch Public Health. 2023 Apr 27;81:72. doi: 10.1186/s13690-023-01062-x (PMC10134564; doi:10.1186/s13690-023-01062-x)
Supplement: Supplementary file 5 — Additional file 5: Annexure 5: SOP for sample storage and transport. [file 13690_2023_1062_MOESM5_ESM.zip › 1-Annexure 5_SOP for sample storage and transport.pdf]

## SOP for sample transportation and storage

(1st May 2020 to 30th April 2021)
(Dharni Block of Amaravati district)

Samples collected by MITS procedure in Ambulance

Samples will be collected for three purposes:

**A) Microbiology, Culture**

A) Blood culture & CSF sample:
Sterile tube:
DBT card (HIV):
Blood EDTA tube
Culture bottles.

**B) PCR**

B) Two cryovials: one for left and right lung and one for CNS. One cryovial for liver
One sterile tube: CSF
Nasopharyngeal swab

**C) Histopathology**

**C) Four formalin jars**: one for right lung, one for left lung, one for CNS and one for liver

### Sample transportation from MITS ambulance to base hospital (MAHAN)

A) All samples will be carried in Icebox

B) All samples will be carried in 5 Liquid nitrogen cylinder (<-80$^{0}$C to -160$^{0}$C) and naso- pharyngeal swab in icebox

C)All samples will be carried at room temperature

### Sample storage at base hospital (MAHAN)

A) HIV, Malaria: MAHAN Lab. Blood culture bottle, DBT card at room temperature,
EDTA tube and CSF sample in refrigerator

B) All PCR samples will be stored in 47 L nitrogen cylinder (<-80$^{0}$C to -160$^{0}$C), nasopharyngeal swab in -20°C Freezer

C)All samples will be stored at room temperature in 10% formalin for 8-24 hours and then in 70-80% Isopropyl alcohol

A) Samples will be carried in Ice box from MAHAN to Nagpur in MITS AMBULANCE

B) Samples will be carried in Liquid nitrogen cylinder (<-80$^{0}$C to -160$^{0}$C ) (for PCR) from MAHAN to Nagpur in MITS AMBULANCE

C)Samples will be carried in 70-80% alcohol at room temperature from MAHAN to Nagpur in MITS AMBULANCE

B) Samples will be transported in dry ice (<-80$^{0}$C) to CMC Vellore/NIV Pune by air courier/train

B)Samples will be transported in 70-80% alcohol at room temperature by air courier/train

A) All samples will be processed at Vishakha Clinical Microbiology laboratory, Nagpur.

B) All PCR samples will be processed in NIV Pune

C)All samples will be processed in NIOP, Delhi

All samples will be labelled as BIOHAZARD while transportation

## Cryovials storage and transport

1) Collect the tissue samples from dead body in cryovials.
2) Immediately transfer these cryovials to cryo-box which is kept in 5L Mini liquid nitrogen container (temperature <-80$^{0}$C to -160$^{0}$C) in MITS Van.
3) Use special gloves, googles, face shields, gown and shoes to prevent damage to person handling liquid nitrogen.
4) After reaching the hospital, transfer the cryovials and cryo-box to Cryo racks which are kept in the 45/60L liquid nitrogen container (temperature <-80$^{0}$C to -160$^{0}$C) (Thermo Fisher Scientific Cat. No. 11679077). The cryovials will be stored in vapor phase of liquid nitrogen and not in liquid phase of liquid nitrogen.
5) The liquid nitrogen level in nitrogen container will be regularly monitored with the help of level monitor. The liquid nitrogen will be topped up once in 15 to 21 days depending upon level of liquid nitrogen in nitrogen container. The liquid nitrogen will be brought from Amravati regularly in nitrogen transfer container.
6) **Transport of cryovials to designated laboratory**: After collection of samples from 8 to10 dead children, our team will remove the cryovials and cryo-boxes from the liquid nitrogen container by using personnel protection measures ( wearing special gloves, googles, face shields, gown and shoes). The cryovials and cryo-boxes will be transferred to liquid nitrogen transfer containers (temperature <-80$^{0}$C to -160$^{0}$C). The liquid nitrogen transfer container will be shifted to Nagpur airport in ambulance. In Nagpur, the cryovials will be transferred to dry ice (temperature <-80$^{0}$C). The cryovials in dry ice will be shifted to designated laboratory by air courier so that it will reach laboratory within 24 hours.

## Alert/Precautions for use of cryovials

Cryogenic vials are intended for placement only in the vapor phase of liquid nitrogen, and should not be used for storage in the liquid phase of liquid nitrogen. Immersion of the vials in the liquid phase could result in penetration of the liquid gas into the vial, resulting in rapid vaporization of the liquid upon removal and possible violent explosion or leakage from the vial/closure perimeter. To prevent cryogenic vials from exploding, never over fill liquid nitrogen storage units. Always examine vials before use to ensure no visible defects around the closure rims. Always use full face-shields, heavy safety gloves and laboratory protective apparel when removing vials from cryogenic storage. Always permit vials to warm slowly in a biological safety cabinet. Never reuse cryogenic vials.
