# Supplementary material for: Community—Minimal Invasive Tissue Sampling (cMITS) using a modified ambulance for ascertaining the cause of death: A novel approach piloted in a remote inaccessible rural area in India
Source: Arch Public Health. 2023 Apr 27;81:72. doi: 10.1186/s13690-023-01062-x (PMC10134564; doi:10.1186/s13690-023-01062-x)
Supplement: Supplementary file 6 — Additional file 6: Annexure 6: Verbal autopsy form for neonatal deaths (0 to 28 days). [file 13690_2023_1062_MOESM6_ESM.pdf]

MAHAN TRUST MELGHAT

VERBAL AUTOPSY FORM FOR NEONATAL DEATHS (0 to 28 DAYS)

Date: (dd/mm/yyyy) _____/_____/___________ **Time:**_____________

(1st May 2020 to 30th April 2021) (Dharni Block of Amaravati district)

I. GENERAL INFORMATION ABOUT FAMILY

Name of head of the household ____

Identification code of head of the household: ☐☐☐☐☐☐☐☐☐

Name of deceased____

Identification code of deceased child: ☐☐☐☐☐☐☐☐☐

Name of mother of deceased child____

Identification code of mother of deceased: ☐☐☐☐☐☐☐☐☐

Name of father of deceased child____

Identification code of father of deceased: ☐☐☐☐☐☐☐☐

1) **Sex of baby:** ☐ 1. Male ☐ 2. Female

2) **Date of Birth** (dd/mm/yyyy) : ___ ___ / ___ ___ / ____ ____ ____ ____

3) **Date of Death** (dd/mm/yyyy) : ___ ___ / ___ ___ / ____ ____ ____ ____

4) **Age at the time of Death:**
[If baby lived less than 24 hours enter hours, otherwise. enter age in days up to 28 days]
Days________ **OR** Hours________

5) **Where was the place of death?**

☐1. Home ☐2. On way to health facility ☐3. PHC/CHC/Rural Hospital
☐4. District Hospital ☐5. Private Hospital ☐6. Other place
☐99. Don't know

II. <u>DETAILS ABOUT RESPONDENTS</u>

6) Name of respondent: ____

7) Relation with deceased:

☐1. Parents (Mother/father) ☐4. Sister
☐2. Grandparents ☐5. Other relative
☐3. Brother ☐6. Neighbors/No relation
☐99. Don't know

8) Age of respondent:______years

9) Age of mother at time of baby's death:______years

10) Sex of respondent: ☐ 1. Male ☐ 2. Female

11) Education of Respondent:

☐ 0.Illiterate/No formal education ☐ 3.SSC
☐ 1.Primary(1 to 4th standard) ☐ 4.HSSC
☐ 2. Middle(5th to 9th standard) ☐ 5.Graduate & above
☐ 99. Don't know

12) Did the respondent live with the deceased during the events that led to death?
☐1. Yes ☐ 2. No ☐ 99. Don't know

13) Was the deceased a singleton or multiple birth?
☐1. Singleton ☐2. Twin ☐3. Triplet ☐99. Don't know

14) If multiple birth, was the baby second born?
☐ 1. Yes ☐2. No ☐ 99. Don't know

15) Was this the first, second, or later in the birth order?
☐ 1. First ☐ 2. Second ☐3. Third or more ☐ 99. Don't know

16) Was this the first delivery of the mother? Whether mother was primigravida?
☐ 1. Yes ☐2. No ☐ 99. Don't know

17) How many births, including stillbirths, did the mother have before this baby?
Number of births/stillbirths__________ ☐ 99. Don't know

18) Did the mother receive antenatal care?

☐ 1. During ☐ 2. After ☐ 99. Don't know

19) Is the mother still alive? ☐ 1.Yes ☐ 2. No

If "Yes", go to question 22.

20) Did the mother die during or after the delivery?

☐ 1. During ☐ 2. After ☐ 99. Don't know

21) How long after the delivery did the mother die?

__ __ days **OR** __ __ months

22) Was the late part of the pregnancy (defined as the last 3 months), labor, or delivery complicated by any of the following problems? *(Read each complication and mark all that apply.)* *(Read "the mother" if the mother is not the respondent.)*

☐ 12. No complications
☐ 1. You (the mother) had convulsions
☐ 2. You (the mother) had high blood pressure
☐ 3. You (the mother) had severe anemia
☐ 4. You (the mother) had diabetes
☐ 5. Child delivered not head first
☐ 6. Cord delivered first
☐ 7. Cord around child's neck
☐ 10. Excessive bleeding
☐ 11. Fever
☐ 13. Blurred vision
☐ 14. Heart disease
☐ 15. Vaginal bleeding
☐ 16. Smelly vaginal discharge
☐ 17. Puffy face
☐ 18. Headache
☐ 19. Severe abdominal pain that was not labor pain
☐ 20. Pallor or shortness of breath (both present)
☐ 21. Other illnesses
☐ 99. Don't know

23) Was there any difficulty during labor?

☐ 1. Yes ☐ 2. No ☐ 99. Don't know

24) If yes, what was the nature?

____
____
____
____

25) What was presenting part of baby during labour? *(Read the choices and mark ONE.)*

☐ 1. Head ☐ **2.** Face ☐ 3. Hand ☐ 4. Foot ☐ 5. Buttock

☐ 6. Umbilical cord ☐ **7.** Other______________

26) Was the delivery…? *(Read the choices and mark ONE.)*

☐ 1. Vaginal with forceps ☐ 2. Vaginal w/out forceps

☐ 3. Vaginal (Don't know if forceps or not) ☐ 4. C-Section

☐99. Don't know

27) Was internal manipulation done during delivery?

☐ 1. Yes ☐2. No ☐ 99. Don't know

28) Was the baby moving in the last few days before the birth?

☐ 1. Yes ☐2. No ☐ 99. Don't know

29) Did the baby stop moving in the womb before labour started?

☐ 1. Yes ☐2. No ☐ 99. Don't know

30) When did the baby stop moving in the womb?

☐ 1. Before labor started ☐ 2. During labor ☐99. Don't know

31) When did the water break?

☐ 1. Before labor started ☐ 2. During labor ☐99. Don't know

32) How many hours after the water broke was the baby born?

☐ 1. Less than 24 hours ☐ 2. 24 hours or more ☐99. Don't know

33) What was the color of the liquor when the water broke?

☐ 1. Green or brown ☐ 2. Clear (normal) ☐ 3. Other (___________________)

☐99. Don't know

34) Was the liquor foul smelling?

☐ 1. Yes ☐2. No ☐99. Don't know

35) How much time did the labor and delivery take? *(Less than 1 hour == "00")*

___ ___ hours

36) Did the birth attendant listen for fetal heart sounds during labor?

☐ 1. Yes ☐2. No ☐ 99. Don't know

37) Were fetal heart sounds present?

☐ 1. Yes ☐ 2. No ☐99. Don't know

38) Was there excess bleeding on the day labor started?

☐ 1. Yes ☐2. No ☐ 99. Don't know

39) Did the mother have a fever on the day labor started?

☐ 1. Yes ☐2. No ☐ 99. Don't know

40) Was the mother suffering from fever within 7 days after the delivery?

☐ 1. Yes ☐2. No ☐ 99. Don't know ☐99. Don't know

41) If Yes, for how long did the fever persists?

☐1. Less than 24 hours ☐2. More than 24 hours ☐99. Don't know

(Id10406) Was the baby blue in colour at birth?

III. Details of birth

42) Birth Place:

☐ 1. in house ☐ 2. in hospital ☐ 3. on road ☐ 4. other place

43) Who conducted delivery:

☐ 1. Traditional birth attendant (if yes, answer question 44)
☐ 2. Relative/friends (if yes, answer question 45)
☐ 3. Nurse (if yes, answer question 46)
☐ 4. Doctor (if yes, answer question 47)
☐ 5. Other (specify__________________________) (if yes, answer question 47)
☐ 6. Self (the mother)

44) Was the traditional birth attendant who conducted the delivery trained?

☐ 1. Yes ☐2. No ☐ 99. Don't know Name__________________

45) Was relative/friends who conducted the delivery trained?

☐ 1. Yes ☐ 2. No ☐ 99. Don't know Name___________________

46) Was the nurse who conducted the delivery trained?

☐ 1. Yes ☐ 2. No ☐ 99. Don't know Name___________________

47) Was the doctor who conducted the delivery trained?

☐ 1. Yes ☐ 2. No ☐ 99. Don't know Name___________________

48) Was the other person who conducted the delivery trained?

☐ 1. Yes ☐ 2. No ☐ 99. Don't know Name___________________

IV. <u>GENERAL INFORMATION ABOUT FAMILY</u>

49) Was the baby alive at the time of birth?

(Consider alive If following signs are seen: Breathing, Crying, Chest-Wall movement, Heart Beats, Eye Movement, Movement of limbs.)

☐1. Yes ☐2. No ☐ 99. Don't know

50) Did the baby ever cry?

☐1. Yes ☐2. No ☐ 99. Don't know

If no, skip to Q56

51) If yes, how many minutes after birth did the baby cry? ______________minutes

☐99. Don't know

52) **What efforts were required to make baby cry?**

☐1. None, cried on its own.
☐2. Slapping of feet or back
☐3. Splashing water on baby
☐4. Holding the baby upside down
☐99. Don't know

53) How loud was cry of baby at birth?

☐ 1. Loud ☐ 2. Feeble ☐ 99. Don't know

54) Did the baby stop being able to cry?

☐ 11. Yes ☐ 2. No ☐ 99. Don't know

55) How many hours before death did the baby stop crying?
[60 minutes=1 hour; if < 1 hour, record "0" hours.]
_______________ Hours

56) Did the baby ever move?
☐ 1. Yes ☐ 2. No ☐ 99. Don't know

If no, skip to Q61

___ ___ days OR ___ ___ hours ☐ 99. Don't know

57) How many <u>days before labor</u> did you or the mother last feel the baby move?
___ ___ days ☐ 99. Don't know

58) How many <u>hours before labor</u> did you or the mother last feel the baby move?
___ ___ hours ☐ 99. Don't know

59) Did the baby ever breathe?
☐ 1. Yes ☐ 2. No ☐ 99. Don't know ☐ 99. Don't know

If no, skip to Q64

60) If yes, how many minutes after birth did the baby breathe?
______________minutes ☐ 99. Don't know

61) Did the baby have difficulty breathing?
☐ 1. Yes ☐ 2. No ☐ 99. Don't know

62) Was anything done to try to help the baby breathe at birth?
☐ 1. Yes ☐ 2. No ☐ 99. Don't know

If the baby was stillbirth, then go to question ________
If the baby was born alive, go to question _______

63) Were there any bruises or signs of injury or broken bones on the baby's body at birth?
☐ 1. Yes ☐ 2. No ☐ 99. Don't know

64) Was the baby's body (skin and tissue) pulpy?

☐ 1. Yes ☐ 2. No ☐ 99. Don't know

65) Was any part of the baby physically abnormal at time of delivery? (for example: body part too large or too small, additional growth on body)

☐ 1. Yes ☐ 2. No ☐ 99. Don't know

If "No" or "Refused to answer" or "Don't know", go to question_______

66) What were the abnormalities? (MARK ALL THAT APPLY)

☐ 1. Head size very small at time of birth
☐ 2. Head size very large at time of birth
☐ 3. Mass defect on the back of head or
☐ 4. Other (Specify____)
☐ 99. Don't know

V. <u>GENERAL INFORMATION ABOUT FAMILY</u>

67) How old was the baby/child when the fatal illness started?

(Less than 24 hours = 00 days, otherwise enter age in days }

___ ___ days ☐ 99. Don't know

68) How long did the illness last?

(Less than 24 hours = 00 days.)

___ ___ days

69) Before the illness that led to death, was the baby growing normally?

☐ 1. Yes ☐ 2. No ☐ 99. Don't know

70) Did s/he die from an injury or accident?

☐ 1. Yes ☐ 2. No ☐ 99. Don't know

If no, go to next section to Q77

71) If yes, what kind of injury or accident?

☐ 1. Road traffic accident
☐ 2. Falls
☐ 3. Fall of objects
☐ 4. Burns/fire
☐ 5. Drowning

☐ 6. Poisoning
☐ 7. Bite/sting
☐ 9. Natural disaster
☐ 10. Homicide/assault
☐ 11. Animal/insect
☐ 12. Firearm
☐ 13. Stabbed/cut/pierced
☐ 14. Strangled
☐ 15. Blunt force
☐ 16. Force of nature
☐ 17. Electrocution
☐ 18. Other ______________________________________
☐ 99. Don't know

72) Was the injury accidental?
☐ 1. Yes ☐2. No ☐ 99. Don't know

73) Was the injury or accident intentionally <u>inflicted by someone else</u>?
☐ 1. Yes ☐2. No ☐ 99. Don't know

74) If (s)he died in a road accident, what was his/her role in the road traffic accident?
☐1. Pedestrian
☐2. Passenger in car or light vehicle
☐3. Passenger in bus or heavy vehicle
☐4. Passenger on a motorcycle
☐5. Passenger on a pedal cycle
☐6. Other

75) If (s)he died of an animal/insect bite, what type of animal/insect?
☐ 1. Dog ☐ 2. Snake ☐ 3. Insect ☐ 8. Other ___________
☐ 99. Don't know

76) Was there any sign of paralysis?
☐ 1. Yes ☐2. No ☐ 99. Don't know

77) Was the baby able to suckle in a normal way during the <u>first day of life</u>?
☐ 1. Yes ☐2. No ☐ 99. Don't know

78) Did the baby <u>ever</u> suckle in a normal way?
☐ 1. Yes ☐2. No ☐ 99. Don't know

79) If no, how many days after birth did the baby stop suckling?__

_________________days ▢99. Don’t know

VI. RESPIRATORY SYMPTOMS

80) During the illness that led to death, did the baby have difficult breathing/breathlessness?

▢ 1. Yes ▢2. No ▢ 99. Don’t know

If no, go to Section Q82 & Q83

81) How many hours after the birth did abnormal breathing signs/symptoms appear?

▢ 1. within six hours ▢2. up to six hours ▢3. after six hours

▢99. Don’t know

82) For how many days did the difficult breathing/breathlessness last? ***(Less than 1 day= “00”)***

___ ___ days ▢99. Don’t know

83) During the illness that led to death, did the baby have fast breathing?

▢ 1. Yes ▢2. No ▢ 99. Don’t know

84) For how many days did the fast breathing last? ***(Less than 1 day= “00”)***

___ ___ days ▢99. Don’t know

85) During the illness that led to death, did the baby have indrawing of the chest?

▢ 1. Yes ▢2. No ▢ 99. Don’t know

86) Did the baby have flaring of the nostrils?

▢ 1. Yes ▢2. No ▢ 99. Don’t know

87) During the illness that led to death did his/her breathing sound abnormal?

▢ 1. Stridor ▢ 2. Grunting ▢3. Wheezing ▢4. No

▢ 99. Don’t know

88) During the illness that led to death, did the baby become cold to touch?

▢ 1. Yes ▢2. No ▢ 99. Don’t know

If no, skip Q90-Q92

89) At what age did the baby start feeling cold to touch? *(Less than 1 day= “00”)*

___ ___ days ▢99. Don’t know

90) For how many hours long was baby's body cold before death? (____________)

91) Which part of the body was cold?

☐ 1. Head ☐ 2. Chest, Abdomen, Hand and/or leg ☐ 3. Other

☐ 99. Don't know

92) During the illness that led to death, did the baby become lethargic?

☐ 1. Yes ☐ 2. No ☐ 99. Don't know

93) Was there normal activity before the baby became lethargic?

☐ 1. Yes ☐ 2. No ☐ 99. Don't know

94) During the illness that led to death, did the baby become unresponsive or unconscious?

☐ 1. Yes ☐ 2. No ☐ 99. Don't know

95) How long before death did the baby become unresponsive or unconscious?

__ __ hours **OR** __ __ days

96) How long after birth did the baby become unresponsive or unconscious?

__ __ hours **OR** __ __ days

97) During the illness that led to death, did the baby have redness or pus drainage from the umbilical cord stump?

☐ 1. Yes ☐ 2. No ☐ 99. Don't know

98) During the illness that led to death, did the baby have an area(s) of skin with redness and swelling?

☐ 1. Yes ☐ 2. No ☐ 99. Don't know

99) During the illness that led to death, did he/she have yellow skin?

☐ 1. Yes ☐ 2. No ☐ 99. Don't know

100) During the illness that led to death, did he/she have areas of skin turn black?

☐ 1. Yes ☐ 2. No ☐ 99. Don't know

101) During the illness that led to death, did he/she have any skin rash?

☐ 1. Yes ☐ 2. No ☐ 99. Don't know

102) During the illness that led to death, did he/she have skin ulcer(s) or pits?

☐ 1. Yes ☐ 2. No ☐ 99. Don't know

103) During the illness that led to death, did he/she have yellow discoloration of the eyes?

☐ 1. Yes ☐ 2. No ☐ 99. Don't know

104) Did the infant appear to be healthy and then just die suddenly?

☐ 1. Yes ☐ 2. No ☐ 99. Don't know

105) Did the pregnancy end earlier than expected?

☐ 1. Yes ☐ 2. No ☐ 99. Don't know

106) What was gestational age? (Start counting pregnancy in weeks from last menstrual period)

_________weeks ☐ 99. Don't know

107) How did baby die? Describe in details about the problem at the time of death (as per narration of mother.) Avoiding the terms like black magic, superstitions, etc, ask the parents regarding signs & symptoms of the baby.

____
____
____
____
____
____
____
____
____
____
____
____
____

108) What was the size of the baby at the time of birth?

☐ 1.Very small ☐ 4. Larger than average

☐ 2. Smaller than average ☐ 8. Refused to answer

☐ 3.Average ☐ 99.Don't know

109) What was the weight at the time of birth: ___________gm ☐ 99. Don't know

110) What were the color of limbs and the lips of the baby after birth?

☐ Pink-Normal ☐ Blue

111) Was there any gross swelling/mark on the scalp?

☐ 1. Yes ☐ 2. No ☐ 99. Don't know

112) Was there any convulsions after birth?

☐ 1. Yes ☐ 2. No ☐ 99. Don't know

113) If yes, how soon after birth?______________________ days

114) Was the baby limp? (within first 3 days)

☐ 1. Yes ☐ 2. No ☐ 99. Don't know

V) <u>Tetanus</u>

115) Was the body of baby stiff or hyperextended back like bow?

☐ 1. Yes ☐ 2. No ☐ 99. Don't know

116) Did the baby have Lock jaw?

☐ 1. Yes ☐ 2. No ☐ 99. Don't know

117) Did the mother receive Tetanus Toxoid Vaccine during this pregnancy?

☐ 1. Yes ☐ 2. No ☐ 99. Don't know

118) If yes, how many doses?

Number of doses__________ ☐ 99. Don't know

119) Did you/the mother receive any vaccinations since reaching adulthood including during this pregnancy?

☐ 1. Yes ☐ 2. No ☐ 99. Don't know

120) What instrument was used to cut the umbilical cord?

☐ 1. Boiled/ Sterile blade/ New blade ☐ 2. Uncleaned blade or other things

☐ 8. Refuse to answer ☐ 99. Don't know

121) What was used to tie the umbilical cord?

☐ 1. Unboiled thread ☐ 2. rubber band ☐ 3. **Boiled thread**

122) What was applied over umbilical stump?

☐ 1. Antiseptic lotion ☐ 2. oil/ vermilion /cow dung / Brick

123) Was there periumbilicalswelling/redness or purulent umbilical discharge?

☐ 1. Yes ☐2. No ☐ 99. Don't know

124) Whether TBA washed hands with soap &clean water before conducting delivery?

☐ 1. Yes ☐2. No ☐ 99. Don't know

125) Did the family members recognize it as Tetanus?

☐ 1. Yes ☐2. No ☐ 99. Don't know

VI) R. D. S. / A. R. I. Pneumonia

126) Was there cough before death?

☐ 1. Yes ☐2. No ☐ 99. Don't know

If no, skip Q128 & Q129

127) Did the baby make a whooping sound when coughing?

☐ 1. Yes ☐2. No ☐ 99. Don't know

128) How many days after birth did the baby start to cough?

☐ 1. ____hours ☐ 2. __ __ days ☐3. ____ weeks

129) Was the baby suffering from fever?

☐ 1. Yes ☐2. No ☐ 99. Don't know

130) If yes, for how many days did the fever last? ___________ Days

VII) Diarrheal diseases

131) Was the baby suffering from loose motion?

☐ 1. Yes ☐2. No ☐ 99. Don't know

If no, skip Q133-Q135

132) If yes, then how many times in one day & night?________________________

133) How many days before death did the frequent loose or liquid stools start?

☐ 1. _______days ☐ 99. Don't know

134) What was the maximum number of episodes of loose motion in 24 hours by the child?_____________

135) Was the stool sticky, containing mucus and blood?

☐ 1. Yes ☐ 2. No ☐ 99. Don't know

136) Was the baby suffering from vomiting?

☐ 1. Yes ☐ 2. No ☐ 99. Don't know

137) Did the baby vomit in the week preceding death?

☐ 1. Yes ☐ 2. No ☐ 99. Don't know

138) Was the breast feeding continued?

☐ 1. Yes ☐ 2. No ☐ 99. Don't know

139) Was the breastfeeding exclusive?

☐ 1. Yes ☐ 2. No ☐ 99. Don't know

140) How was (anterior) fontanelle?

☐ 1. Plain/ normal ☐ 2. Depressed inside ☐ 3. bulged/raised ☐ 99. Don't know

141) If s(he) has bulging fontanelle, for how many days before death?

_______days ☐ 99. Don't know

142) Were eyes sunken?

☐ 1. Yes ☐ 2. No ☐ 99. Don't know

143) How much was Urine output?

☐ 1. Normal ☐ 2. Less/Nothing ☐ 3. Don't know

144) How was color of urine?

☐ 1. Watery/Normal ☐ 2. Yellow ☐ 3. Don't know

VIII) Hypothermia

145) Was the baby suffering from hypothermia?

☐ 1. Yes ☐ 2. No ☐ 99. Don't know

146) Was there any boil or pustule on the body?

☐ 1. Yes ☐ 2. No ☐ 99. Don't know

147) Was the baby suffering from Vomiting?

☐ 1. Yes ☐ 2. No ☐ 99. Don't know

148) Was the baby suffering from loose motions more than 3 times a day?

☐ 1. Yes ☐ 2. No ☐ 99. Don't know

149) Was there abdominal distention?

☐ 1. Yes ☐ 2. No ☐ 99. Don't know

150) Did the breathing of the child stop for some time in between convulsions? (While after the birth, breathing was normal)

☐ 1. Yes ☐ 2. No ☐ 99. Don't know

151) Was there bleeding from the skin or body parts orifices?

☐ 1. YES ☐ 2. NO ☐ 8. Refuse to answer ☐ 99. Don't know

XI) Breast feeding Problem

152) Did the baby breast feed within the last 2 days before the death?

☐ 1. Yes ☐ 2. No ☐ 99. Don't know

153) Did the baby suck & swallow milk during last 2 days before death?

☐ 1. Yes ☐ 2. No/sucking little milk ☐ 99. Don't know

154) Was the baby fed bottle milk or top feed because of lactational failure by mother?

☐ 1. Yes ☐ 2. No ☐ 99. Don't know

155) Whether mother had enough breast milk secretion?

☐ 1. Yes ☐ 2. No ☐ 99. Don't know

156) Leaking of milk from opposite breast while breast feeding the baby?

☐ 1. Yes ☐ 2. No ☐ 99. Don't know

157) How was the nipple?

☐ 1. popped up ☐ 2. retracted unilaterally ☐ 3. retracted bilaterally

158) Did the baby have cleft lip or cleft palate?

☐ 1. Yes ☐ 2. No ☐ 99. Don't know

159) Did the mother have enough breast milk secretion?

☐ 1. Yes ☐ 2. No ☐ 99. Don't know

160) Was there leaking of milk from opposite breast while breast feeding the baby?

☐ 1. Yes ☐ 2. No ☐ 99. Don't know

XIII) Haemorrhagic Disease of Newborn (Bleeding)

161) Was there bleeding from nose/mouth?

☐ 1. Yes ☐ 2. No ☐ 99. Don't know

162) Was there bleeding in urine / stool?

☐ 1. Yes ☐ 2. No ☐ 99. Don't know

163) Was there vaginal bleeding of newborn after 7 days of delivery?

☐ 1. Yes ☐ 2. No ☐ 99. Don't know

164) Was the Vomitus black?

☐ 1. Yes ☐ 2. No ☐ 99. Don't know

165) **Did a health care worker tell you the cause of death?**

☐ 1. Yes ☐ 2. No ☐ 99. Don't know

166) What did the health care worker say?

____

____

____

____

167) **Was care sought outside the home while the deceased had this illness?**

☐ 1. Yes ☐ 2. No ☐ 99. Don't know

168) **Where or from whom did you seek care?**(check all that apply)

☐ 1. Traditional healer ☐ 2. Homeopath ☐ 3. Religious leader
☐ 4. Government hospital ☐ 5. Governmental health center or clinic
☐ 6. Private hospital ☐ 7. Community-based practicitioner
☐ 8. Trained birth attendant ☐ *9. Private physician*
☐ 10. Pharmacy/drug seller/store/market ☐ 11. Other provider
☐ 12. Relative/friend (outside household)
☐ 8. Refuse to answer ☐ 99. Don't know

169) **In the month before death, how many contacts with formal health services did the baby have?**

☐ 1. _____________ number of contacts ☐ 8. Refuse to answer ☐ 99. Don't know

170) **Did (s)he receive oral rehydration salts?**

☐ 1. Yes ☐ 2. No ☐ 99. Don't know

171) **Did (s)he receive (or need) intravenous fluids treatment?**

☐ 1. Yes ☐ 2. No ☐ 99. Don't know

172) **Did (s)he receive (or need) a blood transfusion?**

☐ 1. Yes ☐ 2. No ☐ 99. Don't know

173) **Did (s)he receive (or need) intravenous fluids treatment?**

☐ 1. Yes ☐ 2. No ☐ 99. Don't know

174) **Did (s)he receive (or need) treatment/food through a tube passed through the nose?**

☐ 1. Yes ☐ 2. No ☐ 99. Don't know

175) Did (s)he receive (or need) injectable antibiotics?

☐ 1. Yes ☐ 2. No ☐ 99. Don't know

176) Did (s)he receive (or need) antiretroviral therapy (ART)?

☐ 1. Yes ☐ 2. No ☐ 99. Don't know

177) Did (s)he receive (or need) an operation for the illness?

☐ 1. Yes ☐ 2. No ☐ 99. Don't know

178) Had (s)he received immunizations?

☐ 1. Yes ☐ 2. No ☐ 99. Don't know

179) Was care sought outside the home while (s)he had this illness?

☐ 1. Yes ☐ 2. No ☐ 99. Don't know

180) Do you have the health care records that belonged to the deceased?

☐ 1. Yes ☐ 2. No ☐ 99. Don't know

181) Can I see the records?

☐ 1. Yes ☐ 2. No ☐ 99. Don't know

182) Has the deceased's (biological) mother ever been tested for "HIV"?

☐ 1. Yes ☐ 2. No ☐ 99. Don't know

183) Was the "HIV" test ever positive?

☐ 1. Yes ☐ 2. No ☐ 99. Don't know

184) Has the deceased's (biological) mother ever been told she had "AIDS" by a health worker?

☐ 1. Yes ☐ 2. No ☐ 99. Don't know

185) In the final days before death, did (s)he travel to a hospital or health facility?

☐ 1. Yes ☐ 2. No ☐ 99. Don't know

186) Did (s)he use motorized transport to get to the hospital or health facility?

☐ 1. Yes ☐ 2. No ☐ 99. Don't know

187) Were there any problems during admission to the hospital or health facility?

☐ 1. Yes ☐ 2. No ☐ 99. Don't know

188) Were there any problems with the way (s)he was treated (medical treatment, procedures, interpersonal attitudes, respect, dignity) in the hospital or health facility?

☐ 1. Yes ☐ 2. No ☐ 99. Don't know

189) Were there any problems getting medications or diagnostic tests in the hospital or health facility?

☐ 1. Yes ☐ 2. No ☐ 99. Don't know

190) Does it take more than 2 hours to get to the nearest hospital or health facility from the deceased's household?

☐ 1. Yes ☐ 2. No ☐ 99. Don't know

191) In the final days before death, were there any doubts about whether medical care was needed?

☐ 1. Yes ☐ 2. No ☐ 99. Don't know

192) In the final days before death, was traditional medicine used?

☐ 1. Yes ☐ 2. No ☐ 99. Don't know

193) In the final days before death, did anyone use a telephone or cell phone to call for help?

☐ 1. Yes ☐ 2. No ☐ 99. Don't know

XII) <u>Other Problem</u>?

194) Did the baby have other problems?

☐ 1. Yes ☐ 2. No ☐ 99. Don't know

195) Is the cause of death unknown?

☐ 1. Yes ☐ 2. No ☐ 99. Don't know

196) Was the baby treated for the illness that led to death?

☐ 1. Yes ☐ 2. No ☐ 99. Don't know

197) When ____

198) Where ____

199) By whom ____

200) What treatment ____

201) What was Diagnosis ____
