# Supplementary material for: Community—Minimal Invasive Tissue Sampling (cMITS) using a modified ambulance for ascertaining the cause of death: A novel approach piloted in a remote inaccessible rural area in India
Source: Arch Public Health. 2023 Apr 27;81:72. doi: 10.1186/s13690-023-01062-x (PMC10134564; doi:10.1186/s13690-023-01062-x)
Supplement: Supplementary file 7 — Additional file 7: Annexure 7: Verbal autopsy form of post neonatal under 5 children’s deaths. [file 13690_2023_1062_MOESM7_ESM.pdf]

MAHAN TRUST MELGHAT

VERBAL AUTOPSY OF POST NEONATAL UNDER 5 CHILDREN DEATHS
(AGE 1 MONTH TO 60 MONTHS)

Date: (dd/mm/yyyy) _____/_____/___________ **Time:**_____________

(1st May 2020 to 30th April 2021) (Dharni Block of Amaravati district)

I. GENERAL INFORMATION ABOUT FAMILY

Name of head of the household ____

Identification code of head of the household: ☐☐☐☐☐☐☐☐☐

Name of deceased____

Identification code of deceased child: ☐☐☐☐☐☐☐☐☐

Name of mother of deceased child____

Identification code of mother of deceased: ☐☐☐☐☐☐☐☐☐

Name of father of deceased child____

Identification code of father of deceased: ☐☐☐☐☐☐☐☐☐

1) **Sex of baby:** ☐ 1. Male ☐2. Female

2) **Date of Birth** (dd/mm/yyyy) : ___ ___ / ___ ___ / ____ ____ ____ ____

3) **Date of Death** (dd/mm/yyyy) : ___ ___ / ___ ___ / ____ ____ ____ ____

4) **Age at the time of Death:**
[Enter age in months up to 11 months. From 12 months enter age in years. If the exact age is unknown, enter the best estimate.]
_______ Months **OR** ________ Years

5) **Where was the place of death?** [SELECT ONE]
☐1.Home ☐2. On way to health facility ☐3.PHC/CHC/Rural Hospital
☐4.District Hospital ☐5. Private Hospital ☐6. Other place
☐ 99. Unknown

DETAILS ABOUT RESPONDENTS

6) Name of respondent:____

7) Relation with deceased: [SELECT ONE]

☐ 1.Parents (Mother/father) ☐ 2. Grandparents ☐ 3. Brother
☐ 4.Sister ☐ 5. Other relative ☐ 6.Neighbors/No relation
☐ 99.Unknown

8) Age of respondent:______years

9) Age of mother at time of baby's death:______years

10) Sex of respondent: ☐ 1. Male ☐ 2. Female

11) Education of Respondent: [SELECT ONE]

☐ 0.Illiterate/No formal education ☐ 3.SSC ☐ 99. Don't know
☐ 1.Primary (1 to 4th standard) ☐ 4.HSSC
☐ 2. Middle (5th to 9th standard) ☐ 5.Graduate & above

12) Did the respondent live with the deceased during the events that led to death?

☐ 1. Yes ☐ 2. No ☐ 99. Don't know

II. BIRTH HISTORY

13) Was the deceased a singleton or multiple birth (twin/triplet)?

☐ 1. Singleton ☐ 2. Twin ☐ 3. Triplet ☐ 99. Don't know

If singleton, -> go to Q15

14) If multiple birth, was the baby second born?

☐ 1. Yes ☐ 2. No ☐ 99. Don't know

15) Was this the first, second, or later in the birth order?

☐ 1. First child ☐ 2. Second child ☐ 3. Third or more child

16) Was this the first delivery of the mother? Whether mother was primigravida?

☐ 1. Yes ☐ 2. No ☐ 99. Don't know

17) How many births, including stillbirths, did the mother have before this baby?

Number of births/stillbirths __________ ☐ 99. Don't know

18) Did the mother receive antenatal care?

☐ 1. Yes ☐ 2. No ☐ 99. Don't know

19) Is the mother still alive?

☐ 1. Yes ☐ 2. No

If "Yes", -> go to Q22.

20) Did the mother die during or after the delivery?

☐ 1. During ☐ 2. After ☐ 99. Don't know

21) How long after the delivery did the mother die?

____ days **OR** __ __ months ☐ Don't know

22) **Was the late part of the pregnancy (defined as the last 3 months), labor, or delivery complicated by any of the following problems with the mother?** *(Read each complication and mark all that apply.)*

☐ 12. No complications
☐ 1. You (the mother) had convulsions
☐ 2. You (the mother) had high blood pressure
☐ 3. You (the mother) had severe anemia
☐ 4. You (the mother) had diabetes
☐ 5. Child delivered not head first
☐ 6. Cord delivered first
☐ 7. Cord around child's neck
☐ 10. Excessive bleeding
☐ 11. Fever
☐ 13. Blurred vision
☐ 14. Heart disease
☐ 15. Vaginal bleeding
☐ 16. Smelly vaginal discharge
☐ 17. Puffy face
☐ 18. Headache
☐ 19. Severe abdominal pain that was not labor pain
☐ 20. Pallor or shortness of breath (both present)
☐ 21. Other illnesses
☐ 99. Don't know

23) What was gestational age? *(Start counting pregnancy in **weeks** from last menstrual period*

__________weeks ☐ Don't know

24) Birth Place

☐1. in house ☐2. in hospital ☐3. on road ☐ 4. other place ☐ 99. Don't know

25) What was the size of the baby at the time of birth?

☐ 1.Very small ☐ 4. Larger than average
☐ 2. Smaller than average ☐ **3. Average** ☐ 99. Don't know

26) **What was the weight at the time of birth:**
___________gm ☐ 99. Don't know

27) **Did the baby/child have swelling or a defect on the back at time of birth?**
☐ 1. Yes ☐ 2. No ☐ 99. Don't know

28) **Did the baby/child have a very large head at time of birth?**
☐ 1. Yes ☐ 2. No ☐ 99. Don't know

29) **Did the baby/child have a very small head at time of birth?**
☐ 1. Yes ☐ 2. No ☐ 99. Don't know

30) **Before the illness that led to death was the baby/child growing normally?**
☐ 1. Yes ☐ 2. No ☐ 99. Don't know

31) **Did (s)he receive any treatment for the illness that led to death?**
☐ 1. Yes ☐ 2. No ☐ 99. Don't know

Details of the vaccinations given:

32) BCG	☐ 1. Yes	☐ 2. No	☐ 99. Don't know
33) Penta (3)	☐ 1. Yes	☐ 2. No	☐ 99. Don't know
34) Polio drops	☐ 1. Yes	☐ 2. No	☐ 99. Don't know
35) Measles	☐ 1. Yes	☐ 2. No	☐ 99. Don't know

36) **What did the respondent think the deceased died of?**

____

____

____

37) **How old was the baby/child when the fatal illness started?** *(Enter age in days up to 27 days. Enter 28 days as 1 month. From 1-11 months enter age in months. Enter 12 months as 1 year. From 1-5 years enter age in years.)*

___ ___ days **OR** ___ ___ months **OR** ___ ___ years

38) **How long did the illness last?** *(Less than 24 hours = 00 days. Enter time in days up to 27 days. Enter 28 days as 1 month. From 1-11 months enter duration of illness in months..)*

____ ____ days **OR** ____ ____ months

39) **Did the infant appear to be healthy and then just die suddenly?**

☐ 1. Yes ☐ 2. No ☐ 99. Don't know

III. ACCIDENTS AND INJURY

40) **Did s/he die from an injury or accident?**

☐ 1. Yes ☐ 2. No ☐ 99. Don't know

If no, go to next section V DIAGNOSES FROM HEALTH CARE WORKERS

41) **If yes, what kind of injury or accident?**

☐ 1. Road traffic accident
☐ 2. Falls
☐ 3. Fall of objects
☐ 4. Burns/fire
☐ 5. Drowning
☐ 6. Poisoning
☐ 7. Bite/sting
☐ 9. Natural disaster
☐ 10. Homicide/assault
☐ 11. Animal/insect
☐ 12. Firearm
☐ 13. Stabbed/cut/pierced
☐ 14. Strangled
☐ 15. Blunt force
☐ 16. Force of nature
☐ 17. Electrocution
☐ 18. Other ____
☐ 99. Don't know

42) **Was the injury accidental?**

☐ 1. Yes ☐ 2. No ☐ 99. Don't know

43) **Was the injury or accident intentionally <u>inflicted by someone else</u>?**

☐ 1. Yes ☐ 2. No ☐ 99. Don't know

44) **If (s)he died in a road accident, what was his/her role in the road traffic accident?**

☐ 1. Pedestrian
☐ 2. Passenger in car or light vehicle

- ☐ 3. Passenger in bus or heavy vehicle
- ☐ 4. Passenger on a motorcycle
- ☐ 5. Passenger on a pedal cycle
- ☐ 6. Other
- ☐ 99. Don’t know

IV. DIAGNOSES FROM HEALTH CARE WORKERS

45) **Was the Village Health Worker present at the time of death?**

☐ 1. Yes ☐ 2. No ☐ 99. Don’t know

If no, go to next section, VI RASH

46) **Was there any other diagnosis by a health professional of the following?** SELECT ANY THAT APPLY

- ☐ 1. Tuberculosis
- ☐ 2. AIDS
- ☐ 3. HIV positive test
- ☐ 4. Malaria positive test
- ☐ 5. Dengue fever
- ☐ 6. Measles
- ☐ 7. Heart disease
- ☐ 8. Diabetes
- ☐ 9. Asthma
- ☐ 10. Epilepsy
- ☐ 11. Cancer
- ☐ 12. Sickle cell disease
- ☐ 13. Kidney disease
- ☐ 14. Liver disease
- ☐ 15. Malnutrition
- ☐ 16. Other ____
- ☐ 99. Unknown

V. RASH

47) **What were the complaints of child? Explain in detail.**

(Write the information given by parents. Help them to narrate symptoms of the disease by asking supportive question)

----

48) During the illness that led to death, did s(he) have any skin rash?

☐ 1. Yes ☐ 2. No ☐ 99. Don't know

If no, go to next section, VII MALNUTRITION

49) For how many days was rash present on the body?

☐ 1. None ☐ 2. 3 days or less than 3 days ☐ 3. More than 3 days

50) Where was the rash? [SELECT ONE]

☐ 1. Face ☐ 2. Trunk or abdomen ☐ 3. Extremities ☐ 4. Everywhere

51) Did (s)he have measles rash?

☐ 1. Yes ☐ 2. No ☐ 99. Don't know

52) What did the rash look like?

☐ 1. Measles rash ☐ 2. Rash with clear fluid ☐ 3. Rash with Pus ☐ 99. Don't know

53) Was the rash was accompanied by fever?

☐ 1. Yes ☐ 2. No ☐ 99. Don't know

54) If Yes, for how many days? ____________ days

55) Was the rash accompanied by cough & cold?

☐ 1. Yes ☐ 2. No ☐ 99. Don't know

56) Were the eyes congested and/or red?

☐ 1. Yes ☐ 2. No ☐ 99. Don't know

57) Did the rash change from red to black- brown then faded before disappearance and later on vanished?

☐ 1. Yes ☐ 2. No ☐ 99. Don't know

58) During the illness that led to death, did the baby's skin flake off in patches?

☐ 1. Yes ☐ 2. No ☐ 99. Don't know

59) Did (s)he have mouth sores or white patches in the mouth or on the tongue?

☐ 1. Yes ☐ 2. No ☐ 99. Don't know

60) For how long did (s)he have mouth sores or white patches in the mouth or the tongue?

____hours **OR** __ __ days **OR** ____ weeks **OR** ______ months

61) Whether there were blisters? (like Chickenpox)

☐ 1. Yes ☐ 2. No ☐ 99. Don't know

62) During the illness that led to death did s(he) have areas of skin with redness and swelling?

☐ 1. Yes ☐ 2. No ☐ 99. Don't know

63) During the illness that led to death did s(he) have a whitish rash outside the mouth or on the tongue?

☐ 1. Yes ☐ 2. No ☐ 99. Don't know

VI. MALNUTRITION

64) Was the growth of the baby normal especially during last 4 months?

☐ 1. Yes ☐ 2. No ☐ 99. Don't know

65) Did the child have noticeable weight loss?

☐ 1. Yes ☐ 2. No ☐ 99. Don't know

66) For how long before death did (s)he have the weight loss?

_____hours OR __ __ days OR _____ weeks OR _______ months

67) Was your baby malnourished?

☐ 1. Yes ☐ 2. No ☐ 99. Don't know

68) Was the child severely thin or wasted?

☐ 1. Yes ☐ 2. No ☐ 99. Don't know

69) Did (s)he have any swelling?

☐ 1. Yes ☐ 2. No ☐ 99. Don't know

If no, go to next section, VIII BREAST FEEDING

70) For how long did the (s)he have the swelling? SELECT ONE

_____hours OR __ __ days OR _____ weeks OR _______ months

71) If yes, where was the swelling:

☐ 1. Feet/lower leg
☐ 2. Lower back
☐ 3. Face
☐ 4. Whole body
☐ 5. Other place
☐ 99. Don't know

72) How many days did the swelling last? ________ Days ☐ 99. Don't know

VII. BREAST FEEDING

73) When was the baby first breastfed?

☐ 1. Immediately/within 1hr of birth
☐ 2. Same day of birth
☐ 3. Second day/later
☐ 4. Never breastfed
☐ 99. Don't know

74) Did the baby receive anything other than breast milk during 6mths of life after birth?

☐ 1. Yes ☐ 2. No ☐ 99. Don't know

75) At what age (months) child was fed supplementary top feed or bottle Milk?

☐ 1. Within first 3 months ☐ 2. Fourth month ☐ 3. Fifth month

76) What was the age of complementary/weaning food?

☐ 1. After 9 months ☐ 2. After 7 months ☐ 3. After 8 months

77) During the illness that led to death, was the child breastfeeding?

☐ 1. Yes ☐ 2. No ☐ 99. Don't know

78) How was the appetite of the baby in last month before dying?

☐ 1. Good ☐ 2. Less ☐ 99. don't know

VIII. APPEARANCE

79) How was health of baby in last 3 months?

☐ 1. Good (not sick/ill) ☐ 2. Ill/sick ☐ 99. Don't know

80) Was the child playful in last month?

☐ 1. Good ☐ 2. (less/No) ☐ 99. Don't know

81) Was the baby suffering from night blindness?

☐ 1. Yes ☐ 2. No ☐ 99. Don't know

82) Did the baby's hair change in color to a reddish or yellowish color?

☐ 1. Yes ☐ 2. No ☐ 99. Don't know

83) For how long did (s)he have reddish/yellowish hair?

_____hours OR __ __ days OR _____ weeks OR _______ months

84) Did the baby have a protruding belly?

☐ 1. Yes ☐ 2. No ☐ 99. Don't know

85) During the illness that led to death, did the baby suffer from "lack of blood" or "pallor" or have pale palms, eyes, or nail beds?

☐ 1. Yes ☐ 2. No ☐ 99. Don't know

86) For how long did (s)he look pale or have pale palms, eyes, or nail beds?

_____hours OR __ __ days OR _____ weeks OR _______ months

87) During the illness that led to death, did he/she have swelling in the armpits?

☐ 1. Yes ☐ 2. No ☐ 99. Don't know

88) During the illness that led to death, did he/she bleed from anywhere?

☐ 1. Yes ☐ 2. No ☐ 99. Don't know

89) Did he/she bleed from nose, mouth or anus?

☐ 1. Yes ☐ 2. No ☐ 99. Don't know

90) During the illness that led to death, did he/she have areas of the skin that turned black?

☐ 1. Yes ☐ 2. No ☐ 99. Don't know

IX. COUGH

91) Was the child suffering from cough?

☐ 1. Yes ☐ 2. No ☐ 99. Don't know

If no, go to next section, XI. BREATHING PROBLEMS

92) Was the cough very severe?

☐ 1. Yes ☐ 2. No ☐ 99. Don't know

93) what was the duration of the cough?_ ______ days

94) Whether the cough was in long bouts?

☐ 1. Yes ☐ 2. No ☐ 99. Don't know

95) Whether the face of child becomes suffused -red or cyanosed (blue) during bouts of cough?

☐ 1. Yes ☐ 2. No ☐ 99. Don't know

96) Whether there was whooping sound during cough?

☐ 1. Yes ☐ 2. No ☐ 99. Don't know

97) **Was there no sucking or feeding due to frequent bouts of cough/vomiting?**

☐ 1. Yes ☐ 2. No ☐ 99. Don't know

98) **Whether there was whooping cough/ dog cough?**

☐ 1. Yes ☐ 2. No ☐ 99. Don't know

99) **Was the child in contact with another child suffering from dry cough, whooping cough? Was there epidemic of whooping cough in village?**

☐ 1. Yes ☐ 2. No ☐ 99. Don't know

100) **Did the child cough up blood?**

☐ 1. Yes ☐ 2. No ☐ 99. Don't know

101) **Did the child vomit after (s)he coughed?**

☐ 1. Yes ☐ 2. No ☐ 99. Don't know

X. BREATHING PROBLEMS

102) **Was the child suffering from breathlessness/fast/laboured breathing/ grunting?**

☐ 1. Yes ☐ 2. No ☐ 99. Don't know

103) **During the illness that led to death, did the child have difficulty breathing?**

☐ 1. Yes ☐ 2. No ☐ 99. Don't know

If no, skip Q104 & Q105

104) **For how many days did the difficult breathing last?**

__ __ days (Enter 99 if unknown) ☐ Don't know

105) **Was the difficulty continuous or on and off?**

☐ 1. Continuous ☐ 2. On and off ☐ 99. Don't know

106) **During the illness that led to death, did the baby have fast breathing?**

☐ 1. Yes ☐ 2. No ☐ 99. Don't know

107) **If yes, for how many days did the fast breathing last?**

__ __ days (Enter 99 if unknown) ☐ Don't know

108) **During the illness that led to death did his/her breathing sound like any of the following?**

☐ 1. Stridor ☐ 2. Grunting ☐ 3. Wheezing ☐ 4. None ☐9. Don't know

109) **Did the child have chest indrawing?**

☐ 1. Yes ☐ 2. No ☐ 99. Don't know

110) **If yes, for how long did (s)he have chest indrawing?**

____hours **OR** __ __ days **OR** ____ weeks **OR** ______ months

111) **Did (s)he have flaring of the nostrils?**

☐ 1. Yes ☐ 2. No ☐ 99. Don't know

112) **Did (s)he have chest pain?**

☐ 1. Yes ☐ 2. No ☐ 99. Don't know

113) **If yes, for how many days did (s)he have chest pain?**

__ __ days

114) **Did the child have night sweats?**

☐ 1. Yes ☐ 2. No ☐ 99. Don't know

115) **Was the child irritable?**

☐ 1. Yes ☐ 2. No ☐ 99. Don't know

XI. DIARRHEA

116) **Was the baby suffering from loose motion? (diarrhea?)**

☐ 1. Yes ☐ 2. No ☐ 99. Don't know

If no, go to next section, XIII. VOMITING

117) **How many times in 24 hours?** __________

118) **Did the child have watery stools?**

☐ 1. Yes ☐ 2. No ☐ 99. Don't know

119) **Did the stool contain blood or mucus?**

☐ 1. Yes ☐ 2. No ☐ 99. Don't know

120) **How many days after birth did the baby have loose motion?**__________days

121) **If Yes, how many times a day in last 3 months?** __________times a day

122) **How many days, during last 3 months?** __________days

123) **Was the baby suffering from loose motion continuously for 15 days or more?**

☐ 1. Yes ☐ 2. No ☐ 99. Don't know

124) **During the illness that led to death, did he/she have more frequent loose or liquid stools than usual?**

☐ 1. Yes ☐ 2. No ☐ 99. Don't know

125) Did the frequent loose or liquid stools continue until death?
☐ 1. Yes ☐ 2. No ☐ 99. Don't know

126) Was there a period of a day or longer during which (s)he did not pass any stool?
☐ 1. Yes ☐ 2. No ☐ 99. Don't know

127) Was breast feeding or fluids feeding continued during loose motion? (less or more feeding?)
☐ 1. More ☐ 2. Less/No ☐ 99. Don't know

XII. VOMITING

128) Whether child was suffering from Vomiting?
☐ 1. Yes ☐ 2. No ☐ 99. Don't know

If no, go to next section, XIV. ABDOMINAL PROBLEM

129) Did (s)he vomit the week preceding death?
☐ 1. Yes ☐ 2. No ☐ 99. Don't know

130) Was there blood in the vomit?
☐ 1. Yes ☐ 2. No ☐ 99. Don't know

131) When the vomiting was most severe, how many times did (s)he vomit in a day?
________days

132) How many days after birth did vomiting start?
________days

133) Was the vomit black?
☐ 1. Yes ☐ 2. No ☐ 99. Don't know

XIII. ABDOMINAL PROBLEM

134) Did (s)he have any belly (abdominal) problem?
☐ 1. Yes ☐ 2. No ☐ 99. Don't know

135) Did (s)he have any belly (abdominal) pain?
☐ 1. Yes ☐ 2. No ☐ 99. Don't know

If no, go to next section, XV.PROTRUDING BELLY

136) For how long did (s)he have belly (abdominal) pain?
_____hours OR __ __ days OR _____ weeks OR _______ months

137) Was the belly (abdominal) pain severe?
☐ 1. Yes ☐ 2. No ☐ 99. Don't know

138) Was the pain in the upper of lower belly (abdomen)?
☐ 1. Upper ☐ 2. Lower ☐ 2. Upper and lower ☐ 99. Don't know

XIV. PROTRUDING BELLY

139) Did s(he) have a more than usually protruding belly (abdomen)?
☐ 1. Yes ☐ 2. No ☐ 99. Don't know

If no, go to next section, XVI. BELLY MASS

140) For how long did (s)he have protruding belly (abdomen)/distension?
_____hours OR __ __ days OR _____ weeks OR _______ months

141) Did the protruding belly develop rapidly within days or gradually over months?
☐ 1. Rapidly over days ☐ 2. Gradually over months ☐ 99. Don't know

XV. BELLY MASS

142) Did s(he) have any mass in the belly (abdomen)?
☐ 1. Yes ☐ 2. No ☐ 99. Don't know

If no, go to next section, XVII. HYDRATION

143) If yes, for how many months before death did (s)he have the mass in the abdomen?
_____hours OR __ __ days OR _____ weeks OR _______ months

XVI. HYDRATION

144) How was the thirst?
☐ 1. Increased ☐ 2. Decreased ☐ 3. Normal ☐ 99. Don't know

145) Did the child have sunken eyes?
☐ 1. Yes ☐ 2. No ☐ 99. Don't know

146) **For how long did (s)he have sunken eyes?**

_____hours **OR** __ __ days **OR** _____ weeks **OR** _______ months

147) **How was the (anterior) fontanelle?**

☐ 1. Plain/ normal ☐ 2. Depressed inside ☐ 3. bulged/raised ☐ 99. Don't know

148) **If s(he) has bulging fontanelle, for how many days before death?**

________days

149) **Was there any change in the amount of urine (s)he passed daily?**

☐ 1. Yes ☐ 2. No ☐ 99. Don't know

150) **For how long did (s)he have the change in the amount of urine (s)he passed daily?**

_____hours **OR** __ __ days **OR** _____ weeks **OR** _______ months

151) **How much urine did (s)he pass?**

☐ 1. Too much ☐ 2. Too little ☐ 3. No urine at all ☐ 99. Don't know

152) **What was the color of urine?**

☐ 1. Watery/Normal ☐ 2. Yellow ☐ 99. Don't know

153) **Did (s)he have any urine problems such as frequent urination or blood in urine?**

☐ 1. Yes ☐ 2. No ☐ 99. Don't know

154) **Did (s)he stop urinating?**

☐ 1. Yes ☐ 2. No ☐ 99. Don't know

155) **Did (s)he go to urinate more often than usual?**

☐ 1. Yes ☐ 2. No ☐ 99. Don't know

156) **Did (s)he ever pass blood in the urine during the final illness?**

☐ 1. Yes ☐ 2. No ☐ 99. Don't know

XVII. SKIN

157) **Did (s)he have sores or ulcers anywhere on the body?**

☐ 1. Yes ☐ 2. No ☐ 99. Don't know

If no, go to next section, XIX. DROWSY OR UNCONSCIOUS

158) **Did the sores have clear fluid or pus?**

☐ 1. Yes ☐ 2. No ☐ 99. Don't know

159) **Did (s)he have an ulcer (pit) on the foot?**

☐ 1. Yes ☐ 2. No ☐ 99. Don't know

160) **Did the ulcer on the foot ooze pus?**

☐ 1. Yes ☐ 2. No ☐ 99. Don't know

XVIII. DROWSY OR UNCONSCIOUS

161) **Whether child was drowsy or unconscious?**

☐ 1. Yes ☐ 2. No ☐ 99. Don't know

If no, go to next section, XX. CONVULSIONS

162) **How long before death did unconsciousness start?**

☐ 1. Less than 6 hours ☐ 2. 6-23 hours ☐ 3. 24 hours or more

☐ 9. Don't know

163) **For how long was (s)he unconscious?**

_____hours OR __ __ days OR _____ weeks OR _______ months

164) **Did the unconsciousness start suddenly, quickly (at least within a single day)?**

☐ 1. Yes ☐ 2. No ☐ 99. Don't know

165) **Did the unconsciousness continue until death?**

☐ 1. Yes ☐ 2. No ☐ 99. Don't know

XIX. CONVULSIONS

166) **Did the child have convulsions?**

☐ 1. Yes ☐ 2. No ☐ 99. Don't know

If no, go to next section, XXI. NEURO

167) **Did the convulsions start more than 24 hours after birth?**

☐ 1. Yes ☐ 2. No ☐ 99. Don't know

168) **Did (s)he experience any generalized convulsions during the illness that led to death?**

☐ 1. Yes ☐ 2. No ☐ 99. Don't know

169) **For how many minutes did the convulsions last?**

_________minutes

170) **Did (s)he become unconscious immediately after the convulsions?**

☐ 1. Yes ☐ 2. No ☐ 99. Don't know

171) For how long did (s)he have convulsions?

____hours OR __ __ days OR ____ weeks OR ______ months

XX. NEURO

172) Whether there was any ear discharge?

☐ 1. Yes ☐ 2. No ☐ 99. Don't know

173) Was neck stiffness present?

☐ 1. Yes ☐ 2. No ☐ 99. Don't know

174) IF YES, For how many days before death did (s)he have stiff neck?_ __________ days

175) Did (s)he have a painful neck during the illness that led to death?

☐ 1. Yes ☐ 2. No ☐ 99. Don't know

176) Whether child was suffering from continuous headache?

☐ 1. Yes ☐ 2. No ☐ 99. Don't know

177) If yes, for how long did (s)he have headache?

____hours OR __ __ days OR ____ weeks OR ______ months

178) Did the child have stiffness of the whole body or was unable to open the mouth?

☐ 1. Yes ☐ 2. No ☐ 99. Don't know

179) Was the body of baby stiff, with the back arched backwards like a bow?

☐ 1. Yes ☐ 2. No ☐ 99. Don't know

XXI. PARALYSIS

180) Was the child in any way paralysed?

☐ 1. Yes ☐ 2. No ☐ 99. Don't know

If no, go to next section, XXIII.OTHER QUESTIONS

181) Did s(he) have paralysis of only one side of the body?

☐ 1. Yes ☐ 2. No ☐ 99. Don't know

182) Which were the limbs or body parts paralysed?

☐ 1. Right side ☐ 2. Left side ☐ 3. Lower part of body ☐ 4. Upper part of body
☐ 5. One leg only ☐ 6. One arm only ☐ 8. Whole body ☐ 9. Other_______________

183) **Did (s)he have paralysis of the lower limb?**
☐ 1. Yes ☐ 2. No ☐ 99. Don't know

184) **How long did (s)he have paralysis of the lower limbs?**
____hours **OR** __ __ days **OR** ____ weeks **OR** ______ months

185) **Did the paralysis of the lower limbs start suddenly, quickly within a single day, or slowly over many days?**
☐ 1. Suddenly ☐ 2. Fast (in a day) ☐ 3. Slowly (many days) ☐ 99. Don't know

XXII. OTHER QUESTIONS

186) **Did s(he) have difficulty swallowing?**
☐ 1. Yes ☐ 2. No ☐ 99. Don't know

187) **For how many days did the child have difficulty swallowing?** _____________ Days

188) **Did (s)he have pain upon swallowing?**
☐ 1. Yes ☐ 2. No ☐ 99. Don't know

189) **Whether child had continuous vomiting?**
☐ 1. Yes ☐ 2. No ☐ 99. Don't know

190) **Did s(he) drink a lot more water than usual?**
☐ 1. Yes ☐ 2. No ☐ 99. Don't know

191) **Was the baby able to suckle in a normal way during the first day of life?**
☐ 1. Yes ☐ 2. No ☐ 99. Don't know

192) **Did the baby stop sucking milk?**
☐ 1. Yes ☐ 2. No ☐ 99. Don't know

193) **How many days after birth did the baby stop suckling?** _____________ Days

194) **Did the baby ever suckle in a normal way?**
☐ 1. Yes ☐ 2. No ☐ 99. Don't know

195) **Whether any family member was suffering from TB?**
☐ 1. Yes ☐ 2. No ☐ 99. Don't know

196) **Was the child sucking breast milk/drinking fluids?**
☐ 1. Yes ☐ 2. No ☐ 99. Don't know

197) **Whether child had clenching of teeth? Was it associated with frothing from mouth?**
☐ 1. Yes ☐ 2. No ☐ 99. Don't know

198) **During the illness that led to death, did he/she have yellow discoloration of the eyes?**
☐ 1. Yes ☐ 2. No ☐ 99. Don't know

199) **For how long did the child have the yellow discoloration?**
____hours **OR** __ __ days **OR** ____ weeks **OR** ______ months

200) **Was the belly (abdominal) pain severe?**
☐ 1. Yes ☐ 2. No ☐ 99. Don't know

XXIII. LUMPS

201) **Did the child have any lumps?**
☐ 1. Yes ☐ 2. No ☐ 99. Don't know

If no, go to next section, XXV. FEVER

202) **For how long did (s)he have the lumps?**
____hours **OR** __ __ days **OR** ____ weeks **OR** ______ months

203) **Were the lumps on:**
☐ 1. The neck?
☐ 2. The armpit?
☐ 3. The groin?
☐ 4. Any other place? ______________________

XXIV. FEVER

204) **Was the child suffering from fever?**
☐ 1. Yes ☐ 2. No ☐ 99. Don't know

If no, go to next section, XXVI. TREATMENT HISTORY ,

205) **Was fever accompanied by :**
☐ 1. loose motion
☐ 2. breathlessness
☐ 3. unconsciousness

☐ 4. Convulsions

206) **How many days did the fever last?**
☐ 1. Less than 24 hours ☐ __ __ days ☐ Don't know

207) **Did the fever continue until death?**
☐ 1. Yes ☐ 2. No ☐ 99. Don't know

208) **How severe was the fever?**
☐ 1. Mild ☐ 2. Moderate ☐ 3. Severe ☐ 99. Don't know

209) **What was the pattern of the fever?**
☐ 1. Continuous ☐ 2. On and off ☐ 3. Only at night ☐ 99.Don't know

210) **Did (s)he have chills/rigor?**
☐ 1. Yes ☐ 2. No ☐ 99. Don't know

XXV. TREATMENT HISTORY

211) **Was there any other complains? If yes then describe the nature?**

……
……
……
……
……
……
……
……

212) **Did a health care worker tell you the cause of death?**
☐ 1. Yes ☐ 2. No ☐ 99. Don't know

213) **What did the health care worker say?**

____
____
____

214) **Was care sought outside the home while the deceased had this illness?**
☐ 1. Yes ☐ 2. No ☐ 99. Don't know

If no, go to next section, Q204

215) **Where or from whom did you seek care?**

- ☐ 1. Traditional healer
- ☐ 2. Homeopath /Ayurveda doctor
- ☐ 3. Religious leader
- ☐ 4. Government hospital
- ☐ 5. Governmental health center or clinic
- ☐ 6. Private hospital
- ☐ 7. Community-based practitioner
- ☐ 8. Trained birth attendant / VHW
- ☐ 9. Private physician
- ☐ 10. Pharmacy/drug seller/store/market
- ☐ 11. Other provider
- ☐ 12. Relative/friend (outside household)
- ☐ 99. Don't know

216) **Did (s)he receive oral rehydration salts?**

☐ 1. Yes ☐ 2. No ☐ 99. Don't know

217) **Did (s)he receive (or need) intravenous fluids treatment?**

☐ 1. Yes ☐ 2. No ☐ 99. Don't know

218) **Did (s)he receive (or need) a blood transfusion?**

☐ 1. Yes ☐ 2. No ☐ 99. Don't know

219) **Did (s)he receive (or need) treatment/food through a tube passed through the nose?**

☐ 1. Yes ☐ 2. No ☐ 99. Don't know

220) **Did (s)he receive (or need) injectable antibiotics?**

☐ 1. Yes ☐ 2. No ☐ 99. Don't know

221) **Did (s)he receive (or need) antiretroviral therapy (ART)?**

☐ 1. Yes ☐ 2. No ☐ 99. Don't know

222) **Did (s)he receive (or need) an operation for the illness?**

☐ 1. Yes ☐ 2. No ☐ 99. Don't know

If no, go to next section XXVII. HEALTH CARE RECORDS

223) **Did (s)he have the operation within 1 month before death?**

☐ 1. Yes ☐ 2. No ☐ 99. Don't know

224) **How long before death did (s)he have the operation?**

☐ 1. Days _____________ ☐ 99. Don't know

225) **On what part of the body was the operation?**

☐ 1. Abdomen ☐ 2. Chest ☐ 3. Head ☐ 4. Other ____________ ☐ 99. Don't know

226) Was (s)he discharged from hospital very ill?

☐ Yes ☐ No ☐ Don't know

227) In the month before death, how many contacts with formal health services did (s)he have?

Number of contacts ____________ ☐ Don't know

228) If contacts with formal health services then what? ______________________________

229) by Whom ……………………………………………………………………………………..

230) Where ……………………………………………………………………………………..

231) What was nature of treatment ………………………………………………………………………………

232) Duration of treatment ………………………………………………………………………………

233) Cause of death as per person who has treated the child………………………………………..

XXVI. HEALTH CARE RECORDS

234) Do you have the health care records that belonged to the deceased?

☐ 1. Yes ☐ 2. No ☐ 99. Don't know

235) Can I see the records?

☐ 1. Yes ☐ 2. No ☐ 99. Don't know

236) Do you have the death certificate?

☐ 1. Yes ☐ 2. No ☐ 99. Don't know

237) Can I see the death certificate?

☐ Y1. Yes ☐ 2. No ☐ 99. Don't know

238) In the final days before death, did (s)he travel to a hospital or health facility?

☐ 1. Yes ☐ 2. No ☐ 99. Don't know

239) Did (s)he use motorized transport to get to the hospital or health facility?

☐ 1. Yes ☐ 2. No ☐ 99. Don't know

240) Were there any problems during admission to the hospital or health facility?

☐ 1. Yes ☐ 2. No ☐ 99. Don't know

241) Were there any problems with the way (s)he was treated (medical treatment, procedures, interpersonal attitudes, respect, dignity) in the hospital or health facility?

☐ 1. Yes ☐ 2. No ☐ 99. Don't know

242) Were there any problems getting medications or diagnostic tests in the hospital or health facility?

☐ 1. Yes ☐ 2. No ☐ 99. Don't know

243) Does it take more than 2 hours to get to the nearest hospital or health facility from the deceased's household?

☐ 1. Yes ☐ 2. No ☐ 99. Don't know

244) In the final days before death, were there any doubts about whether medical care was needed?

☐ 1. Yes ☐ 2. No ☐ 99. Don't know

245) In the final days before death, was traditional medicine used?

☐ 1. Yes ☐ 2. No ☐ 99. Don't know

246) In the final days before death, did anyone use a telephone or cell phone to call for help?

☐ 1. Yes ☐ 2. No ☐ 99. Don't know

247) Has the deceased's (biological) mother ever been tested for "HIV"?

☐ 1. Yes ☐ 2. No ☐ 99. Don't know

248) Was the "HIV" test ever positive?

☐ 1. Yes ☐ 2. No ☐ 99. Don't know

249) Has the deceased's (biological) mother ever been told she had "AIDS" by a health worker?

☐ 1. Yes ☐ 2. No ☐ 99. Don't know
