# Supplementary material for: Community—Minimal Invasive Tissue Sampling (cMITS) using a modified ambulance for ascertaining the cause of death: A novel approach piloted in a remote inaccessible rural area in India
Source: Arch Public Health. 2023 Apr 27;81:72. doi: 10.1186/s13690-023-01062-x (PMC10134564; doi:10.1186/s13690-023-01062-x)
Supplement: Supplementary file 8 — Additional file 8: Annexure 8: Verbal autopsy form for death of people in age group of 16-60 years. [file 13690_2023_1062_MOESM8_ESM.pdf]

महान ट्रस्ट, कर्मग्राम उतावली, मेलघाट
(Verbal Autopsy Form)
१६ – ६० वर्षे पर्यंत मृत्यु सर्वेक्षण (कारणमिमांसा)

दिनांक: पावती क्र.

(RSV burden of disease study)

गांव क्रमांक घर क्रमांक परिवार क्रमांक व्यक्ती क्रमांक

पहचान क्रमांक ( )( )( ) – ( )( )( ) – ( )( )( )( )( )( ) – ( )( )

१) मृतक का पूरा नाम : ___________________ पिता का नाम ___________________ जाटो _______________

२) जन्मतारीख (पता है तो): ( ) ( )/ ( ) ( )/ ( )( )( )( ) (दिन /महिना/साल) ३) लिंग: पुरुष ( )स्त्री ( )

४) गांव का नाम (हमेशा रहनेका) : __________________

५) मरने कि तारीख : ( ) ( )/ ( ) ( )/ ( )( )( )( ) (दिन /महिना/साल) ६)मृत्यू के समय उम्र: ( )( )( )

७) जहाँ मृत्यू हुआ वह गाँव का नाम : __________________

८) मुलाखत कि तारीख: ( ) ( )/ ( ) ( )/ ( )( )( )( ) (दिन /महिना/साल)

९) मरने का कारण :-

१०) पर्यवेक्षक का नाम:

१)- कैसा क्या मर गया? क्या तकलीफ हुई थी यह सविस्तर बताइये| बताया हुआ वर्णन लिखिए| बाहरकी बिमारी है यह कारण न बताते हुए क्या तकलीफ थी, यह समजाने में मदत किजीये|

----

१०) क्या महिला अरोग्यदूत उपस्थित थी? हाँ (----------------) नही (---------------)

११) जानकारी देनेवाले का नाम:- -------------------------------- मृतक के साथ रिश्ता: ----------------------------

सुचना:-

१) कोई भी सवाल के सामने (----------) जगह रहने पर, वह तकलीफ मरने के कितने दिन पहले से थी,(तकलीफ एक दिन से कम समय रहने पर) कितने घंटो से थी यह लिखिये|

२) बड़े कठिन सवालो का भाग करके दो –तिन सवालों द्वारा जानकारी पुछीये|

I) क्या मरिज नीचे लिखे किसी कारण से मर गया|

१) अपघात	( )	२) पानी में डूबकर	( )	३) चोट	( )
४) गिरने से	( )	५) सांप काटने से	( )	६) जहर खाने से	( )
५) जंगली जानवरों ने मारा	( )	८) खून हुआ	( )	९) खुद कुशी कीयी	( )

C. हाँ. (-----------------------) नही ( ) पता नही ( )

अपघात वैगरे

बहुतेक = 1 C -----------------

II) साँस कि बिमारी

1. क्या मरिज को खाँसी थी?
हाँ. (----------------------) नही ( ) पता नही ( )

2. क्या मरिज को दम लगता था? साँस फुलती थी? क्या मरिज को साँस लेने में कोई दिक्कत थी?
हाँ. (----------------------) नही ( ) पता नही ( )
यदी दोनों नहीं तो III पर जाइये|
एक या दोनों हाँ होंगे तो सवाल ३ से ७ पुछिये|

3. खाँसी कब से थी? (----------------------)
E (२४ घंटों से ज्यादा)

4. क्या खाँसी में लाल (गंजने जैसा) फेफड़ा जाता था?
C हाँ. (----------------------) नही ( ) पता नही ( )

5. दम/साँस फुलना कब से था? (----------------------)
C (६ घंटो से ज्यादा)

6. क्या मरिज बुखार था?
S हाँ. (----------------------) नही ( ) पता नही ( )

7. क्या सीना/पासलीया अंदर खिची जाती थी?
C हाँ. (----------------------) नही ( ) पता नही ( )

8. क्या सीने में दर्द था?
C हाँ. (----------------------) नही ( ) पता नही ( )
क्या साँस लेने से बढ़ता था?
C हाँ. (--------------------) नही ( ) पता नही ( ) (दोनों में से कितने भी S होने पर 1S)

न्युमोनिया
संभव = 1E + 1S
बहूदा =1E + 1C + 2 S अथवा 1E + 1S + 2C

सवाल 3 से 6 में तकलीफ (खाँसी या बुखार) 15 दिन से ज्यादा समय तक होंगी तो सवाल 9 से २५ तक पुछीये| यदी नहीं होंगी तो III पर जाइये|

9. क्या खाँसी कब से थी? (----------------------)
E हाँ (यदी 15 दिनसे ज्यादा होगा, तो 1E) नहीं ( ) पता नहीं ( )

10. क्या खाँसी में खून जाता था?
C हाँ (----------------------) नही ( ) पता नही ( )

11. क्या मरिज दिन ब दिन सुखता जा रहा था? और वजन घटते जा रहा था? (गत 15 दिन से ज्यादा समय से सतत)
E हाँ (----------------------) नही ( ) पता नही ( )

12. आखिर 15 दिन में मरिज की तबीयत कैसे थी?
E बिमारी थी? (----------------------) अच्छी थी ( ) पता नही ( )

13. क्या मरिज को एक माह से बहोत कमजोरी आयी थी?
S हाँ (----------------------) नही ( ) पता नही ( )

14. मरने के पहले 30 दिन के अंदर तक मरिज का खाना कैसे था?
S कम (----------------------) अच्छा ( ) पता नही ( )

15. क्या मरिज को T.B. (क्षयरोग/सुखी) की बिमारी थी?
S हाँ (----------------------) नहीं ( ) पता नही ( )

16. क्या T.B (क्षयरोग) के मरिज के साथ इस मरीज का संपर्क (साथ) आया था?
S हाँ (----------------------) नहीं ( ) पता नही ( )

17. क्या मरीज को T.B. की लस (बि.सी.जी.) लगाई थी?
हाँ (----------------------) S नहीं ( ) पता नही ( )

18. क्या मरीज के गले/ काख में 15 दिन से ज्यादा समय तक गठान थी?
C हाँ (----------------------) नहीं ( ) पता नही ( )

19. क्या मरीज को 15 दिनसे ज्यादा समय तक गर्दन| सिर में दर्द और उलटिया थी?
S हाँ (----------------------) नहीं ( ) पता नही ( )

20. क्या मरीज के आवाज में बदल, घोगरेपन तथा निगलने में तकलीफ होती थी?
S हाँ (----------------------) नहीं ( ) पता नही ( )

21. क्या दम/साँस फुलना/ पसलिया अंदर खिची जाना था? और कबसे?
C हाँ (----------------------) नहीं ( ) पता नही ( )

T.B.
संभवता = E1+ 1S +1C/ 2E + 3S
बहूदा = 3E + 2C अथवा 2E + 1C +5S
अथवा 2E + 2C +2S

(सुचना:15 दिनसे १ माहके ऊपर खाँसी फुलना रहनेपर T. B. की संभावना ज्यादा है)

22. क्या मरीज को बहोत दिन से दम लगता था? या साँस फुलती थी?
(जिसके वजह से वह काम नहीं कर सकता था)
E हाँ (----------------------) नहीं ( ) पता नही ( )

23. क्या मरीज को बहोत दिन से खाँसी थी?
E हाँ (----------------------) नहीं ( ) पता नही ( )

24. क्या मरीज दम के वजह से दिन रात बैठा रहता था? सो नहीं सकता था?
C हाँ (----------------------) नहीं ( ) पता नही ( )

25. क्या मरीज को लगातार तीन साल से प्रत्येक वर्ष हररोज दो माह तक खाँसी में सफ़ेद चिकट बलगम फेफड़ा आता था?
C हाँ (----------------------) नहीं ( ) पता नही ( )

क्या वह खाँसी थंडी के दिनों बढती थी?
S हाँ (----------------------) नहीं ( ) पता नही ( )

26. क्या मरीज के ओठ, पैर, नीलें हुयें थे?
C हाँ (----------------------) नहीं ( ) पता नही ( )

27. क्या मरीज को बार-बार दमा होता था?
S हाँ (----------------------) नहीं ( ) पता नही ( )

28. क्या मरीज को दिन से बिडी/चिलम/सिगारेट पिने की आदत थी?
C हाँ (----------------------) नहीं ( ) पता नही ( )

COAD
संभवता = E1+ 1S / 1E + 1C
बहूदा = 2E + 2C / 1E +1C +2S / 2E +1C+2S

(सवाल 22 से 28 तक)

III) हृदयरोग (IDH)

1. क्या मरीज को सीने में (बाया हिस्सा/ बिचमें) दर्द हुआ था|
(वजन रखा जैसे लगता था?)
E हाँ (----------------------) नहीं ( ) पता नही ( )

2. क्या मरीज के सीनेमें घुटन जैसे महसुस हुआ? या घबराहट हो रही थी? जो कम करने से बढती थी? और आराम करने से कम होती थी?
C हाँ (----------------------) नहीं ( ) पता नही ( )

3. क्या सीने का दर्द बाये हाथ में गया था?
C हाँ (----------------------) नहीं ( ) पता नही ( )

4. क्या मरीज को बहोत पसीना आता था?
S हाँ (----------------------) नहीं ( ) पता नही ( )

5. क्या मरीज को चलने से कम करने से या सीडी चढ़ने से दम लगता था?
S हाँ (----------------------) नहीं ( ) पता नही ( )

6. क्या मरीज चक्कर आके गिर गया था?
S हाँ (----------------------) नहीं ( ) पता नही ( )

7. क्या मरीज की दिल की धड़कन बढ़ती थी?
S हाँ (----------------------) नहीं ( ) पता नही ( )

8. क्या मरीज को उलटी हुई थी?
S हाँ (----------------------) नहीं ( ) पता नही ( )

9. क्या मरीज को तंबाखू, बिडी,चिलम, सिगारेट या गांजा पिने की आदत थी?
S हाँ (----------------------) नहीं ( ) पता नही ( )

10. क्या मरीज को पैर से उपर तक सुजन (उलटी सुजन) आई थी?
S हाँ (----------------------) नहीं ( ) पता नही ( )

हृदयरोग (IDH)
संभवता = 1E+ 1S / 1E + 1C
बहूदा = 1E + 1C + 2S / या 2C अथवा 1E +2C

IV) पतली दस्त (दस्तकी बीमारी)

1. क्या मरीज को पतली तट्टी होती थी? हाँ (---------------) नहीं ( ) पता नही ( )
यदी हाँ, तो एक दिन में और रात में ज्यादा से ज्यादा कितने बार? तिन बार या उससे कम होने पर V पर जाइये|
E एक दिन में और रात तीन से ज्यादा बार पतले दस्त होने पर, सवाल 2. पुछियें|

2. क्या तट्टी में खुन या बहोत ऑव गिरती थी?
C हाँ (----------------------) नहीं ( ) पता नही ( )

डिसेंट्री
बहुतेक = 1E + 1C
यदी हाँ तो VI पर जाइये
यदी पता नहीं तो सवाल 3 पछिये|

3. कितने दिन से पतली दस्त शुरू थी? (--------------------------------)
C १५ दिन से ज्यादा दिन तक

लंबी हगवन|पतली दस्त
बहुतेक = 1E + 1C
सिर्फ सवाल 1 और 3 के लिये।

१५ दिन से कम समय होगा तो सवाल ४ से ११ पूछिये|

4. क्या दस्त पाणी जैसे पतली (ढाळ) होती थी?
C हाँ (----------------------) नहीं ( ) पता नही ( )

5. क्या मरीज को उलटी होती थी?
S हाँ (----------------------) नहीं ( ) पता नही ( )

6. प्यास कैसी थी?
C बढ़ गई थी (----------------------) नहीं ( ) पता नही ( )

7. क्या ऑखे गहरी अंदर गई थी?
C हाँ (----------------------) नहीं ( ) पता नही ( )

8. आखरी दो दिनमें कितनी पेशाब होती थी?
C कम|कुछ भी नहीं (----------------------) हमेशा जैसी ( ) पता नही ( )

9. पेशाब का रंग कैसा था?
C गर्द या बहोत पिली (----------------------) हमेशा जैसी ( ) पता नही ( )

10. क्या मरीज को दस्त के समय पानी/ शरबत/ जीवनरक्षक घोल पिलाना शुरु रखा था?
बहोत (----------------------) S कम नहीं ( ) पता नही ( )

**पतली दस्त**
संभव = 1E
बहुतेक = 1E + 2C या 1E + 1C + 2S

V) रॅबीज

1. क्या मरीज को कुत्ते, जंगली जानवर ने, कटा था?
E हाँ (----------------------) नहीं ( ) पता नही ( )

2. क्या मरीज मरने से कुछ दिन पहले पानी पीनेसे घबराता या डरता था? डर के वजह से पानी नहीं पिता था?
C हाँ (----------------------) नहीं ( ) पता नही ( )

3. क्या मरीज मरने से पहले हवा से डरता था?
C हाँ (----------------------) नहीं ( ) पता नही ( )
* यदी 1 भी हाँ होगा तो 4 से 11 तक सवाल पुछीये|
* यदी सभी नहीं होंगे तो VII पर जाइये|

4. क्या मरीज मरने से पहले पागल जैसे करता था? काटता था?
C हाँ (----------------------) नहीं ( ) पता नही ( )

5. क्या मरीज मरने के कुछ घंटे बहोश हुआ था या बिलकुल शांत हुआ था?
S हाँ (----------------------) नहीं ( ) पता नही ( )

6. क्या मरीज को जिस जगह कुत्ते ने कटा था वहाँ इज आग/जलन/स्पर्शवेदना होना या स्नायु कि हलचल थी?
C हाँ (----------------------) नहीं ( ) पता नही ( )

7. क्या मरीज के मुँह से फेस निकलता था?
C हाँ (----------------------) नहीं ( ) पता नही ( )

8. प्रकाश, स्पर्श या आवाज से क्या मरीज डरता था?
S हाँ (----------------------) नहीं ( ) पता नही ( )

9. क्या मरीज बिच-बिचमें मानसिक रुपसे विकलांग होता था?
S हाँ (----------------------) नहीं ( ) पता नही ( )

10. क्या मरीज को दो-दो चीजे दिखती थी? क्या मरीज का चेहरा तिरछा था?
S हाँ (----------------------) नहीं ( ) पता नही ( )

11. क्या मरीज को निगलने में दिक्कत होती थी?
S हाँ (----------------------) नहीं ( ) पता नही ( )

**रॅबीज**
संभव = 1E + 1C / 1C +2S
बहुतेक = 1E + 3C या 1E + 2C + 2S/3C+3S

VII) एड्स

1. क्या एक माह् से ज्यादा दिन तक बुखार था?
E हाँ (----------------------) नहीं ( ) पता नही ( )

2. क्या मरीज का वजन आखरी छ माह् में १०% (दस प्रतिशत) से ज्यादा कम हुआ? (सुखी की बिमारी थी?
E हाँ (----------------------) नहीं ( ) पता नही ( )

3. क्या मरीज को मरने से पह्ले एक माह् से ज्यादा समय से लगातार पतली दस्त शुरु थी?
E हाँ (----------------------) नहीं ( ) पता नही ( )

4. क्या मरीज को 1 माह् से ज्यादा समय तक खाँसी थी?
S हाँ (----------------------) नहीं ( ) पता नही ( )

5. क्या मरीज को नागिन की बिमारी (चमड़ी पर अंगार,लालसर चट्टे और पानीवाले फोड़े) थी?
C हाँ (----------------------) नहीं ( ) पता नही ( )

6. क्या मरीज के जबान (जीभ) पर सफ़ेद दाग (बुरशी) आयी थी?
C हाँ (----------------------) नहीं ( ) पता नही ( )

7. क्या मरीज को टी.बी. की बिमारी थी?
C हाँ (----------------------) नहीं ( ) पता नही ( )

8. क्या मरीज को पेशाब की जगह ह्मेशा फोड़ा/फुंसी होती थी?
S हाँ (----------------------) नहीं ( ) पता नही ( )

9. क्या मरीज के पेशाब से सफ़ेद पिप जाता था?
S हाँ (----------------------) नहीं ( ) पता नही ( )

10. क्या मरीज के शारीर पर बह्रोत गठाने आयी थी?
C हाँ (----------------------) नहीं ( ) पता नही ( )

11. क्या मरीज वेश्या के साथ या विवाहबाह्य संभोग करता था?
S हाँ (----------------------) नहीं ( ) पता नही ( )

12. क्या मरीज को मरने के एक साल पहले कभी खुन लगाया/चढ़ाया था?
S हाँ (----------------------) नहीं ( ) पता नही ( )

13. क्या मरीज के पती/पत्नी को या बच्चे को एड्स की बिमारी है?
S हाँ (----------------------) नहीं ( ) पता नही ( )

14. क्या मरीज को पीलिया था?
S हाँ (----------------------) नहीं ( ) पता नही ( )

15. क्या मरीज को भूख नहीं लगती थी?
S हाँ (----------------------) नहीं ( ) पता नही ( )

एड्स

संभव = 2E + 2S या 2E + 1C

बहुतेक = 3E + 1C या 3E + 2S

या 2E+2C+3S अथवा 2E+1C +5S

VII) धनुर्वात

1. क्या मरीज को झटके/फीट (दाटी) आये थे? कब?
C हाँ (----------------------) नहीं ( ) पता नही ( )

2. क्या मरीज का शरीर कड़क हुआ था? और धनुष्यबान जैसे पीछे झुका?
C हाँ (----------------------) नहीं ( ) पता नही ( )

3. क्या मरीज मुँह हमेशा जैसे पूरी तरह से खोल नहीं सकता था?
हाँ (----------------------) E नहीं (----------------------) पता नही ( )
क्या मरीज की दातखील लगातार बैठी?
E हाँ (----------------------) नहीं ( ) पता नही ( )

एक भी बिमारी का लक्षण नहीं होंगा तो VIII पर जाईये| और एक भी बिमारी का लक्षण होगा तो सवाल से 4 से 14 तक पूछिये|

4. क्या मरीज के झटके आवाज या प्रकाश बढ़ते थे?
S हाँ (----------------------) नहीं ( ) पता नही ( )

5. क्या मरीज आखरी क्षण तक होश में था?
C हाँ (----------------------) नहीं ( ) पता नही ( )

6. क्या मरीज को लोहे की चिज (जंग खायी हई)/ काटा चुभा था? या मरीज को चोट आकर जखम गंदी हुई थी? क्या गर्भवती महीला की जचकी के समय नल गंदी अवजार से काटी थी? काटने के बाद उस जगह पर गोबर लगाया था?
S हाँ (----------------------) नहीं ( ) पता नही ( )

7. क्या लोग इस बिमारी को धनुर्वाद बोलते है|
S हाँ (----------------------) नहीं ( ) पता नही ( )

8. क्या मरीज को धनुर्वाद के टिके नहीं मिले थे?
हाँ (----------------------) E नहीं ( ) पता नही ( )

9. क्या मरीज को निगलने में तकलीफ होती थी?
S हाँ (----------------------) नहीं ( ) पता नही ( )

10. क्या मरीज का पेट बहुत कड़क था?
S हाँ (----------------------) नहीं ( ) पता नही ( )

11. क्या मरीज का चेहरा पुतले के जैसे हुआ था?
S हाँ (----------------------) नहीं ( ) पता नही ( )

12. क्या मरीज का चेहरा, सिना, कमर, पेट कड़क थे और हाथ पैर सही थे?
S हाँ (----------------------) नहीं ( ) पता नही ( )

13. क्या मरीज का शरीर का कोई भाग जल गया था? कान में से पिप बहता था? ऑपरेशन हुआ था? मरने के पहले (15 दिन के अंदर) जचकी हुई थी? निर्जतुक नहीं कियें हुयें इंजेक्शन लेने की आदत थी?
S हाँ (----------------------) नहीं ( ) पता नही ( )

धनुर्वाद
संभव = 1E + 1C अथवा 1C + 2S
बहुतेक = 1E + 2C + 3S + 2S अथवा 3C+3S

VIII) पिलीया/कावीळ

1. क्या मरीज की आँखे पिली थी?
E हाँ (----------------------) नहीं ( ) पता नही ( )

2. क्या मरीज की पेशाब पिली थी?
E हाँ (----------------------) नहीं ( ) पता नही ( )

3. क्या मरीज को उलटी जैसे लगता था?
S हाँ (----------------------) नहीं ( ) पता नही ( )

4. क्या मरीज को बार –बार उलटीया होती थी?
S हाँ (----------------------) नहीं ( ) पता नही ( )

5. क्या मरीज को बुखार था?
S हाँ (----------------------) नहीं ( ) पता नही ( )

6. क्या मरीज के पेट दर्द था?
S हाँ (----------------------) नहीं ( ) पता नही ( )

7. क्या मरीज को भुख नहीं लगती थी?
S हाँ (----------------------) नहीं ( ) पता नही ( )

8. क्या मरीज का वजन कम हो रहा था?
S हाँ (----------------------) नहीं ( ) पता नही ( )

9. क्या मरीज को मरने के एक माह पहले खुन लगाया?
S हाँ (----------------------) नहीं ( ) पता नही ( )

10. क्या मरीज को बहोत दारू पिने की आदत थी?
S हाँ (----------------------) नहीं ( ) पता नही ( )

11. क्या पेट में गोला था?
S हाँ (----------------------) नहीं ( ) पता नही ( )

पिलीया
संभव = 1E
बहुतेक = 2E + 3S/ 1E +5S

IX) मेंदू (मस्तिष्क) पर सुजन

1. क्या मरीज बहोत सुस्त या बेहोश था?
नहीं ( ) S हाँ(----------------------) पता नही ( )

2. क्या मरीज को झटके/दाटी आई थी?
C हाँ (----------------------) नहीं ( ) पता नही ( )

3. क्या मरीज को बहोत (निरर्थक) करता था? या पागल जैसे बाते करता था?
नहीं ( ) C हाँ(----------------------) पता नही ( )

4. क्या मरीज को बुखार था?
E हाँ (----------------------) नहीं ( ) पता नही ( )

5. क्या बुखार के साथ पसीना आता था?
S हाँ (----------------------) नहीं ( ) पता नही ( )

6. क्या बुखार के साथ पसीना आता था?

S हाँ (----------------------) नहीं ( ) पता नही ( )

7. क्या लगातार सिरदर्द और उलटियाँ होती थी?

S हाँ (----------------------) नहीं ( ) पता नही ( )

8. क्या प्रकश मरीज को सहन नहीं होता था? आँखोंपर प्रकाश डालने के बाद वह तुरंत आँख बंद कर लेता था?

S हाँ (----------------------) नहीं ( ) पता नही ( )

9. क्या मरीज के कान से पिप निकलता था?

S हाँ (----------------------) नहीं ( ) पता नही ( )

10. क्या गर्दन कड़क या ताठ रहती थी? या गर्दन पीछे पीछे जाती थी?

S हाँ (----------------------) नहीं ( ) पता नही ( )

11. क्या मरीज को बुखार के साथ खाँसी,दम, पेशाब में जलन पेट में दर्द, इत्यादी होत्ता था?

S हाँ (----------------------) नहीं ( ) पता नही ( )

पिलीया

संभव = 1E +1C /1E + 5S

बहुतेक = 1E + 2C या 1E +1C+5S

12. क्या मरीज का एक हाँथ और एक पैर कमजोर हुआ था? (वह हाँथ,पैर हिला नहीं सकता था?)

C हाँ (----------------------) नहीं ( ) पता नही ( )

13. क्या मरीज का मुँह तेढा हुआ था?

C हाँ (----------------------) नहीं ( ) पता नही ( )

CVE (मस्तिष्क में खुन की बिमारी)

संभव = 1C

बहुतेक = 3C (12 और 13 के C मिलकर)

X) ॲनेमिया (खुन की कमतरता)

1. क्या मरने के पहले मरीज की जबान,ओठ, आँखे की पलके (अंदरसे), हाथ के तलवे, नाखुन बैगरे सफ़ेद हुए थे? (लाल या गुलाबी नहीं थे?)

E हाँ (----------------------) नहीं ( ) पता नही ( )

2. क्या मरने कुछ दिन पहले से मरीज के शरीर के किसी भी अवयवसे (चमड़ी,तट्टी, पेशाब, उलटी,बच्चेदानी) खुन बह रहा था? क्या चमड़ीमें खुन के दाग (लाल, हरे, नीले) थे? क्या डाम्बर जैसी कलि, चिकट तट्टी हुई थी?

C हाँ (----------------------) नहीं ( ) पता नही ( )

3. क्या मरीज को बहोत कमजोरी (थकान) महसुस होती थी?

S हाँ (----------------------) नहीं ( ) पता नही ( )

4. क्या मरीज थोडासा चलने पर या काम करने पर सॉस फुलती थी?

S हाँ (----------------------) नहीं ( ) पता नही ( )

5. क्या मरीज को खुन की कमतरता की बिमारी बतायी गई थी?

S हाँ (----------------------) नहीं ( ) पता नही ( )

6. क्या मरीज को बार-बार खुन लगाने की (चढ़ाने की) जरुरत पड़ती थी?

C हाँ (----------------------) नहीं ( ) पता नही ( )

7. क्या मरीज के तट्टी में (कृमि) गिरते थे?

S हाँ (----------------------) नहीं ( ) पता नही ( )

8. क्या मरीज को सिकलसेल (जोड़ो के दर्द) की बिमारी थी?
S हाँ (----------------------) नहीं ( ) पता नही ( )

**अॅनेमिया**
संभव = 1E + 2S / अथवा 1C + 2S
बहुतेक = 1E = 1C+2S अथवा 1E + 2C अथवा 2C + 2S अथवा 1C+3S

XI) मुहँ का कँन्सर या कर्करोग

1. क्या मुहँ में ठिक न होनेवाली दर्दरहित जखम (फोड़ा/उज्जु) बहोत दिन से थी|
E हाँ (----------------------) नहीं ( ) पता नही ( )

2. क्या मुहँ में ठिक न होनेवाली दर्दरहित कोई गठान आयी थी|
E हाँ (----------------------) नहीं ( ) पता नही ( )

3. क्या मुहँ के गठान या जखम से खाना निगलने में कोई तकलीफ थी|
S हाँ (----------------------) नहीं ( ) पता नही ( )

4. क्या मुहँ के गठान या जखम बहोत गंदा बास आता था?
S हाँ (----------------------) नहीं ( ) पता नही ( )

5. क्या मरीज के मुहँ में सफ़ेद दाग (-------) आया और फिर गठान| फोड़ा जखम तैयार हुई|
C हाँ (----------------------) नहीं ( ) पता नही ( )

सब नहीं होंगा XII पर जाइये| एक भी हाँ होंगा तो सवाल 6 से 9 तक पूछीयें|

6. क्या मरीज का वजन दिन ब दिन कम होने जा रहा था? (क्या मरीज दिन ब दिन सुख रहा था?)
C हाँ (----------------------) नहीं ( ) पता नही ( )

7. क्या मरीज की भुख बहोत कम हुई थी?
S हाँ (----------------------) नहीं ( ) पता नही ( )

8. क्या मरीज के जखम से खुन बहता था?
S हाँ (----------------------) नहीं ( ) पता नही ( )

9. क्या मरीज को तम्बाखु, बीडी या चिलम, दारू सेवन करने की आदत थी?
S हाँ (----------------------) नहीं ( ) पता नही ( )
यदी हाँ तो कौनसी आदत थी? (----------------------------)

**मुहँ का कँन्सर**
संभव = 1E + 1S
बहुतेक = 1E = 1C+1S अथवा 1E + 4S OR 2E + 2S

XII) क्या और दुसरी कोई तकलीफ थी?

कौनसी

____

____

____

____

____

**अन्य --**   **--**

**वजह**	**पता**	**नहीं**

ईलाज किया क्या?

हाँ ( ) नहीं ( ) पता नहीं ( )

यदी हाँ तो,

किसने --

कहाँ --

क्या ईलाज किया --

बिमारी कौनसी बताई? --

पर्यवेक्षक/मार्गदर्शक का निष्कर्ष

1) --

2) --

3) --

4) --

5) --

1.

**मृतक के**	**नाम**	**सही / अंघुठा**
माता		
पिता		
पति		
पत्नि		
लड़का		
लड़की		
(किसी दो का)		

सही / अंघुठा

2. सरपंच का नाम:-- --

सही / अंघुठा

3. पोलीस पटेल का नाम: -- --

सही / अंघुठा

मार्गदर्शक / पर्यवेक्षक का नाम: -- --

दिनांक : / / २०

MAHAN TRUST KARMGRAM UTAVALI, MELGHAT

पहचान क्रमांक ( )( )( ) – ( )( )( ) – ( )( )( )( )( ) – ( )( )

१) मृतक का पूरा नाम : ________________ पिता का नाम __________________ जाटो ________________

२) जन्मतारीख (पता है तो): ( ) ( )/ ( ) ( )/ ( )( )( )( ) (दिन /महिना/साल) ३) लिंग: पुरुष ( )स्त्री ( )

४) गांव का नाम (हमेशा रहनेका) : _____________ ५) जहाँ मृत्यू हुआ वह गाँव का नाम : _____________

६) मरने कि तारीख : ( ) ( )/ ( ) ( )/ ( )( )( )( ) (दिन /महिना/साल) ७)मृत्यू के समय उम्र: ( )( )( )

	Inference on causes of death	Possible	Most probable	Disease code
1)	Pneumonia			
2)	Tuberculosis			
3)	COAD			
4)	IHD			
5)	Dysentry			
6)	Chronic Diarrhoea			
7)	Acute Diarrhoea			
8)	Rabies			
9)	AIDS			
10)	Tetanus			
11)	Jaundice			
12)	CNS Infection			
13)	CVE			
14)	Fever Cause Unknown			
15)	Anemia			
16)	Oral Cancer / Malignacy			
17)	Other			
18	Cause not Known			

Underlying Cause ______________________________( )______________

Singnature: _______________________

Name: ___________________________

Date: _____/___/________
