# Supplementary material for: Community—Minimal Invasive Tissue Sampling (cMITS) using a modified ambulance for ascertaining the cause of death: A novel approach piloted in a remote inaccessible rural area in India
Source: Arch Public Health. 2023 Apr 27;81:72. doi: 10.1186/s13690-023-01062-x (PMC10134564; doi:10.1186/s13690-023-01062-x)
Supplement: Supplementary file 9 — Additional file 9: Annexure 9: Tangerine Software. [file 13690_2023_1062_MOESM9_ESM.pdf]

(1st May 2020 to 30th April 2021) (Dharni Block of Amaravati district)

About Tangerine

Tangerine is open source electronic data collection software designed for use on Android mobile devices. Its primary use is to enable recording of students' responses in oral early grade reading and mathematics skills assessments. Tangerine is also used to capture interview responses from students, teachers, and principals; as well as in simple surveys and other data collections.

Tangerine was developed by RTI International with funding from RTI and Google.org.

Objective

The application will be used for online capturing of data on MITS from the field, which will be available to all stakeholders from time to time, ensuring proper security.

Tangerine code base

All of Tangerine's code base is stored in its public repository on GitHub. The repository can be found at https://github.com/Tangerine-Community/Tangerine. To be able to check out the open source project code you need to have a git client installed on your computer. Another prerequisite for Tangerine is to have docker installed.
