# Supplementary material for: Community—Minimal Invasive Tissue Sampling (cMITS) using a modified ambulance for ascertaining the cause of death: A novel approach piloted in a remote inaccessible rural area in India
Source: Arch Public Health. 2023 Apr 27;81:72. doi: 10.1186/s13690-023-01062-x (PMC10134564; doi:10.1186/s13690-023-01062-x)
Supplement: Supplementary file 10 — Additional file 10: Annexure 10: COD procedure. [file 13690_2023_1062_MOESM10_ESM.pdf]

Annexure 10: Procedure of determination of cause of death (CoD) :

The process of assigning the cause of death was done by the DeCoDe panel. For diagnosis standards CHAMPS data elements were used. We used De-Code panel case report form (WHO international death certificate). To determine the sequence of diagnoses leading to death, we used clinical judgement, verbal autopsy (VA), microbiology, MITS PCR, MITS Histopathology, and ICD-10 coding standards. We have classified causes of deaths as: Immediate cause, most probable cause, underlying cause and possible cause. Three levels of certainties were used for diagnostic standards.

Procedure of determination of cause of death (CoD) :

DeCoDe panel was formed. The panel consisted of

- 1) Dr. Ashish Satav. Physician providing medical services to tribal area of Melghat since 25 years.
- 2) Dr. Eric Simoes, Paediatrician, Infectious disease expert.
- 3) Dr. Vibhawari Dani. Paediatrician and research head
- 4) Dr. Niteen Wairagkar: Community Physician, public health and global health expert.
- 5) Dr. Pradyot Garge Pathologist
- 6) Dr. Yagnesh Thakar: Microbiologist
- 7) Dr. Dhananjay Raje: Biostatistician.

The DeCoDe panel was responsible for assigning the causes of deaths.

For diagnosis standards, we used CHAMPS data elements as follows.

Type of data	Specific data element
Case data extraction	Maternal data
	Child clinical data (including antemortem diagnostics found in medical records)
Verbal autopsy	Narrative and list of conditions
MITS procedure findings	Photography and gross findings, Measurements
Pathology	Histology of postmortem biopsies (lung, brain, liver)<br>Tissue PCR (lung, brain, liver)<br>Placenta histology (SB, Early newborn deaths)<br>Immunohistochemistry only if must and if sufficient funds will be available.

Post-mortem diagnostics	Microbiology/culture (blood, CSF)
	Molecular (TAC) (blood, CSF, NP/OP swab, lung tissue)
	HIV testing
	TB testing
	Malaria testing

<u>Diagnosis standards:</u>

- We will use De-Code panel case report form (WHO international death certificate)
- We will establish diagnoses which will contribute to a person's death.

➢ To determine the sequence of diagnoses leading to death, we will use clinical judgement, verbal autopsy (VA), ICD-10 coding standards.

➢ We have classified causes as: Immediate cause, most probable cause, underlying cause and possible cause.

- **<u>Level of certainty which will be used for diagnosis standards:</u>**
- Level 1= Diagnosis with highest level of certainty

a) Highly specific pathological findings, Or

b) lab test with specific findings and medically observed and documented/ clinically observed appropriate illness signs.

- Level 2= Diagnosis with a high level of certainty,

a) medically observed and documented appropriate illness sign(s) to support the diagnosis. Or

b) a CHAMPS laboratory test with specific findings and supporting symptoms reported by Verbal Autopsy (VA).

- Level 3= Conditions which would be considered for diagnosis (but do not meet level 1 or 2 criteria.

i.e. from Verbal Autopsy data alone.
