# Supplementary material for: Community—Minimal Invasive Tissue Sampling (cMITS) using a modified ambulance for ascertaining the cause of death: A novel approach piloted in a remote inaccessible rural area in India
Source: Arch Public Health. 2023 Apr 27;81:72. doi: 10.1186/s13690-023-01062-x (PMC10134564; doi:10.1186/s13690-023-01062-x)
Supplement: Supplementary file 11 — Additional file 11. [file 13690_2023_1062_MOESM11_ESM.pdf]

Parlberg, Lindsay 2:12 AM (4 hours ago)
to me, Norman, MITSAllianceGrants

Parlberg, Lindsay <lparlberg@rti.org>

to: Ashish Satav <drashish@mahantrust.org>, "Goco, Norman" <ngoco@rti.org>, MITSAllianceGrants <MITSAllianceGrants@rti.org>

date: Jan 7, 2023, 2:12 AM

subject: RE: Permission to use photos of MITS SOP.

Hi Ashish,
Happy New Year! Thanks for reaching out with this request. The use is permissible as long as the following two conditions are upheld:

1. Ensure “**Photo credit attributed to Alex G. Kamweru on behalf of the MITS Surveillance Alliance”**

and

2. Ensure the face is obscured in any photos showing someone's face and there is NO way to discern the identity of the individual in the photo

All the best,
Lindsay

Lindsay Parlberg
Pronouns: she/her
RTI International
Research Public Health Analyst

Center for Applied Public Health Research
Biostatistics and Epidemiology Division
3040 E. Cornwallis Rd.

Research Triangle Park, NC 27709
